# Physical activity, diet and other behavioural interventions for improving cognition and school achievement in children and adolescents with obesity or overweight

**DOI:** 10.1002/14651858.CD009728.pub4

**Published:** 2018-03-02

**Authors:** Anne Martin, Josephine N Booth, Yvonne Laird, John Sproule, John J Reilly, David H Saunders

**Affiliations:** University of EdinburghCentre for Population Health SciencesMedical School, Teviot PlaceEdinburghUKEH8 9AG; University of GlasgowMRC/CSO Social and Public Health Sciences Unit200 Renfield StreetGlasgowUKG2 3QB; The University of EdinburghInstitute for Education, Community and SocietyMoray House School of EducationRoom 2.17, St John's LandEdinburghUKEH8 8AQ; University of EdinburghScottish Collaboration for Public Health Research and Policy (SCPHRP)20 West Richmond StreetEdinburghUKEH8 9DX; Institute for Sport, Physical Education and Health Sciences (SPEHS), University of EdinburghMoray House School of EducationHolyrood RoadEdinburghEH8 8AQUK; University of StrathclydePhysical Activity for Health Group, School of Psychological Sciences and Health50 George StreetGlasgowUKG1 1QE; University of EdinburghPhysical Activity for Health Research Centre (PAHRC)St Leonards LandHolyrood RoadEdinburghMidlothianUKEH8 8AQ

**Keywords:** Adolescent, Child, Humans, Achievement, Diet, Educational Status, Executive Function, Exercise, Life Style, Mathematics, Overweight, Overweight/psychology, Overweight/therapy, Pediatric Obesity, Pediatric Obesity/psychology, Pediatric Obesity/therapy, Randomized Controlled Trials as Topic, Reading, Sensitivity and Specificity

## Abstract

**Background:**

The global prevalence of childhood and adolescent obesity is high. Lifestyle changes towards a healthy diet, increased physical activity and reduced sedentary activities are recommended to prevent and treat obesity. Evidence suggests that changing these health behaviours can benefit cognitive function and school achievement in children and adolescents in general. There are various theoretical mechanisms that suggest that children and adolescents with excessive body fat may benefit particularly from these interventions.

**Objectives:**

To assess whether lifestyle interventions (in the areas of diet, physical activity, sedentary behaviour and behavioural therapy) improve school achievement, cognitive function (e.g. executive functions) and/or future success in children and adolescents with obesity or overweight, compared with standard care, waiting‐list control, no treatment, or an attention placebo control group.

**Search methods:**

In February 2017, we searched CENTRAL, MEDLINE and 15 other databases. We also searched two trials registries, reference lists, and handsearched one journal from inception. We also contacted researchers in the field to obtain unpublished data.

**Selection criteria:**

We included randomised and quasi‐randomised controlled trials (RCTs) of behavioural interventions for weight management in children and adolescents with obesity or overweight. We excluded studies in children and adolescents with medical conditions known to affect weight status, school achievement and cognitive function. We also excluded self‐ and parent‐reported outcomes.

**Data collection and analysis:**

Four review authors independently selected studies for inclusion. Two review authors extracted data, assessed quality and risks of bias, and evaluated the quality of the evidence using the GRADE approach. We contacted study authors to obtain additional information. We used standard methodological procedures expected by Cochrane. Where the same outcome was assessed across different intervention types, we reported standardised effect sizes for findings from single‐study and multiple‐study analyses to allow comparison of intervention effects across intervention types. To ease interpretation of the effect size, we also reported the mean difference of effect sizes for single‐study outcomes.

**Main results:**

We included 18 studies (59 records) of 2384 children and adolescents with obesity or overweight. Eight studies delivered physical activity interventions, seven studies combined physical activity programmes with healthy lifestyle education, and three studies delivered dietary interventions. We included five RCTs and 13 cluster‐RCTs. The studies took place in 10 different countries. Two were carried out in children attending preschool, 11 were conducted in primary/elementary school‐aged children, four studies were aimed at adolescents attending secondary/high school and one study included primary/elementary and secondary/high school‐aged children. The number of studies included for each outcome was low, with up to only three studies per outcome. The quality of evidence ranged from high to very low and 17 studies had a high risk of bias for at least one item. None of the studies reported data on additional educational support needs and adverse events.

Compared to standard practice, analyses of physical activity‐only interventions suggested high‐quality evidence for improved mean cognitive executive function scores. The mean difference (MD) was 5.00 scale points higher in an after‐school exercise group compared to standard practice (95% confidence interval (CI) 0.68 to 9.32; scale mean 100, standard deviation 15; 116 children, 1 study). There was no statistically significant beneficial effect in favour of the intervention for mathematics, reading, or inhibition control. The standardised mean difference (SMD) for mathematics was 0.49 (95% CI ‐0.04 to 1.01; 2 studies, 255 children, moderate‐quality evidence) and for reading was 0.10 (95% CI ‐0.30 to 0.49; 2 studies, 308 children, moderate‐quality evidence). The MD for inhibition control was ‐1.55 scale points (95% CI ‐5.85 to 2.75; scale range 0 to 100; SMD ‐0.15, 95% CI ‐0.58 to 0.28; 1 study, 84 children, very low‐quality evidence). No data were available for average achievement across subjects taught at school.

There was no evidence of a beneficial effect of physical activity interventions combined with healthy lifestyle education on average achievement across subjects taught at school, mathematics achievement, reading achievement or inhibition control. The MD for average achievement across subjects taught at school was 6.37 points lower in the intervention group compared to standard practice (95% CI ‐36.83 to 24.09; scale mean 500, scale SD 70; SMD ‐0.18, 95% CI ‐0.93 to 0.58; 1 study, 31 children, low‐quality evidence). The effect estimate for mathematics achievement was SMD 0.02 (95% CI ‐0.19 to 0.22; 3 studies, 384 children, very low‐quality evidence), for reading achievement SMD 0.00 (95% CI ‐0.24 to 0.24; 2 studies, 284 children, low‐quality evidence), and for inhibition control SMD ‐0.67 (95% CI ‐1.50 to 0.16; 2 studies, 110 children, very low‐quality evidence). No data were available for the effect of combined physical activity and healthy lifestyle education on cognitive executive functions.

There was a moderate difference in the average achievement across subjects taught at school favouring interventions targeting the improvement of the school food environment compared to standard practice in adolescents with obesity (SMD 0.46, 95% CI 0.25 to 0.66; 2 studies, 382 adolescents, low‐quality evidence), but not with overweight. Replacing packed school lunch with a nutrient‐rich diet in addition to nutrition education did not improve mathematics (MD ‐2.18, 95% CI ‐5.83 to 1.47; scale range 0 to 69; SMD ‐0.26, 95% CI ‐0.72 to 0.20; 1 study, 76 children, low‐quality evidence) and reading achievement (MD 1.17, 95% CI ‐4.40 to 6.73; scale range 0 to 108; SMD 0.13, 95% CI ‐0.35 to 0.61; 1 study, 67 children, low‐quality evidence).

**Authors' conclusions:**

Despite the large number of childhood and adolescent obesity treatment trials, we were only able to partially assess the impact of obesity treatment interventions on school achievement and cognitive abilities. School and community‐based physical activity interventions as part of an obesity prevention or treatment programme can benefit executive functions of children with obesity or overweight specifically. Similarly, school‐based dietary interventions may benefit general school achievement in children with obesity. These findings might assist health and education practitioners to make decisions related to promoting physical activity and healthy eating in schools. Future obesity treatment and prevention studies in clinical, school and community settings should consider assessing academic and cognitive as well as physical outcomes.

## Plain language summary

**Healthy weight interventions for improving thinking skills and school performance in children and teenagers with obesity**

**What is the aim of this review?**

The aim of this Cochrane Review was to find out if healthy weight interventions can improve thinking skills and school performance in children and teenagers with obesity. Cochrane researchers collected and analysed all relevant studies to answer this question.

**What are the key messages?**

This updated review provides some evidence that school programmes that encourage healthier child weight may also provide ‘co‐benefits’ of thinking skills and school performance. However, we need more high‐quality healthy‐weight interventions that test thinking skills and school performance, as well as health outcomes.

**What was studied in this review?**

The number of children and teenagers with obesity is high worldwide. Some children and teenagers with obesity have health issues or are bullied because of their body weight. These experiences have been linked to problems in performing well in school, where they tend to perform less well in thinking tasks such as problem‐solving. Physical activity and healthy eating benefit a healthy body weight and improve thinking skills and school performance in children with a healthy weight. Studies found that healthy‐weight interventions can reduce obesity in children and teenagers, but it is unknown if and how well healthy‐weight interventions can improve thinking skills and school performance in children and teenagers with obesity.

**What are the main results of this review?**

The review authors found 18 studies which included a total of 2384 children and teenagers with obesity. Five studies assigned individual children to intervention or control groups. Thirteen studies allocated entire classes, school or school districts to the intervention and control group. Of the 18 studies, 11 involved children at primary/elementary‐school age. Eight studies offered physical activity interventions, seven studies combined physical activity programmes with healthy lifestyle education, and three studies offered dietary changes. The studies took place in 10 different countries. Seventeen studies had at least one flaw in how the study was done. This reduces the level of confidence we can have in the findings.

Few studies shared the same type of school performance or thinking skills. Only three studies reported the same outcome. None of the studies reported on additional educational support needs and harmful events. We found that, compared with usual routine, physical activity interventions can lead to small improvements in problem‐solving skills. This finding was based on high‐quality evidence. Moderate‐quality findings showed that physical activity interventions do not improve mathematics and reading achievement in children with obesity. Very low‐quality evidence also suggested no benefits of physical activity interventions for improving uncontrolled behavioural responses. General school achievement was not reported in studies comparing physical activity interventions with standard practice.

Studies that compared physical activity interventions plus healthy lifestyle education with standard practice were of low to very low quality. They showed no improvement in school achievement or uncontrolled behavioural responses in the intervention group compared to the control group. Problem‐solving skills were not reported in studies comparing physical activity plus healthy lifestyle education with standard practice.

Our findings indicate that changing knowledge about nutrition, and changing the food offered in schools can lead to moderate improvements in general school achievement of teenagers with obesity, when compared to standard school practice. Replacing packed school lunch with a nutrient‐rich diet plus nutrition education did not improve mathematics and reading achievement of children with obesity. However, the quality of evidence for general school achievement, mathematics and reading was low. This means that future research is very likely to change the results, because included studies showed some methodological weaknesses (for example, small numbers of children and a high dropout of children from studies). Problem‐solving skills and uncontrolled behavioural responses were not reported for dietary intervention studies.

**How up‐to‐date is this review?**

The review authors searched the scientific literature for relevant studies in February 2017.

## Summary of findings

**Summary of findings for the main comparison:** Physical activity intervention compared to standard practice for improving cognition and school achievement in children and adolescents with obesity or overweight

**Physical activity interventions compared to standard practice for improving cognition and school achievement in children and adolescents with obesity or overweight**						
**Patient or population:** Children and adolescents with obesity or overweight **Setting:** Classroom and school environment or as after‐school activity in the USA, Norway, Spain, and The Netherlands **Intervention:** Physical activity interventions (active academic lessons, extracurricular games, after‐school group exercise) **Comparison:** Standard practice (e.g. usual Physical Education curriculum)						
**Outcomes**	Illustrative comparative risks* (95% CI)		Relative effect (95% CI)**	**№ of participants (studies)**	**Quality of the evidence (GRADE)**	**Comments**
	**Assumed risk** **Standard practice **	**Corresponding risk** **Physical activity **				
**School achievement:** **Average achievement across subjects taught at school**	‐	‐	‐	(0 studies)	‐	No data available
**School achievement: Mathematics** Assessed with: standardised national tests, BADyG‐I (numerical quantitative concepts) Follow‐up: range 13 weeks to 1 year immediately post‐intervention	‐	Compared to the control group, the mean mathematics achievement score in the intervention group was**0.49 standard deviations higher** (0.04 lower to 1.01 higher)	‐	255 (2 RCTs)	⊕⊕⊕⊝ **Moderate^1^**	A standard deviation of 0.49 represents a moderate difference between groups
**School achievement: Reading** Assessed with: WJ‐II test of achievement, standardised national tests Follow‐up: range 13 weeks to 7 months immediately post‐intervention	‐	Compared to the control group, the mean reading achievement score in the intervention group was **0.10 standard deviations higher** (0.30 lower to 0.49 higher)	‐	308 (2 RCTs)	⊕⊕⊕⊝ **Moderate^1^**	A standard deviation of 0.10 represents a small difference between groups
**School achievement: Additional educational support needs**	‐	‐	‐	(0 studies)	‐	No data available
**Cognitive function: Composite executive functions** Assessed with: CAS Follow‐up: 13 weeks immediately post‐intervention	The mean composite executive functions score in the control group was **102 scale points**	The mean composite executive functions score in the intervention group was **5.00 points higher** (0.68 higher to 9.32 higher)	‐	116 (1 RCTs)	⊕⊕⊕⊕ **High**	‐
**Cognitive function: Inhibition control** Assessed with: SCWT, scale range: 0 to 100 Follow‐up: mean 18 months immediately post‐intervention	The mean inhibition control score in the control group was **20.55 scale points**	The mean inhibition control score in the intervention group was **1.55 points lower** (5.85 lower to 2.75 higher)	‐	84 (1 RCT)	⊕⊝⊝⊝ **Very Low^2^**	‐
**Adverse events**	‐	‐	‐	(0 studies)	‐	No data available
*The effect sizes are differences in standard deviations. To facilitate interpretation we have used rules of thumb in interpretation of effect size (section 12.6.2 in Higgins 2011), where a standard deviation of 0.2 represents a small difference between groups, 0.5 represents a moderate difference, and 0.8 represents a large difference. ** Different assessment tools were used to assess school and cognitive outcomes. We therefore calculated standardised mean differences to assess the effect size between intervention and control groups. **WJ**: Woodcock‐Johnson; **SCWT**: Stroop test (colour and words); **CAS**: Das‐Naglieri‐Cognitive Assessment System; **D–KEFS**: Delis‐Kaplan Executive Function System; **BADyG‐I:** [Batería de aptitudes diferenciales y generals] Differential Aptitude Battery‐ General scale. **MD:** Mean difference, **SMD:** Standardised mean difference **CI:** Confidence interval						
**GRADE Working Group grades of evidence** **High quality:** We are very confident that the true effect lies close to that of the estimate of the effect **Moderate quality:** We are moderately confident in the effect estimate: The true effect is likely to be close to the estimate of the effect, but there is a possibility that it is substantially different **Low quality:** Our confidence in the effect estimate is limited: The true effect may be substantially different from the estimate of the effect **Very low quality:** We have very little confidence in the effect estimate: The true effect is likely to be substantially different from the estimate of effect						

^1^Downgraded one level due to high risk of attrition bias. ^2^Downgraded three levels due to high risk of selection bias, attrition bias and imprecision (wide confidence intervals) due to a low sample size.

**Summary of findings 2:** Physical activity plus healthy lifestyle education interventions compared to standard practice for improving cognition and school achievement in children and adolescents with obesity or overweight

**Physical activity plus healthy lifestyle education interventions compared to standard practice for improving cognition and school achievement in children and adolescents with obesity or overweight**						
**Patient or population:** Children and adolescents with obesity or overweight **Setting:** Classroom and school/preschool environment or in another community setting in the USA, Canada, Brazil, Spain, Germany, and Denmark **Intervention:** Physical activity plus healthy lifestyle education interventions **Comparison:** Standard practice (e.g. usual physical education/health education curriculum), and attention control (short‐term, less intensive programme)						
**Outcomes**	Illustrative comparative risks* (95% CI)		Relative effect (95% CI)**	**№ of participants (studies)**	**Quality of the evidence (GRADE)**	**Comments**
	**Assumed risk** **Standard practice **	**Corresponding risk** **Physical activity plus healthy lifestyle education**				
**School achievement:** **Average achievement across subjects taught at school** Assessed with: CAT‐3, scale mean 500, SD 70 Follow‐up: 12 months immediately post‐intervention	The mean score for average achievement across subjects taught at school in the control group was **19.50 grade points**	The mean score for average achievement across subjects taught at school in the intervention group was **6.37 grade points lower** (36.83 lower to 24.09 higher)	‐	31 (1 RCT)	⊕⊕⊝⊝ **Low^1^**	‐
**School achievement: Mathematics** Assessed with: CAT‐3, standardised national tests, M‐CAT Follow‐up: range 4 months to 12 months immediately post‐intervention	‐	Compared to the control group, the mean mathematics achievement score in the intervention group was **0.02 standard deviations higher** (0.19 lower to 0.22 higher)	‐	384 (3 RCTs)	⊕⊝⊝⊝ **Very low^2^**	A standard deviation of 0.02 represents a small difference between groups
**School achievement:** **Reading** Assessed with: CAT‐3, R‐CBM Follow‐up: mean 1 year immediately post‐intervention	‐	Compared to the control group, the mean reading achievement score in the intervention group was **0 standard deviations higher** (0.24 lower to 0.24 higher)	‐	284 (2 RCTs)	⊕⊕⊝⊝ **Low^3^**	A standard deviation of zero represents no difference between groups
**School achievement: Additional educational support needs**	‐	‐	‐	(0 studies)	‐	No data available
**Cognitive function: Composite executive functions**	‐	‐	‐	(0 studies)	‐	No data available
**Cognitive function: Inhibition control** Assessed with: SCWT, KiTAP (Go/No‐go) Follow‐up: range 12 months to 13 months immediately post‐intervention	‐	Compared to the control group, the mean inhibition control score in the intervention group was**0.67 standard deviations lower** (1.50 lower to 0.16 higher)	‐	110 (2 RCTs)	⊕⊕⊝⊝ **Low^4^**	A standard deviation of 0.67 represents a moderate difference between groups
**Adverse events**	‐	‐	‐	(0 studies)	‐	No data available
*The effect sizes are differences in standard deviations. To facilitate interpretation we have used rules of thumb in interpretation of effect size (section 12.6.2 in Higgins 2011), where a standard deviation of 0.2 represents a small difference between groups, 0.5 represents a moderate difference, and 0.8 represents a large difference. ** Different assessment tools were used to assess school and cognitive outcomes. We therefore calculated standardised mean differences to assess the effect size between intervention and control groups. **CAT‐3**: Canadian Achievement Test, version 3; **M‐CAT**: Mathematics Concepts and Applications Test; **R‐CBM**: Reading–Curriculum‐Based Measurement; **PPVT III**: Peabody Picture Vocabulary Test, version 3; **SCWT**: Stroop test (colour and words); **KiTAP**: [Kinderversion der Testbatterie zur Aufmerksamkeitsprüfung] Attention test battery for children; **RCFT**: Rey Complex Figure Test; **CI:** Confidence interval; **SMD:** Standardised mean difference						
**GRADE Working Group grades of evidence** **High quality:** We are very confident that the true effect lies close to that of the estimate of the effect **Moderate quality:** We are moderately confident in the effect estimate: The true effect is likely to be close to the estimate of the effect, but there is a possibility that it is substantially different **Low quality:** Our confidence in the effect estimate is limited: The true effect may be substantially different from the estimate of the effect **Very low quality:** We have very little confidence in the effect estimate: The true effect is likely to be substantially different from the estimate of effect						

^1^Downgraded two levels due high risk of bias in attrition and unclear risk of bias for randomisation. ^2^Downgraded three levels due to high risk of bias in sequence generation, blinding of outcome assessors, and attrition; low sample sizes across studies resulting in imprecision; and inconsistent direction of intervention effects. ^3^Downgraded two levels due to high risk of bias in sequence generation, blinding of outcome assessors, and attrition and inconsistent direction of intervention effects. ^4^Downgraded two levels due to high risk of attrition bias; and selective reporting.

**Summary of findings 3:** Dietary interventions compared to standard practice for improving cognition and school achievement in children and adolescents with obesity and overweight

**Dietary interventions compared to control for improving cognition and school achievement in children and adolescents with overweight and obesity**						
**Patient or population:** Children and adolescents with obesity or overweight **Setting:** Classroom and school environment in the USA and Denmark **Intervention:** Dietary interventions **Comparison:** Standard practice (e.g. usual school lunch)/wait‐list control						
**Outcomes**	Illustrative comparative risks* (95% CI)		Relative effect (95% CI)**	**№ of participants (studies)**	**Quality of the evidence (GRADE)**	**Comments**
	**Assumed risk** **Standard practice **	**Corresponding risk** **Dietary intervention**				
**School achievement:** **Average achievement across subjects taught at school** Assessed with: teacher‐assessed grades Follow‐up: range 1 year to 2 years immediately post‐intervention	‐	Compared to the control group, the mean score for average achievement across subjects taught at school was **0.46 standard deviations highe**r (0.25 higher to 0.66 higher) in the intervention group	‐	382 (2 RCTs)	⊕⊕⊝⊝ **Low^1^**	A standard deviation of 0.46 represents a moderate difference between groups
**School achievement: Mathematics** Assessed with: standard national test, scale range 0 to 69 Follow‐up: mean 3 months immediately post‐intervention	The mean change in mathematics achievement score ranged across control groups from **8.00 to 10.70 scale points**	The mean change in mathematics achievement score in the intervention group was **2.18 scale points lower** (5.83 lower to 1.47 higher)	‐	76 (1 RCT)	⊕⊕⊝⊝ **Low^2^**	‐
**School achievement: Reading** Assessed with: standard national test, scale range 0 to 108 Follow‐up: mean 3 months immediately post‐intervention	The mean change in reading achievement score ranged across control groups from **7.40 to 9.20 scale points**	The mean change in reading achievement score in the intervention group was **1.17 scale points higher** (4.40 lower to 6.73 higher)	‐	67 (1 RCT)	⊕⊕⊝⊝ **Low^2^**	‐
**School achievement: Additional educational support needs**	‐	‐	‐	(0 studies)	‐	No data available
**Cognitive function: Composite executive function**	‐	‐	‐	(0 studies)	‐	No data available
**Cognitive function: Inhibition control**	‐	‐	‐	(0 studies)	‐	No data available
**Adverse events**	‐	‐	‐	(0 studies)	‐	No data available
*The effect sizes are differences in standard deviations. To facilitate interpretation we have used rules of thumb in interpretation of effect size (section 12.6.2 in Higgins 2011), where a standard deviation of 0.2 represents a small difference between groups, 0.5 represents a moderate difference, and 0.8 represents a large difference. ** Different assessment tools were used to assess school and cognitive outcomes. We therefore calculated standardised mean differences to assess the effect size between intervention and control groups. **SMD:** Standardised mean difference; **MD:** mean difference; **CI:** Confidence interval						
**GRADE Working Group grades of evidence** **High quality:** We are very confident that the true effect lies close to that of the estimate of the effect **Moderate quality:** We are moderately confident in the effect estimate: The true effect is likely to be close to the estimate of the effect, but there is a possibility that it is substantially different **Low quality:** Our confidence in the effect estimate is limited: The true effect may be substantially different from the estimate of the effect **Very low quality:** We have very little confidence in the effect estimate: The true effect is likely to be substantially different from the estimate of effect						

^1^Downgraded two levels due to high risk of detection and attrition bias. ^2^Downgraded two levels due to high risk of detection bias and imprecision due to a low sample size.

## Background

### Description of the condition

Overweight and obesity are conditions of excessive body fat accumulation. In clinical practice, child and adolescent overweight and obesity are commonly identified by age‐ and gender‐specific body mass index (BMI) percentiles, BMI standard deviation scores, and waist circumference (WC) percentiles relative to a reference population (Reilly 2010; Rolland‐Cachera 2011).

The primary criteria used to define overweight and obesity include:

1. overweight: BMI or WC ≥ 85th percentile to 95th percentile, BMI > one standard deviation above the average;
2. obesity: BMI or WC > 95th percentile, BMI > two standard deviations above the average.

Also, BMI cut‐offs from the International Obesity Task Force (IOTF) are often used as a definition of overweight and obesity. These age‐specific BMI cut‐offs were constructed to match the definition for overweight and obesity in adults (BMI ≥ 25 kg/m^2^ and BMI ≥ 30 kg/m^2^, respectively) (Cole 2000). Recently, the IOTF BMI cut‐offs were reformulated to allow BMI to be expressed as standard deviation or percentile (Cole 2012).

A recent analysis of population data of children aged five to 19 years estimated that in 2016 obesity was identified in 50 million girls and 74 million boys worldwide (NCD Risk Factor Collaboration 2017). In the USA in 2014, the prevalence of child and adolescent obesity (BMI > 95^th^ centile) was 9.4% (two to five years), 17.4% (six to 11 years), and 20.6% (12 to 19 years) (Ogden 2016). In Europe, obesity prevalence was on average 4.0% in adolescents, with vast differences between countries (Inchley 2017). For example, in Scotland the prevalence was 15% in adolescents aged 12 to 15 years (SHeS 2016). Childhood obesity prevalence is increasing in middle‐ and low‐income countries (NCD Risk Factor Collaboration 2017), for example, up to 40% of children in Mexico were living with obesity or overweight, 32% in Lebanon and 28% in Argentina (Gupta 2012).

Health problems are common in children and adolescents with obesity. These include cardiovascular conditions (e.g. hyperlipidaemia, hypertension), endocrinologic conditions (e.g. Type 2 diabetes, metabolic syndrome), gastrointestinal conditions (non‐alcoholic fatty liver disease), respiratory conditions (e.g. obstructive sleep apnoea), musculoskeletal disorders, (e.g. slipped capital femoral epiphysis) and psychosocial disorders (e.g. depression, anxiety) (Grant‐Guimaraes 2016; Han 2010; Puder 2010; Puhl 2007; Su 2015).

Cognitive deficits in children and adolescents (Bruce 2011; Delgado‐Rico 2012a; Liang 2013; Martin 2016; Yu 2010) and academic deficits in adolescents associated with obesity have been observed (Booth 2014; Martin 2017). Cognitive skills such as the ability to suspend prepotent or default responses (inhibition), to switch between rules and responses (cognitive flexibility), to keep and retrieve information while working on a new task (working memory), and to concentrate (attention) are understood to predict school achievement in children and adolescents (Jacob 2015). Collectively, these cognitive abilities are known as executive functions. Evidence from prospective cohort studies suggests that obesity‐related deficits in school achievement are more prevalent in adolescent girls than in boys and younger children (Martin 2017).

The academic consequences of adolescent obesity are shown to persist beyond schooling negatively influencing socioeconomic success. A Finnish longitudinal study (N = 9754, follow‐up 17 years) suggests that adolescent obesity predicts unemployment in later life, with educational achievement as a mediating factor (Laitinen 2002). A British birth cohort study (N = 12,537) indicates that adolescent obesity (at age 16 years) is associated with fewer years of schooling and predicts lower income in young women (at age 23 years), including those who are no longer obese (Sargent 1994). These findings were further confirmed by Han 2011, using the National Longitudinal Survey of Youth 1979 (N = 1974, follow‐up 12 to 16 years), and by Sabia 2012, using the National Longitudinal Study of Adolescent Health (N = 12,445, follow‐up 13 years) in the USA. Findings from the National Longitudinal Survey of Youth 1997 in the USA (N = 8427, follow‐up eight years) suggest that obese adolescents had a 39% lower chance of obtaining a college degree than peers of normal weight (Fowler‐Brown 2010). All of these studies accounted for a variety of confounding variables, including measures of socioeconomic status (e.g. parental education, household income).

### Description of the intervention

Clinical guidelines for prevention and treatment of childhood obesity from countries such as the UK (NICE 2013; SIGN 2010), Australia (NHMRC 2003), Canada (Lau 2007) and Malaysia (Ismail 2004) recommend a multicomponent approach that combines:

1. reduced energy intake;
2. increased physical activity (≥ 60 minutes a day, moderate‐to‐vigorous intensity);
3. decreased sedentary behaviour (e.g. screen time less than two hours a day);
4. cognitive‐behavioural techniques (e.g. goal setting, self‐monitoring, self‐regulation).

The recently updated series of Cochrane Reviews on the treatment of childhood and adolescent obesity concluded that interventions aiming to alter eating habits, physical activity, and sedentary behaviour patterns in a family‐based setting were effective in achieving clinically meaningful weight reduction in children and adolescents (Al‐Khudairy 2017; Colquitt 2016; Mead 2017).

### How the intervention might work

Obesity prevention and treatment interventions could benefit cognition, school achievement and future success of children and adolescents with obesity or overweight differently compared to children and adolescents with a healthy weight. The mechanisms relate to brain development, health and psychosocial consequences, cognitive‐behavioural regulation and lifestyle concerns associated with obesity (Figure 1).

**1:**
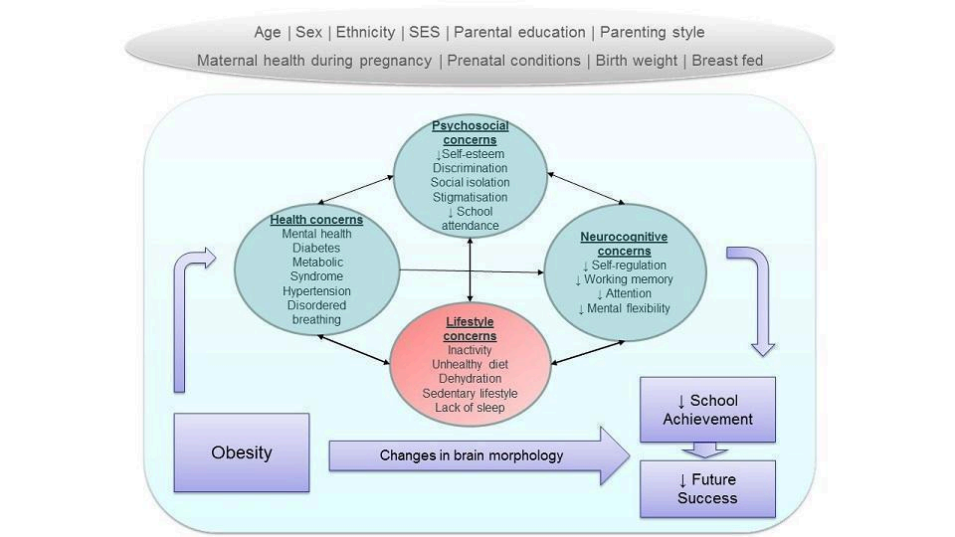
**Potential causal links between obesity and impaired cognitive function, school achievement and future success.** Reverse causation may also occur when cognitive function, school achievement and future success can impact the 'mediating factors', and both in turn may cause worsening of obesity.

#### Brain development

Emerging evidence has linked obesity in children and adolescents to lower brain grey and white matter volume in brain regions associated with cognitive control and learning when compared to children and adolescents with healthy weight (Alarcón 2016; Alosco 2014; Kennedy 2016; Maayan 2011; Ou 2015; Yau 2014). This suggests a direct association between obesity and reduced cognitive and academic abilities, and is consistent with findings from animal models where manipulation of fat mass has been shown to affect cognition, probably as a result of inflammatory mechanisms.

#### Health and psychosocial consequences

Research has also identified obesity‐related health consequences and psychosocial concerns to be associated with lower school achievement and cognitive function. These potential indirect factors include poor sleep due to obesity‐related disordered breathing (Galland 2015; Tan 2014); hypertension (Lande 2015); Type 2 diabetes (Rofey 2015); metabolic syndrome (Yau 2012); decreased school attendance due to adverse physical and mental health (Pan 2013); and social isolation and bullying (Gunnarsdottir 2012a; Krukowski 2009). Reducing the risk of these health and psychosocial concerns, through reduction of obesity or increasing physical activity levels, or both, and improving diet and other obesity‐related behaviours, could have beneficial effects on cognitive function, school achievement and future success in children and adolescents with obesity.

#### Cognitive‐behavioural regulation

The association between lifestyle interventions for weight management and cognition and school achievement might be bidirectional. Research indicates that children with obesity show higher impulsivity and inattention and lower reward sensitivity, self‐regulation and cognitive flexibility compared with their healthy‐weight peers. These neurocognitive correlates were associated with uncontrolled food intake and physical activity behaviour, and thus are assumed to predict weight gain (Francis 2009; Hall 2014; Kulendran 2014; Levitan 2015; Nederkoorn 2006; Smith 2011) or reduction of weight status after an obesity treatment intervention (Naar‐King 2016; Nederkoorn 2007). Lifestyle interventions for weight management might positively impact the neurocognitive factors required for control of food intake. A randomised controlled trial conducted in 44 children (eight to 14 years of age) with obesity or overweight suggested that specific training of self‐regulatory abilities improved weight‐loss maintenance after an inpatient weight‐loss programme in the intervention group compared with the control group (Verbeken 2013). Findings from another randomised controlled overweight treatment programme involving 62 children (mean age 10.3 ± 1.1 years) showed improved problem‐solving skills after an intervention duration of six months (Epstein 2000). Inhibition control skills were improved in 42 obese adolescents from 12 to 17 years of age after 12 weeks of cognitive‐behavioural therapy (Delgado‐Rico 2012b).

#### Lifestyle interventions

Growing evidence has shown that the influence of lifestyle interventions, particularly physical activity and dietary intervention, lie beyond the alteration of energy balance. Many aspects of physical activity, diet and other behaviours have been demonstrated to benefit cognition and school achievement in children and adolescents, regardless of their body weight status, as summarised below.

##### Physical activity

Recently, Faught 2017 reported that meeting the Canadian recommendations for diet, physical activity, sedentary behaviour and sleep at age 11 years was associated with favourable school achievement at age 12 (N = 4253). Low levels of physical fitness (Chaddock 2011; Davis 2011a; Raine 2013) and moderate‐to‐vigorous intensity physical activity have also been linked to impaired cognitive functions in children (Haapala 2017). In addition to the observational evidence, a substantial body of literature suggests a causal relationship between increased levels of physical activity and cognitive function or school achievement or both. For example, a meta‐analysis of 44 experimental and cross‐sectional studies (in participants aged four to 18 years) indicates that increased physical activity caused significant overall improvement in cognitive function and school performance (Hedge's *g* = 0.32; standard deviation (SD) 0.27) (Sibley 2003). A recent meta‐analysis of 21 experimental and quasi‐experimental studies in children aged four to 16 years (N = 4044) also reported a moderate positive effect of physical activity interventions on cognitive outcomes (Hedge's *g* = 0.46, 95% confidence interval 0.28 to 0.64) (Vazou 2016).

Physical activity may affect cognitive function and school achievement through physiological mechanisms (elevated blood circulation, increased levels of neurotrophins and neurotransmitters) (Dishman 2006), learning and motor developmental mechanisms (Pesce 2016a).

##### Dietary modification

Composition of the diet may impact cognition and school achievement by altering neurotrophic and neuroendocrine factors involved in learning and memory. As shown in animal research, these factors are decreased by high‐energy diets containing saturated fat and simple sugars, and are increased by diets that are rich in omega‐3 polyunsaturated fatty acids and micronutrients (Gomez‐Pinilla 2008; Kanoski 2011). These findings were also observed in children. Cross‐sectional data of school‐aged children linked dietary intake of omega‐3 fatty acids to increased memory performance (Baym 2014; Boucher 2011), while consumption of food rich in saturated fatty acids and refined sugar was associated with decreased memory performance (Baym 2014). Longitudinal observational data suggest that diets high in fat and sugar in preschool children (N = 3966; aged three to four years) are associated with decreased intelligence and school performance at primary/elementary school age (Feinstein 2008; Northstone 2011). A controlled healthy school meal intervention over three years in more than 80,000 children led to improved mathematics, English and science achievement (Belot 2011). Promotion of healthier school food at lunchtime and changes in the school dining environment over 12 weeks improved classroom on‐task behaviour in preschool children compared to controls (Golley 2010; Storey 2011). An improvement in dietary quality could therefore have beneficial effects on cognition and school achievement even without improved weight status.

##### Sedentary behaviour

A sedentary lifestyle in children, particularly television‐viewing for two or more hours a day, is associated with the development of obesity or overweight (review of 71 studies; Rey‐Lopez 2008) and may replace opportunities to engage in activities that promote scholastic and cognitive development. To our knowledge, there is no published literature on the effect of reduced sedentary behaviour and improved cognitive and academic outcomes of children and adolescents. However, epidemiological evidence suggests that high levels of sedentary behaviour are associated with reduced school achievement or cognitive abilities. For example, longitudinal data indicate that children younger than three years of age with low television exposure (less than three hours a day) performed better than those with high television exposure (three or more hours a day) in reading (N = 1031) and mathematics (N = 1797) (Peabody Individual Achievement Test) when at preschool age (Zimmerman 2005). Similarly, parent‐reported television viewing in preschool children was inversely related to mathematics achievement at age 10 years (N = 1314) (Pagani 2010) and reading achievement at age 10 to 12 years (N = 308) (Ennemoser 2007). Low TV exposure was also linked to improved school achievement in 8061 adolescents aged 16 years (Kantomaa 2016). Longer‐term educational outcomes may also be affected. Hancox 2005 found that young people (N = 980; follow‐up 21 years) with the highest television viewing time during childhood and adolescence tended to have no formal educational qualifications, and those with a university degree watched the least television during childhood and adolescence. Television viewing for three or more hours a day at age 14 years (N = 678) was associated with a two‐fold risk of failing to obtain a post–secondary/high school education at 33 years of age compared with those watching television for less than one hour a day, mediated by attention difficulties, frequent failure to complete homework and negative attitudes about school at 16 years of age (Johnson 2007). Studies relating accelerometer‐measured sedentary behaviour to cognitive function or school achievement or both indicated that high levels of sedentary behaviour at age seven years were associated with reduced verbal reasoning skills at age 11 (Aggio 2016), and that low levels of sedentary behaviour were associated with increased school achievement at age 10 to 11 years (Aadland 2017).

Reducing sedentary behaviour (TV and screen time, sitting time) might therefore improve cognitive function and school achievement in children and adolescents with obesity or overweight.

##### Multicomponent interventions

In this review, the term 'multicomponent interventions' refers to interventions that target at least two obesity‐related behaviours. Multicomponent lifestyle interventions may benefit cognitive function and school achievement in the general population, i.e. a study population that includes both children and adolescents of normal weight and those with obesity or overweight. For example, after the implementation of an uncontrolled intervention involving healthy nutrition, physical activity and using behaviour change techniques in a US primary/elementary school, an upward trend in reading performance scores was noted; these scores exceeded the national average by 10% after eight years (Nansel 2009). Another uncontrolled experimental study, which implemented a healthy diet and physical activity programme in a primary/elementary school, reported an increase in the numbers of children passing standardised tests in writing, reading and mathematics by 25%, 27% and 31%, respectively (Sibley 2008). A similar but controlled school‐based intervention promoting healthy eating and physical activity behaviour in children aged 11 to 14 years led to significant improvement in mathematics, listening and speaking scores after only five weeks compared with the control condition (standard classroom education) (Shilts 2009).

### Why it is important to do this review

The current global trend in childhood obesity (NCD Risk Factor Collaboration 2017; WHO 2016) suggests that the prevalence of cognitive and educational problems among children is also likely to increase. Given the evidence of a link between low school achievement and economic disadvantage, this might have financial repercussions for future employability and income.

The beneficial effects of changes in diet, physical activity, sedentary behaviour and thinking patterns for prevention and treatment of childhood obesity are well established (Al‐Khudairy 2017; Colquitt 2016; Mead 2017; Waters 2011) and are reflected in clinical guidelines for the management of obesity (Ismail 2004; Lau 2007; NHMRC 2003; NICE 2013; SIGN 2010).

Animal models and human studies suggest that both obesity and obesity‐related lifestyle behaviours have the potential to impair cognitive function, learning, and school achievement (see How the intervention might work; Figure 1). What is less clear is the extent to which interventions which modify lifestyle or body fatness or both can improve cognitive function and learning/school achievement. We would expect that obesity prevention or treatment interventions benefit children with obesity differently from children with a healthy weight by mitigating cognitive deficits which are associated with having an excessive level of body fatness.

The first version of this review was published in March 2014 and included analysis of six trials published until May 2013 (Martin 2014). An update of the review was required to reflect the growing interest in this field.

## Objectives

To assess whether lifestyle interventions (in the areas of diet, physical activity, sedentary behaviour and behavioural therapy) improve school achievement, cognitive function (e.g. executive functions) and/or future success in children and adolescents with obesity or overweight, compared with standard care, waiting‐list control, no treatment, or an attention placebo control group.

## Methods

### Criteria for considering studies for this review

#### Types of studies

Randomised controlled trials (RCTs), including cluster‐randomised trials, and quasi‐randomised trials with or without cross‐over design, were eligible for inclusion. We included cross‐over trials when data from the first period were obtainable.

#### Types of participants

Children and adolescents with obesity or overweight aged three to 18 years attending preschool or school, and whose body weight status was determined using age‐ and gender‐specific BMI percentiles, BMI z‐scores, BMI standard deviation scores (SDSs), BMI cut‐off points or waist circumference. Classification of weight status needed to be based on a relevant national or international reference population for inclusion.

We did not exclude studies on the basis of location.

We excluded children with medical conditions known to affect weight status and academic achievement, such as Prader‐Willi syndrome and diagnosed intellectual disabilities.

#### Types of interventions

Studies were eligible for inclusion when the interventions aimed to prevent or reduce obesity. For inclusion, interventions had to be lifestyle interventions of any frequency and duration provided in any setting (e.g. clinics, schools, community centres) that comprised one or more of the following.

1. Interventions to increase physical activity
2. Dietary and nutritional interventions (excluding supplements)
3. Interventions to decrease sedentary behaviour, screen time and TV time
4. Psychological interventions to facilitate weight management

Interventions could target children or adolescents with or without the participation of family members.

We excluded studies which implemented a physical activity programme aiming to improve cognitive and academic outcomes without a stated intention to prevent or treat childhood obesity. Where any measure or proxy of adiposity was included as a covariate only, the study was not eligible for inclusion. We excluded pharmacological and surgical interventions because these are likely to be conducted in a less representative sample, thus limiting generalisability.

Eligible control interventions were waiting list, attention placebo control, no treatment, and standard practice.

#### Types of outcome measures

Primary and secondary outcomes did not serve as criteria for selection of studies based on title and abstract. Assessment of particular outcome measures was a criterion for inclusion in this review when we screened full texts. We restricted the review to particular outcomes because the same interventions were studied in the same populations for different purposes, for example change in BMI, BMI z‐scores, weight, health‐related quality of life, all‐cause mortality, morbidity, behaviour change (Al‐Khudairy 2017; Colquitt 2016; Mead 2017).

We extracted outcome data at the end of the intervention and at any other follow‐up time point.

##### Primary outcomes

1. School achievement (Morris 2011), recorded by appropriately‐trained investigators (e.g. teachers, researchers). We excluded participant‐ and parent‐reported data.  
  - Average achievement of subjects taught at school.
    - Average across subjects taught at school over one academic year, for example, grade point average (GPA).
  - Achievement in a single subject taught at school.
    - Scores of subjects taught at school or standard achievement test scores for (a) mathematics, (b) reading or (c) language.
    - Validated tests for school achievement in mathematics, reading or language, for example, Woodcock‐Johnson Tests of Achievement III (McGrew 2011).
  - Special education classes.
    - Need for special education class.
    - Reduction of time allocated for special education class.
2. Cognitive function (Carroll 1993): measures of general cognitive ability or different cognitive domains (e.g. composite executive function, inhibition control, attention, memory) assessed using validated cognitive tests administered by appropriately‐trained investigators, such as qualified psychologists. We excluded participant‐reported and parent‐reported data.
3. Adverse outcomes: include, but are not limited to, reduced school attendance, musculoskeletal issues (e.g. activity‐related injury), and psychological issues (e.g. bullying, stigmatisation, depression, eating disorders) obtained from school records, medical records and self‐reports (for bullying and stigmatising events only). We included studies reporting adverse events only when measures of school achievement, cognitive function and/or future success were also reported.

##### Secondary outcomes

1. Future success: includes, but is not limited to, total years of schooling, high school completion, enrolment in higher education, rates of full‐time employment, monthly earnings, home ownership, no/reduced need of social services, obtained from administrative records and self‐reports.
2. Obesity indices: age‐ and gender‐specific BMI, BMI z‐scores and BMI‐SDSs when obtained from measured (not self‐reported) weight and height, measured waist circumference and measures of body fatness by dual‐energy x‐ray absorptiometry (DXA) and bioelectrical impedance analysis (BIA). We included studies reporting obesity indices only when measures of school achievement, cognitive function and/or future success were also reported. Inclusion of these data might enable the review authors to examine whether any changes in school performance, cognitive function and/or future success variables occur independently from changes in obesity (see How the intervention might work). It was not our intention to assess the effect of interventions for treatment of childhood obesity on adiposity or body weight status. This has recently been examined in three other Cochrane Reviews (Al‐Khudairy 2017; Colquitt 2016; Mead 2017).

### Search methods for identification of studies

#### Electronic searches

We previously ran searches in 2012 and 2013. For this update, we searched 17 databases and two trials registers listed below in February 2017. Out of the 17 databases, 12 were searched by the Information Specialist of the Cochrane Developmental Psychosocial and Learning Problem Group. The first review author searched the remaining databases and the trials registers.

1. Cochrane Central Register of Controlled Trials (CENTRAL; 2017, Issue 1) in the Cochrane Library, which includes the Cochrane Developmental, Psychosocial and Learning Problems Specialised Register (searched 2 February 2017).
2. Ovid MEDLINE (1946 to January Week 4 2017).
3. Ovid MEDLINE E‐PUB (searched 2 February 2017).
4. Ovid MEDLINE In‐P (searched 2 February 2017).
5. Embase Ovid (1974 to 2017 Week 05).
6. PsycINFO Ovid (1806 to January Week 5 2017).
7. CINAHL Plus EBSCOhost (Cumulative Index to Nursing and Allied Health Literature; 1937 to 3 February 2017).
8. ERIC EBSCOhost (Education Resources Information Center; 1966 to 3 February 2017).
9. SPORTDiscus EBSCOhost (1980 to 6 February 2017).
10. IBSS ProQuest (International Bibliography of Social Science; 1951 to 3 February 2017).
11. Conference Proceedings Citation Indexes (CPCI; 1990 to 2 February 2017).
12. *Cochrane Database of Systematic Reviews* (CDSR; 2017, Issue 2) part of the Cochrane Library (searched 2 February 2017)
13. Database of Reviews of Effectiveness (DARE; 2015, Issue 2) part of the Cochrane Library (searched 3 February 2017).
14. Database of Promoting Health Effectiveness Reviews (DoPHER; eppi.ioe.ac.uk/webdatabases4/Intro.aspx?ID=9; searched 6 February 2017).
15. EPPI‐Centre Database of Health Promotion Research (Bibliomap; eppi.ioe.ac.uk/webdatabases/Intro.aspx?ID=7; searched 6 February 2017).
16. Trials Register of Promoting Health Interventions (TRoPHI; eppi.ioe.ac.uk/webdatabases4/Intro.aspx?ID=12; searched 6 February 2017).
17. Dissertations and Theses Global ‐ ProQuest (searched 8 February 2017)
18. ISRCTN Registry (www.isrctn.com; searched 8 February 2017 )
19. WHO International Clinical Trials Registry Platform (WHO ICTRP: who.int/trialsearch; searched 8 February 2017).

Search strategies are reported in Appendix 1.

#### Searching other resources

We searched for eligible studies in the reference lists of included studies and in relevant reviews and guidelines.

We handsearched volumes 1 to 10 of The Journal of Human Capital, which is not included in the Cochrane Collaboration's Master List of Journals Being Searched (us.cochrane.org/master‐list) and is not comprehensively indexed by the databases we searched.

We contacted authors of included studies when outcome data were missing or when we required further details on methodology.

When necessary, we translated the title and abstract of non–English language studies. If the study appeared to be eligible for inclusion, we obtained the full article and a translation of the article for further assessment. We obtained translations for articles written in Chinese (Mandarin), Korean, Spanish, Turkish, Portuguese, and Persian.

### Data collection and analysis

#### Selection of studies

We used the web‐based software platform Covidence to view, screen and select studies. AM, JNB and YL independently screened titles and abstracts and assessed their eligibility to identify potentially relevant trials. AM, YL and DHS assessed full reports for eligibility. We resolved different opinions about eligibility by discussion; when the review authors did not agree, the other review authors (JS and JJR) arbitrated. We recorded the reasons for excluding trials in the PRISMA diagram.

#### Data extraction and management

AM, YL and DHS extracted study characteristics using a predefined data extraction form, with AM and YL cross‐checking the extracts. The data extraction form included the following items:

General information: review author ID, title, published or unpublished, study authors, year of publication, country, contact address, source of study.

Methods (including 'Risk of bias' assessment): study design, randomisation methods, allocation concealment, blinding, handling of missing data, selective data reporting.

Population: age, gender, ethnicity, proportion of children with obesity or overweight; inclusion and exclusion criteria; number of participants recruited, included and followed (total and in comparison groups); diagnostic criteria of overweight or obesity; comparability of groups at baseline; comorbidities.

Intervention: type(s), frequency, mode of delivery, intensity of physical activity, methods and timing of comparison of intervention, setting, intervention and follow‐up duration, who delivered the intervention, attrition rates, assessment of compliance, details of comparison and control.

Outcome: assessor characteristics, baseline measures, measures immediately after intervention and at follow‐up, follow‐up time points, validity of measurement tools, definition of outcome (e.g. units, scales), primary outcomes, secondary outcomes.

Results: Where no suitable published data were available, AM contacted the study authors to obtain unpublished data for children and adolescents with overweight or obesity, which were a subgroup of the study sample. AM therefore extracted the result data for each outcome (mean, events, measures of variance, sample sizes), which were double‐checked by YL.

#### Assessment of risk of bias in included studies

AM and DHS independently assessed the risks of bias in each trial, using the Cochrane 'Risk of bias' tool (Chapter 8.5 in Higgins 2011). Findings were cross‐checked and discrepancies resolved through discussion. This included assessment of selection bias (random sequence allocation and allocation concealment), performance bias (blinding of participants and personnel), detection bias (blinding of outcome assessment), attrition bias (incomplete outcome data), reporting bias (selective reporting) and other sources of bias. The review authors judged the risk of bias as 'high', 'low' or 'unclear', using the information provided.

#### Measures of treatment effect

We calculated or extracted the mean change from baseline for intervention and comparison groups, and calculated the mean difference (MD) of change between the groups, when continuous data (e.g. numerical marks) were measured on the same scale. When similar outcomes were measured on different scales, we calculated the standardised mean difference (SMD). Where it was not possible to determine the change from baseline, we calculated MD or SMD using post‐intervention (endpoint) values.

There is no consensus regarding the most appropriate method to use in assessing cognitive ability and school achievement; different researchers tend to use different tools to measure the same outcome. Where the same outcome was assessed across different intervention types, we reported SMD for findings from single‐study and multiple‐study analyses to allow the comparison of intervention effects across intervention types. To ease interpretation of the effect size, we also reported the MD of effect sizes for single‐study outcomes.

We calculated all effect sizes so that positive effect sizes indicate better performance on cognitive function and school achievement outcomes in favour of the intervention group compared to the comparison group.

Included studies did not provide dichotomous or ordinal data. However, in Appendix 2, we describe how we intend to treat these types of data if available, as predefined in our protocol (Martin 2012).

#### Unit of analysis issues

##### Cluster‐randomised trials

We scanned all included studies with clustered randomisation of participants for appropriate analysis of clustered data. Ignoring the proportion of total variance attributable to clustering can result in underpowered study designs and inflation of type I error rates, i.e. increased false‐positive results (Brown 2015). Therefore, for studies in which control of clustering was missing or insufficient at sample size calculation or analysis stage, and when individual participant data were not available, we approximately corrected the intervention effects of cluster‐RCTs. We reduced the size of each trial to its 'effective sample size' (Higgins 2011). We calculated the effective sample size in studies with continuous data by dividing the sample size by the design effect, which is [1 + (M‐1)* ICC], where M is the average cluster size and ICC is the intracluster correlation coefficient. When no ICC was obtainable, we used the ICC estimate of a similar study. In Appendix 3, we provide an overview of the ICCs used to estimate the effective sample size. Some trial authors provided recalculated ICCs for school or cognitive outcomes, or both, which were previously unpublished. We performed a sensitivity analysis to determine the robustness of conclusions from meta‐analyses that included cluster‐randomised trials (see Sensitivity analysis).

##### Cross‐over trials

We considered cross‐over trials as eligible for inclusion if participants were randomly assigned into the first period. We included only data from the first period before the cross‐over took place.

##### Multiple interventions per individual

We performed separate comparisons for studies that compared the effects of a single intervention (e.g. physical activity alone) versus a control condition and studies that compared a combination of any types and numbers of interventions of interest (e.g. physical activity with health behaviour education) versus a control condition.

We entered multiple intervention arms of the same study as separate interventions in the meta‐analysis. We divided the sample size of the control group by the number of intervention arms in the study to avoid overestimating the pooled effect size. We left the means and standard deviations unchanged, as recommended in the *Cochrane Handbook for Systematic Reviews of Interventions* (Section 16.5.4. Higgins 2011).

##### Multiple time points

In separate meta‐analyses, we analysed data from studies that reported results at more than one time point with comparable data of other studies at similar time points.

#### Dealing with missing data

When possible, we recorded characteristics of, reasons for and quantities of missing data for all included studies. We contacted trial authors to obtain information on missing data, if not reported. In our analyses, we ignored data judged to be 'missing at random'. When possible, we imputed missing values in individual participant data, using the last observation carried forward (LOCF) method. We performed sensitivity analyses to examine the effects of including imputed data in meta‐analyses (see Sensitivity analysis).

Included studies did not provide sufficient individual participant data to perform an individual participant data meta‐analysis. Should these become available from the study authors and prove to benefit the review, we will follow the guidance in Higgins 2011 (Chapter 18).

#### Assessment of heterogeneity

We assessed clinical heterogeneity by comparing the similarities of included studies in terms of participants, interventions (type, duration, mode of delivery, setting) and outcomes. By comparing study design and risks of bias, we evaluated methodological heterogeneity. We assessed statistical heterogeneity across studies by visual inspection of the forest plot, and we used the Chi^2^ test with a significance level of P < 0.1 because of its low power in detecting heterogeneity when studies are low in sample size and numbers of events (section 9.5.2 Higgins 2011). Guided by the *Cochrane Handbook* (section 9.5.4 Higgins 2011), we estimated the between‐study variance in a random‐effects meta‐analysis (Tau^2^) in addition to the percentage of variability of intervention effect due to statistical heterogeneity ( I^2^ ). Variability greater than 50% may indicate moderate to substantial heterogeneity of intervention effects (section 9.5.2 Higgins 2011). Furthermore, we assessed the cause of heterogeneity by conducting subgroup and sensitivity analyses, as described below (see Subgroup analysis and investigation of heterogeneity; Sensitivity analysis, respectively).

#### Assessment of reporting biases

We had planned to assess reporting bias by using funnel plots but were unable to do so because of insufficient numbers of included studies (see Appendix 2 and Martin 2012).

#### Data synthesis

We used Review Manager 5 (RevMan 5) (Review Manager 2014) for data entry and analysis. We combined outcome data from included studies in meta‐analyses when the outcome measure addressed the same measurement concept (e.g. mathematics achievement). Where separate data for children and adolescents with overweight and for children and adolescents with obesity were available, we included them separately in the meta‐analysis. This was done with the intention to explore a potential ‘dose‐response’ of the intervention effect relative to the weight category. Where the same study reported several outcome variables for one outcome measurement, we included the outcome variable that was comparable with outcomes reported by other included studies. For example, if reaction time and errors were both given for the cognitive outcome 'attention', then we reported only errors to ensure comparability with other studies which solely reported errors.

Health behaviour interventions have inherent heterogeneity due to intervention implementation and setting, so the true intervention effect is likely to vary between studies. We therefore pooled data using the random‐effects model and provided effect sizes of studies that were inappropriate to include in a meta‐analysis.

##### 'Summary of findings' tables

We summarised outcomes relevant for decision‐making in health and education practice or policy or both (Balshem 2011) in 'Summary of findings' tables, using the GRADE approach. The recommended number of primary outcomes to be reported in the table is seven. We considered the following outcomes to be the most relevant:

1. Average achievement across subjects taught at school;
2. Mathematics achievement;
3. Reading achievement;
4. Additional educational support needs;
5. Composite executive functions;
6. Inhibition control;
7. Adverse events.

We used the GRADEprofiler Guideline Development Tool (GRADEpro GDT 2015) to generate the tables for which we imported data directly from RevMan 5 (Review Manager 2014). These comparison‐specific tables provide details for each outcome concerning the assessment tools used, follow‐up range, timing of follow‐up, study design, number of studies, total sample sizes, effect estimates, and the quality of evidence. Two review authors (AM, DHS) assessed the quality of the evidence, resolving disagreements through discussion with a third review author (JNB).

We determined the quality of the evidence by assessing the methodological quality on outcome level, heterogeneity, the directness of evidence, the precision of evidence, and risk of publication bias. Where the evidence came from small studies, we assessed the extent of the limitation of 'unclear risk of bias on randomisation' on our confidence in the evidence by consulting the risk‐of‐bias item ‘comparability of groups at baseline’. We did not consider an unclear risk of selection bias as a serious limitation where we had rated the risk‐of‐bias item ‘comparability of groups at baseline’ at low risk of bias. A low risk of bias of known baseline characteristics may suggest adequate randomisation, so we have confidence in the evidence. Where we rated ‘comparability of groups at baseline’ at unclear or high risk of bias, we considered an 'unclear risk of bias on randomisation' as a serious limitation and so downgraded the quality of evidence to reflect our limited confidence in the evidence. However, we acknowledge that variables that were not tested for may cause imbalance between groups and that imbalances can occur by chance, despite adequate randomisation.

GRADE specifies four quality levels:

1. High quality: further research is very unlikely to change our confidence in the effect estimate.
2. Moderate quality: further research is likely to have an important impact on our confidence in the effect estimate and may change the estimate.
3. Low quality: further research is very likely to have an important impact on our confidence in the effect estimate and may change the estimate.
4. Very low quality: we are very uncertain about the effect estimate.

For ease of interpretation of the standardised effect sizes, we applied rules of thumb, where a standard deviation (SD) of 0.2 represents a small difference between groups, 0.5 represents a moderate difference, and 0.8 represents a large difference (section 12.6.2 in Higgins 2011). Where both change‐from‐baseline and endpoint data were available for the same outcome, we reported the evidence of highest quality. When the quality of evidence was the same for outcomes generated from endpoint and change‐from‐baseline data, we reported change‐from‐baseline outcomes in the 'Summary of findings' table.

#### Subgroup analysis and investigation of heterogeneity

Subgroup analyses are principally intended to investigate sources of heterogeneity within a meta‐analysis in relation to factors that potentially impact outcomes. We identified several potentially influential participant and intervention characteristics for subgroup analyses (see Appendix 2). The low number of studies included for the same outcome did not allow us to perform meaningful subgroup analyses for all predefined sources of heterogeneity. However, we performed a subgroup analysis for body weight status (overweight versus obesity), where possible.

#### Sensitivity analysis

We investigated the influence of study characteristics on the robustness of the review results by conducting sensitivity analyses. We removed trials from the analysis when studies:

1. used different criteria or variations in the thresholds of criteria to define childhood obesity and overweight (e.g. clinical versus public health thresholds);
2. were judged at 'high risk of bias' in the characteristics of random sequence allocation, concealment of allocation, blinding and extent of dropouts;
3. were cluster‐RCTs or cross‐over trials;
4. provided a post‐intervention mean and standard deviations but where change‐from‐baseline data were missing.

## Results

### Description of studies

See Characteristics of included studies; Characteristics of excluded studies; Characteristics of studies awaiting classification; Characteristics of ongoing studies.

#### Results of the search

For the original review (Martin 2014), we screened 17,748 titles and abstracts, and excluded 17,219 records. We retrieved 529 full‐text reports, of which we included six studies (14 reports) in the review.

The electronic search for this review update yielded 17,577 records. We found two more records by screening the reference lists of relevant systematic reviews. We also carried forward 17 reports from the previous review that had been classified as ongoing or awaiting classification. Overall, our updated search yielded 17,596 records.

Having excluded 6131 duplicate records, we screened the remaining 11,465 on the basis of title and abstract, and discarded 10,806 as irrelevant.

For 60 records of conference papers, only abstracts were available. We contacted the authors of the conference abstracts for further information and followed up on non‐responders two weeks later. We received eighteen replies. Fifteen study authors stated that their study did not meet our inclusion criteria (Criteria for considering studies for this review), and we excluded these 15 records at title and abstract stage, along with 42 abstracts for which we were unable to make a decision due to insufficient information. Three authors supplied us with the full‐text report of their studies, which we screened and discarded at full‐text stage (see Excluded studies).

We retrieved 599 full‐text reports, of which 12 new studies (36 reports) met our inclusion criteria. We include 18 studies (57 reports) in total in this updated review (see Characteristics of included studies).

Three more studies (four reports) are awaiting classification (see Characteristics of studies awaiting classification). Thirteen trials (14 reports) are currently ongoing (see Ongoing studies). A flow chart of the search results is shown in Figure 2.

**2:**
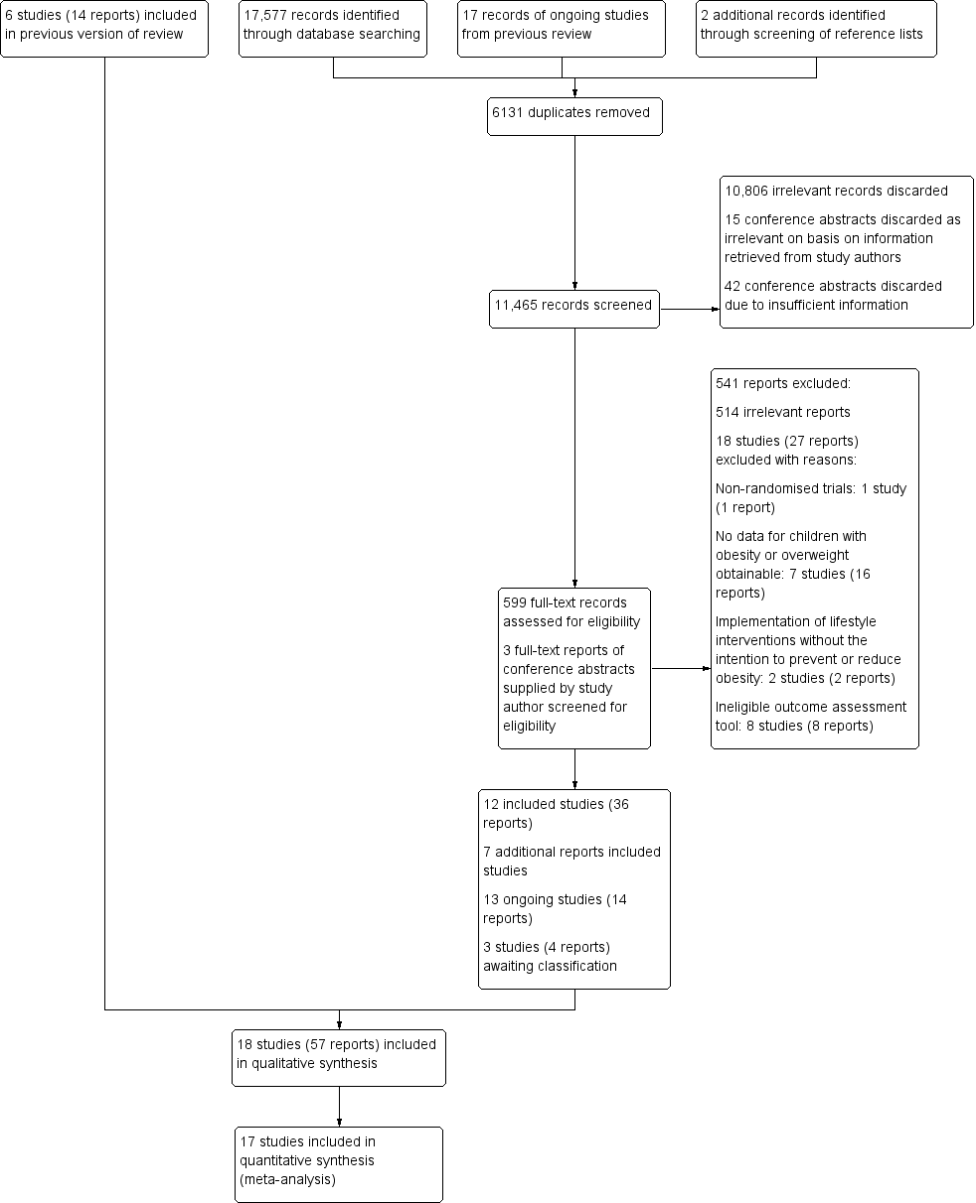
Study flow diagram.

#### Included studies

For 14 of the 18 included studies, outcome data for children and adolescents with obesity or overweight were not published separately from data for the total study population. We therefore contacted the study authors to obtain the unpublished data.

##### Study design and geographical location

We included five RCTs (Chen 2016; Davis 2011b; Huang 2015; Krafft 2014; Staiano 2012) and 13 cluster‐RCTs (Ahamed 2007; Barbosa Filho 2017 [pers comm]; Damsgaard 2017 [pers comm]; De Greeff 2016; Gallotta 2015; Johnston 2013; Melnyk 2013; Nanney 2016; Resaland 2016; Sánchez‐López 2017 [pers comm]; Treu 2017; Winter 2011; Wirt 2013 [pers comm]). Of the 18 studies, eight were conducted in the USA, two in Denmark, and one each in Canada, Brazil, Italy, Spain, Norway, The Netherlands, Germany and Taiwan.

##### Population characteristics

The numbers of participants randomly assigned ranged from 37 to 360, and the number of participants followed and analysed ranged from 28 to 349 (total N = 2384). Attrition rates varied from zero (Gallotta 2015) to 29% (Ahamed 2007; Nanney 2016).

Two studies were carried out in children attending preschool, with age ranges of three to five years (Winter 2011) and four to seven years (Sánchez‐López 2017 [pers comm]). Eleven studies were conducted in primary/elementary school‐aged children (six to 13 years) (Ahamed 2007; Damsgaard 2017 [pers comm]; Davis 2011b; De Greeff 2016; Gallotta 2015; Huang 2015; Johnston 2013; Krafft 2014; Resaland 2016; Treu 2017; Wirt 2013 [pers comm]). One study included adolescents in junior high/secondary school‐aged 12 to 15 years (Chen 2016) and another three studies included adolescents aged 14 to 18 years (Nanney 2016; Melnyk 2013; Staiano 2012). The study population in Barbosa Filho 2017 [pers comm] included adolescents from 11 to 18 years.

The overall proportions of girls with obesity or overweight were 64%, 57% and 53% in Sánchez‐López 2017 [pers comm], Staiano 2012 and Wirt 2013 [pers comm], respectively. These three studies did not report the gender distribution between intervention and comparison groups. There was a roughly equal gender distribution between intervention and comparison groups in four studies only (Barbosa Filho 2017 [pers comm]; Nanney 2016; Resaland 2016; Treu 2017). Five studies had a higher proportion of female participants in the intervention compared to the control group: Ahamed 2007 (48% versus 19%); Damsgaard 2017 [pers comm] (72% versus 59%); Gallotta 2015 (52% versus 36% ); Krafft 2014 (71% versus 58%); and Melnyk 2013 (54% versus 48%). A higher proportion of girls in the control group was evident in six studies: Chen 2016 (36% versus 52%); Davis 2011b (54% versus 62%); De Greeff 2016 (52% versus 69%); Huang 2015 (53% versus 59%); Johnston 2013 (38% versus 46%); and Winter 2011 (25% versus 37%).

Where data were obtainable, ethnic majorities in the study populations were African‐American (Davis 2011b; Krafft 2014; Staiano 2012), Hispanic (Johnston 2013; Melnyk 2013; Winter 2011), Asian (Chen 2016), South European (Sánchez‐López 2017 [pers comm]), South‐East European (Wirt 2013 [pers comm]), and North European (Damsgaard 2017 [pers comm]; Huang 2015; Resaland 2016). In Nanney 2016 and Treu 2017, most participants were of white European ethnic origin.

Of the 18 included studies, four reported that most of their participants were from low‐income families (Barbosa Filho 2017 [pers comm]; Chen 2016; Staiano 2012; Winter 2011).

##### Intervention characteristics

The interventions fell into three categories:

1. Physical activity only (eight studies);
2. Physical activity plus healthy lifestyle education (seven studies);
3. Dietary interventions including nutrition education (three studies).

Table 4 provides an overview of the specific intervention content. For a more detailed description of the interventions see Characteristics of included studies).

**1:** Intervention content of included studies

**STUDY**	**INTERVENTION CONTENT**
**Physical activity only interventions**	
**Chen 2016**	Group physical activity programme including multiple types of moderate‐intensity exercises performed 4 times/week for 40 minutes per session (5 minutes each for warm‐up and cool‐down, 30 minutes for the main exercise). The participants were free to choose one of the provided exercise types (e.g. fast walking, stair climbing, jumping rope, or aerobic dancing), with an emphasis on maintaining a moderate intensity of 60% to 70% of the maximal heart rate. Intervention was offered during the school day in the morning, during lunch break, or after school for 3 months
**Davis 2011b**	Aerobic group exercise for 40 minutes 5 times/week, over a mean total of 13 weeks. Five‐minute warm‐up phase consisted of brisk walking and static and dynamic stretching. Children were encouraged to maintain a heart rate > 150 beats/minute during running games, tag games, jump rope, modified basketball and football. The intervention involved no competition or skill enhancement and was delivered in an after‐school setting
**De Greeff 2016**	Fit en Vaardig op school (Fit and academically proficient at school) involved physically active academic lessons which ran over 44 weeks in total over 2 school years with 3 lessons/week. The lessons had a duration of 20 – 30 minutes, with 10 – 15 minutes spent on solving mathematical problems and 10 – 15 minutes spent on language. During the lessons all children started with performing a basic exercise, such as jogging, hopping in place or marching. A specific exercise was performed when the children solved an academic task. The physical activities were aimed to be of moderate‐to‐vigorous intensity
**Gallotta 2015**	The 2 intervention conditions had the same structure and took place in the school. They included 15 minutes of warm‐up, 30 minutes of moderate‐to‐vigorous physical activities, and 15 minutes of cool‐down and stretching. The traditional physical activity intervention consisted of continuous aerobic circuit training followed by a sub‐maximal shuttle run exercise. This intervention focused on the improvement of cardiovascular endurance by performing different types of gaits (e.g. fast walking, running, skipping) without any specific co‐ordinative request. The co‐ordinative physical activity intervention focused on the development of psychomotor competences and expertise in movement‐based problem‐solving through functional use of a common tool (e.g. basketball), and considering various tasks that involved decision‐making motor tasks and manipulative ball‐handling skills
**Krafft 2014**	See Davis 2011b. The intervention duration was extended to 8 months.
**Sánchez‐López 2017 [pers comm]**	MOVI‐KIDS is a multidimensional intervention that consisted of a standardised extra‐curricular non‐competitive physical activity programme of 4½ hours/week; informative sessions to parents and teachers about how schoolchildren can become more active, and interventions in the playground (environmental changes: equipment, facilities, painting, etc.) aimed to promote physical activity during recess (MOVI‐Playground)
**Staiano 2012**	Nintendo Wii EA Sports Active exergame played in competitive condition individually or in co‐operative condition in pairs for 30 to 60 minutes, 5 days/week, over a period of 10 weeks in total. The fitness video game included cardio activities (e.g. inline skating), sports games (basketball, volleyball, tennis, baseball) and strength training
**Resaland 2016**	The Active Smarter Kids (ASK) programme comprised 3 components: i) physically active lessons for 90 minutes/week, conducted in the playground; physically active educational lessons were delivered in 3 core subjects – Norwegian (30 minutes/ week), mathematics (30 minutes/week) and English (30 minutes/week); ii) physical activity breaks (5 minutes/day) implemented in the classroom during academic lessons; and iii) physical activity homework (10 minutes/day)
**Physical activity plus healthy lifestyle education**	
**Ahamed 2007**	Action Schools! BC was a comprehensive, multicomponent intervention providing tools for schools and teachers to use in promoting physical activity and healthy eating in different settings. These include the school environment (healthy eating posters), scheduled Physical Education, classroom action, family and community (e.g. walking school bus), extracurricular activities (e.g. dance club) and school spirit (e.g. Hike across Canada challenge)
**Barbosa Filho 2017 [pers comm]**	Fortaleça sua Saúde ('Strengthen your health') focused on teachers' training and activities on health in the curriculum (including a specific training to Physical Education teachers), active opportunities in the school environment (availability of spaces and materials for physical activity) and health education (production and exhibition of health material at school, and distributing pamphlets to students and parents)
**Huang 2015**	The day‐camp intervention comprised 2 parts: an intensive six‐week day camp intervention and a subsequent 46‐week family‐based intervention programme (13‐month [52 weeks] in total). Children were engaged in physical activity and sports for at least 3 hours a day, achieving about 90 minutes of moderate‐to‐vigorous physical activity per day measured by accelerometry. After the day camp, one physical activity day was offered as part of the family‐based intervention programme. Healthy lifestyle education topics during the 6‐week day camp included nutrition, physical activity and health, and goal‐setting. The family‐based intervention programme comprised 4 parents‐involved meetings targeting daily physical activity and dietary behaviour. In the day camp, 3 meals and 3 snacks were prepared and served according to the national dietary recommendations with no caloric restrictions
**Melnyk 2013**	COPE (Creating Opportunities for Personal Empowerment) programme was a manualised 15‐session educational and cognitive–behavioural skills‐building programme. Each session of COPE contains 15 – 20 minutes of physical activity (e.g. walking, dancing, kick‐boxing movements), not intended as an exercise training programme, but rather to build beliefs in the participants that they can engage in and sustain some level of physical activity on a regular basis. Pedometers were used throughout the intervention. Participants were asked to increase their step counts by 10% each week, regardless of baseline levels, and to keep track of their daily steps. The COPE Healthy Lifestyles TEEN (Thinking, Emotions, Exercise, Nutrition) Programme was delivered once a week as part of a school health curriculum. Participants received a COPE manual with homework activities for each of the 15 sessions that reinforced the content and skills in the programme: cognitive‐behavioural skill building (e.g. problem‐solving and emotional and behavioural regulation), nutrition (e.g. food groups, portion sizes, food labelling), and physical activity (e.g. ways to increase physical activity and associated benefits)
**Treu 2017**	The standard intervention arm of the ASCEND intervention consisted of the Nutrition Detectives (ND) programme and the ABC for Fitness (ABC) programme. ND was a 90‐minute programme that aimed to convey the link between food choices and health, convince students of the need to become "supermarket spies" to learn the truth about the foods that they eat. ABC for Fitness offered brief bursts of physical activity in the classroom, spread over the school day. Classroom teachers offered 30 daily minutes of activity bursts. The activity bursts were designed to include a brief warm‐up and cool‐down (e.g. stretching or low‐intensity activity) along with one or more core activities of higher intensity (e.g. hopping, running in place, jumping jacks, or dancing to music). The enhanced intervention arm included the ND and ABC programmes plus reinforcements of their messages to participants and their families in the school, home, and a supermarket. Family‐focused kits were send home including pedometers, walking tips to increase daily steps, a family log for recording steps, local walking trail guides, walking maps for local grocery stores, physical activity tips sheet, suggestions for activity bursts, family activity challenge cards, a 3‐ minute sand timer to be used for activity challenges, and a log to record the number of activities and repetitions completed, Nutrition Detectives DVD, a reminder card with the programme’s "five clues" to make healthful food choices, grocery store coupons, and a family "homework assignment" to watch the DVD, review the ND clues together, complete an activity applying the clues to foods in the family kitchen. Family nights were held at a) the local supermarket, with stations set up to teach families about healthful food choices with games, demonstrations, and taste tests; b) schools offering stations throughout the building to try out different kinds of exercises, including Frisbee golf and Zumba, and received information or coupons from local fitness‐related businesses
**Winter 2011**	The Healthy & Ready to Learn intervention involved parents and teachers reading children's books on health‐related themes including nutrition and obesity prevention to the participants. Teachers and parents were trained to increase children's time spent physically active in moderate‐to‐vigorous activity for 60 minutes/day. Activities were play‐based and targeted specific gross motor skills. Physical activity equipment was provided
**Wirt 2013 [pers comm]**	Komm mit in das gesunde Boot (‘Join the healthy boat') comprised healthy lifestyle education of 20 teaching sessions a year focusing on increased physical activity, reduced consumption of sugar‐sweetened beverages and reduced screen time. It included 2 physically‐active breaks per school day of 5 to 7 minutes, and a physical activity task to be performed at home involving parents
**Dietary interventions (including health education)**	
**Damsgaard 2017 [pers comm]**	In the OPUS School Meal intervention children received the New Nordic Diet (NND) containing seasonal, health‐promoting ingredients, for example, berries, root vegetables, whole grains, fish, shellfish, seaweed and rapeseed oil. Children received daily servings of a mid‐morning snack, ad libitum hot lunch meal and afternoon snack (fruit dessert twice/week). The children were encouraged to taste everything and to keep a reasonable plate distribution with vegetables and starchy foods filling most of the plate. Each child spent 3 – 5 school half‐days in the kitchen cooking, presenting, and serving the menu of the day to the other children. The teachers were encouraged to participate in the lunch meals. Class teachers were given a box of teaching materials about the human body, the clinical measurements, and taste sensorics, including background information about NND and suggestions for related educational activities and games
**Johnston 2013**	The whole‐school lifestyle education programme involved curriculum material taught by trained teachers, school meal modification towards nutrient‐dense food and nutrition counselling. Teachers were provided with 50 integrated lessons‐worth of curriculum material aiming to improve healthy diet (increased fruit and vegetable, breakfast, healthy snack, water consumption) and increase physical activity. Teachers were encouraged to teach lifestyle‐integrated lessons once a week, to conduct health‐related activities every 2 weeks and to hold a school‐wide health event once a semester
**Nanney 2016**	The Project breakFAST (Fuelling Academics and Strengthening Teens) aimed to improve students’ school breakfast programme (SBP) participation by implementing a grab‐and‐go‐style cart or breakfast line located outside the cafeteria in a high‐traffic hallway, atrium or common area. School‐wide marketing campaigns were developed by a community partner which worked with a group of students to design the marketing campaign at each school. Positive interactions and social support were created by developing school policies, to allow students to eat breakfast in the hallway. Schools were also encouraged to allow eating breakfast in some classrooms when appropriate

Fifteen studies were set in the classroom or within the school environment or both. Of these, in three studies the intervention also included activities in participants' homes (Resaland 2016; Winter 2011; Wirt 2013 [pers comm]). The intervention by Treu 2017 targeted activities in the school environment, at participants’ home and supermarkets. Davis 2011b and Krafft 2014 delivered the intervention as an after‐school programme at the Georgia Prevention Institute. Huang 2015 offered the intervention in the form of a day camp outside the school setting.

###### Physical activity only interventions

Interventions classified as physical activity‐only interventions comprised four types of physical activity programmes:

1. Group aerobic exercise (Chen 2016; Davis 2011b; Gallotta 2015; Krafft 2014)
2. Group co‐ordination skills exercises (Gallotta 2015)
3. Physically active academic lessons (De Greeff 2016; Resaland 2016)
4. Extracurricular individual or small‐group physical activity (Resaland 2016; Sánchez‐López 2017 [pers comm]; Staiano 2012)

In addition to targeting children and teachers, Sánchez‐López 2017 [pers comm] was the only study which also changed the physical activity environment by implementing improvements to the playground. The intervention durations ranged from 10 weeks (Staiano 2012), three months (Chen 2016; Davis 2011b) and five months (Gallotta 2015) to seven months (Resaland 2016), eight months (Krafft 2014), one school year (Sánchez‐López 2017 [pers comm]), and 18 months (De Greeff 2016).

###### Physical activity intervention combined with healthy lifestyle education

These studies employed complex interventions which included promotion of participants’ physical activity levels and knowledge of health behaviours, mainly healthy eating and physical activity. Three studies provided equipment to facilitate engagement in physical activity (Barbosa Filho 2017 [pers comm]; Melnyk 2013; Treu 2017). The physical activity components of the complex intervention varied between studies, and included short classroom‐based physical activities (Ahamed 2007; Melnyk 2013; Treu 2017), school environment‐based physical activity (Ahamed 2007; Barbosa Filho 2017 [pers comm]; Winter 2011; Wirt 2013 [pers comm]), or daily physical activity during a day camp (Huang 2015). The total intervention duration including the health education component ranged from four months (Barbosa Filho 2017 [pers comm]; Melnyk 2013) and six months (Winter 2011) to one school year (Ahamed 2007; Treu 2017; Wirt 2013 [pers comm]) and 13 months (Huang 2015).

###### Dietary interventions

We classified studies into this category when changes in the food environment were implemented and healthy education components targeted primarily healthy eating knowledge. All studies classified as dietary interventions were conducted in the school setting; two studies in primary/elementary schools (Damsgaard 2017 [pers comm]; Johnston 2013) and one in a high school (Nanney 2016). The studies differed substantially in that, in addition to nutrition education, Nanney 2016 targeted the uptake of school breakfast, Damsgaard 2017 [pers comm] replaced packed lunch with the New Nordic Diet, and Johnston 2013 encouraged school cafeteria staff to increase the availability of nutrient‐dense food, whereby the nutrition education component was the primary focus. Damsgaard 2017 [pers comm] delivered the intervention over a duration of three months, Nanney 2016 over one school year, and Johnston 2013 over two school years.

##### Comparison conditions

Regardless of the intervention type, 15 studies compared the intervention with standard practice, referring to the usual school curriculum, including physical education lessons. Of these, four studies applied a wait‐list control condition offering a similar intervention to the comparison group after completion of the intervention duration (Chen 2016; Nanney 2016; Treu 2017; Wirt 2013 [pers comm]). Three studies compared the intervention with an attention placebo control programme (Huang 2015; Krafft 2014; Melnyk 2013). The attention placebo control condition in Krafft 2014 comprised supervised sedentary activities such as art and board games for the same duration and frequency as the intervention group. In Huang 2015, the comparison group received a two‐hour group physical activity intervention once a week and a single session on healthy lifestyle education for parents. Participants in the comparison condition of Melnyk 2013 received a health education programme which covered different topics from the intervention group and did not involve active promotion of physical activity, as was the case in the intervention group. The comparison condition in Huang 2015 and Melnyk 2013 did not match the intervention condition in terms of the intensity (see Table 4 for details). Despite this, we considered the comparison conditions as attention controls because the participants received an active intervention. Gallotta 2015 did not provide details on the nature of the comparison condition.

##### Primary outcomes

In Appendix 4 we summarise additional information on the outcomes and measurement tools used to assess school achievement and cognitive functions. Data were available for five school achievement outcomes: average achievement across subjects taught at school, mathematics achievement, reading achievement, language achievement, and health class grades. Intervention effects for children and adolescents with obesity or overweight were available for the following cognitive functions: composite executive functions, inhibition control, attention, working memory, visuo‐spatial abilities, cognitive flexibility, non‐verbal memory, and general intelligence.

###### School achievement: Average across subjects taught at school

Three studies provided data for average end‐of‐year school achievement obtained from school records as Grade Point Average (GPA) (Johnston 2013; Nanney 2016) or the Canadian Achievement Test (CAT)‐3 (Ahamed 2007).

###### Individual subject performances

####### Mathematics achievement

Across the three intervention types, seven studies assessed mathematics achievement: Canadian Achievement Test (CAT)‐3 (Ahamed 2007), broad maths scale of the Woodcock‐Johnson Tests of Achievement III (Davis 2011b), standardised national mathematics test (Barbosa Filho 2017 [pers comm]; Damsgaard 2017 [pers comm]; Resaland 2016), numerical quantitative concepts scale of the General Differential Aptitude Battery (Sánchez‐López 2017 [pers comm]), and AIMSweb standardised Mathematics Concepts and Application Test (Treu 2017).

####### Reading achievement

Five studies assessed reading achievement: Canadian Achievement Test (CAT)‐3 (Ahamed 2007), broad reading scale of the Woodcock‐Johnson Tests of Achievement III (Davis 2011b), standardised national reading test (Damsgaard 2017 [pers comm]; Resaland 2016), and AIMSweb standardised Reading Curriculum Based Measurement (Treu 2017).

####### Language achievement

Four studies assessed native language achievement and one study assessed English achievement by Norwegian native speakers using standardised national tests (Resaland 2016). Native language achievement was assessed using the Canadian Achievement Test (CAT)‐3 (Ahamed 2007), analogical and complex verbal order scale of the General Differential Aptitude Battery (Sánchez‐López 2017 [pers comm]), standardised national language tests (Barbosa Filho 2017 [pers comm]), and Peabody Picture Vocabulary Test III (Winter 2011). Although receptive vocabulary skills measured by the Peabody Picture Vocabulary Test are often used as measures of general intelligence, we classified these as school achievement outcomes because the trial authors intended to assess school readiness.

####### Health class achievement

One study provided school achievement outcomes in form of teacher‐assessed health class grades (Melnyk 2013).

###### Special education classes

No study reported intervention effects for additional educational support needs.

###### Cognitive function

####### Composite executive functions

Three studies assessed composite executive functions using the Das‐Naglieri‐Cognitive Assessment System (CAS) (Davis 2011b; Krafft 2014) and the Delis‐Kaplan Executive Function System (Staiano 2012) (see Appendix 4 for further details).

####### Inhibition control

Three studies assessed inhibition control using the Stroop Colour Word Test (De Greeff 2016; Huang 2015) and the Go/No‐go task of the KiTAP Attention test battery for children (Wirt 2013 [pers comm]).

####### Attention

Four studies provided outcome data for participants' attention performance: Attention scale of Das‐Naglieri‐CAS (Davis 2011b), d2‐R test of attention (Gallotta 2015), d2‐ test of attention (Damsgaard 2017 [pers comm]), and sustained attention scale of KiTAP (Wirt 2013 [pers comm]).

####### Working memory

One study assessed working memory using the Digit Span Backward test and Visual Span Backward Test (De Greeff 2016).

####### Visuo‐spatial abilities

Four studies assessed visuo‐spatial abilities in children with obesity or overweight using different scales: Simultaneous processing scale of the Das‐Naglieri‐CAS (Davis 2011b; Krafft 2014), logical puzzle figures test of the General Differential Aptitude Battery (Sánchez‐López 2017 [pers comm]) and copy trial of the Rey Complex Figure Test (Huang 2015).

####### Cognitive flexibility

Two studies assessed cognitive flexibility using the Wisconsin Card Sorting Test (Chen 2016; De Greeff 2016)

####### Non‐verbal memory

Three studies assessed non‐verbal memory using the successive processing scale of the Das‐Naglieri‐CAS (Davis 2011b; Krafft 2014) and the recall trial of the Rey Complex Figure Test (Huang 2015).

####### General intelligence

One study provided outcome measures on general intelligence using the General Differential Aptitude Battery (Sánchez‐López 2017 [pers comm]).

###### Adverse events

Although participants in Chen 2016 were asked to record any adverse events during the intervention, no outcome data were reported. Davis 2011b reported a foot fracture as a consequence of participating in the physical activity intervention. The incident occurred in the low‐intensity intervention arm, which we deemed as ineligible for inclusion in this review (see Characteristics of included studies). We therefore did not consider this adverse event in the evidence synthesis. No other adverse events were reported.

##### Secondary outcomes

###### Future success

None of the included studies assessed measures of future success.

###### Obesity indices

Six studies which reported the intervention effect of school or cognitive outcomes also provided change from baseline BMI z‐scores (Damsgaard 2017 [pers comm]; Davis 2011b; Huang 2015; Johnston 2013; Sánchez‐López 2017 [pers comm]; Treu 2017). Three studies reported change in percentage of total body fat, measured using bioelectric impedance analysis (Chen 2016; Gallotta 2015) and dual energy X‐ray absorptiometry (Huang 2015). Waist circumference measures were reported by one study only (Huang 2015).

##### Follow‐up time points

Sixteen studies reported outcomes immediately after completion of the intervention period or before cross‐over of the experimental conditions (Damsgaard 2017 [pers comm]; Sánchez‐López 2017 [pers comm]). Only two studies provided outcome data for two follow‐up time points.

De Greeff 2016 assessed inhibition control, working memory, and cognitive flexibility at six‐month and 18‐month follow‐ups. The first follow‐up time point relates to an intervention mid‐term assessment and the second represents the immediate post‐intervention follow‐up. Personnel who delivered the intervention changed after mid‐term assessment from specially‐trained primary/elementary school teachers to the regular classroom teacher, who also received training in delivering the intervention.

Huang 2015 assessed inhibition control, non‐verbal memory, visuo‐spatial abilities, and obesity indices immediately after completion of the six‐week intensive day camp versus standard practice/attention control intervention, and 13‐month follow‐up from baseline. In the time period between the day‐camp intervention and the 13‐month follow‐up, participants received a low‐intensity family‐based intervention, which could be considered a maintenance intervention.

#### Excluded studies

For this updated review, we excluded 541 full‐text reports (Figure 2), 514 of which we deemed to be irrelevant. We formally excluded 18 studies (27 reports) for the following reasons:

1. One study was a non‐randomised trial (Halberstadt 2017);
2. Seven studies did not report the disaggregated data for children with obesity or overweight (Donnelly 2009; Donnelly 2013; Gentile 2009; Hillman 2014; Murray 2008; Puder 2011; Reed 2010);
3. Two studies employed lifestyle interventions without the intention to prevent or reduce obesity (Crova 2014; Pesce 2016b);
4. Eight studies used non‐eligible tools to assess school or cognitive outcomes (e.g. self‐reported or parent‐reported questionnaires) (Gee 2014; Goldfield 2012; Muzaffar 2014; Naar‐King 2016; Pentz 2011; Salmoirago‐Blotcher 2015; Smith 2015; Wong 2016).

In total, we excluded 534 full‐text reports, of which we deemed 487 to be irrelevant, and 35 studies (47 reports) were formally excluded. See Characteristics of excluded studies tables for the list of excluded studies and reasons for exclusion from the previous and the present review.

##### Studies awaiting classification

Currently, three studies are awaiting classification. Vetter 2015 is available as a conference abstract only and we were not able to retrieve further details of the study due to non‐response from the authors. We have so far contacted the authors twice. NCT02043626 and NCT02122224 are completed studies identified through a trial register, but the results have not yet been published. Based on the information provided in the trial registers, we are not able to determine the eligibility of the studies, namely, whether data for children with obesity or overweight would be available. See Characteristics of studies awaiting classification for further details.

##### Ongoing studies

We identified 13 ongoing studies (14 reports); for details see Characteristics of ongoing studies.

1. Bau 2016 (Maintain study) is evaluating a group intervention on healthy eating and lifestyle factors as part of a weight loss maintenance programme compared to standard practice on school achievement in children and adolescents aged between 10 and 17 years with a BMI > 99th percentile. This study took place in Germany and the analysis of the results is currently ongoing.
2. Cadenas‐Sanchez 2016 (ActiveBrains project) is taking place in Spain, and compares an exercise intervention with wait‐list control aimed at children with obesity or overweight aged eight to 12 years. Cognitive outcomes are executive functions including inhibition control and memory, whereas school achievement will be assessed for mathematics, language and reading achievement.
3. DRKS00005275 (Ballschool ‐ easy) is being conducted in Germany, and is a four‐arm trial comparing three intervention groups (physical activity, diet, physical activity plus diet) with a no‐treatment control for children aged six to 10 years and a BMI > 90th percentile. Overall intelligence will be assessed as a cognitive outcome measure.
4. ISRCTN12698269 (Run‐a‐mile) is a UK‐based study, evaluating the effect of daily walking or running compared to standard practice on teacher‐assessed school achievement in children aged nine to 12 years. Body weight status is not an inclusion criterion but the study evaluates intervention effects on body fat and so relevant data for this review might be available on completion of the study.
5. NCT01737658 has been conducted in the USA, and compares an exercise intervention with standard practice in adolescents aged 14 to 19 years with a BMI > 99th percentile. The results for intervention effects on changes in cognitive functions (not further specified) are currently in preparation for publication.
6. NCT02873715 (PLAN trial) is taking place in the USA, comparing a family‐based treatment programme plus enhanced usual care with enhanced usual care only in children aged six to 12 with a BMI > 85th percentile. Inhibition control will be the relevant outcome of interest for this review.
7. NCT02972164 is being conducted in children aged nine to 12 with a BMI > 95th percentile in Qatar. The study assesses the effect of a three‐phased weight management programme (weight loss camp/after‐school programme/maintenance) compared to standard school routine on inhibition control.
8. Po'e 2013 (Growing Right Onto Wellness) takes place in the USA, and evaluates a weight management intervention with focus on diet and physical activity consisting of an intensive phase, maintenance and sustainability phase compared to a less intensive educational comparison intervention. Children aged three to five years with a BMI equal to or above the 50th percentile and below the 95th percentile are eligible to take part. Executive functions and general intelligence will be assessed.
9. RBR‐38p23s is being conducted in Brazil, and evaluates the effect of a complex/intense behavioural weight management programme and a 'simple' weight management programme compared to a control condition on school achievement in adolescents aged 10 to 19 years with a BMI > 95th percentile.
10. Robinson 2013 (StanfordGOALS) is taking place in the USA, aimed at children aged seven to 11 years with a BMI > 85th percentile. The study evaluates the effect of a large‐scale, community‐based, interdisciplinary, multicomponent intervention involving physical activity and behaviour change counselling related to screen time, diet and physical activity on school achievement compared to standard care.
11. Sardinha 2014 is located in Portugal and compares two interventions (physical activity and physical activity plus weight management education) with standard practice in children aged 11 to 14 years. Outcome measures include mathematical achievement, language achievement (Portuguese and English), science achievement and body weight status. This study has been completed but outcome data have not yet been published.
12. Scherr 2014 (Shaping Healthy Choices) is being conducted in the USA, and evaluates a multicomponent school nutrition education programme versus control (not further defined) on science achievement in fourth‐grade children. The intervention is not solely aimed at children with obesity or overweight but waist circumference and body mass status are being assessed, yielding data to be included in a future update of this review.
13. Stanley 2016 (Jump Start) is taking place in Australia, targeting young children aged three to five years. The study evaluates the effect of a physical activity and motor skills intervention versus usual practice on inhibitory control, working memory, and attention. In addition, body weight status is being assessed, allowing the researchers to provide data for children with obesity or overweight specifically.

### Risk of bias in included studies

The Characteristics of included studies table provides the reasons for the judgements of risk of bias for each item. Figure 3 and Figure 4 illustrate the judgement for each risk‐of‐bias item across all included studies and for each included study, respectively.

**3:**
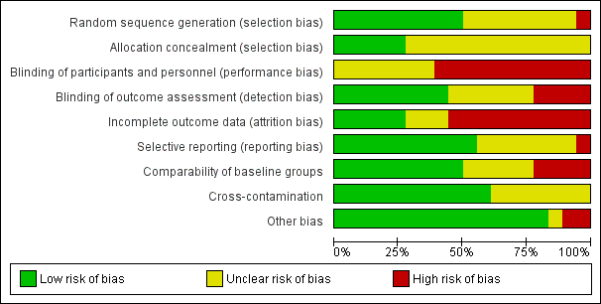
Risk of bias graph: review authors' judgements about each risk of bias item presented as percentages across all included studies.

**4:**
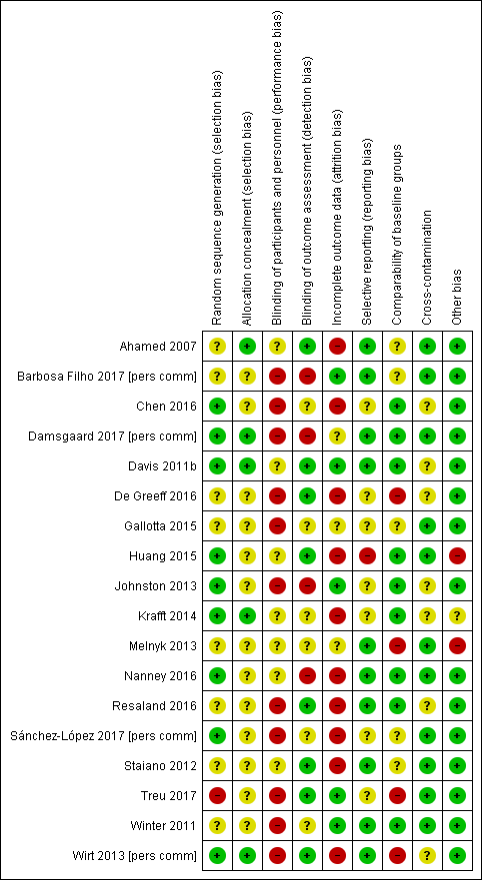
Risk of bias summary: review authors' judgements about each risk of bias item for each included study.

#### Allocation

The method of sequence generation was described adequately in eight studies and we rated these at low risk of bias. Eight studies failed to report sufficient details on how the random sequence was generated and we judged these studies to have an unclear risk of bias. Melnyk 2013 was also rated as unclear risk of bias despite adequate description of the sequence generation. However, it remains unclear if group allocation (drawing of school names from a hat) was sufficiently concealed using opaque envelopes. Treu 2017 was assessed at high risk of bias because only schools allocated to one of two intervention arms were randomised, whereas the control schools were not randomly allocated. Consequently, we conducted a sensitivity analysis.

Adequate description of allocation concealment was evident for five studies, and we judged these as low risk of bias. We rated all of the remaining 13 studies as unclear risk of bias, due to insufficient reporting.

#### Blinding

##### Blinding of participants and personnel (performance bias)

In trials involving physical activity, diet and health education, true blinding of participants and personnel involved in delivering the intervention is not possible. However, four studies (Ahamed 2007; Davis 2011b; Nanney 2016; Staiano 2012) blinded participants and personnel to the true purpose of the study relevant for this review, i.e. changes in cognitive or academic outcomes. We therefore judged these studies to be at unclear risk for performance bias. Three studies (Huang 2015; Krafft 2014; Melnyk 2013) employed an attention control condition which reduced the risk of performance bias and we rated these at unclear risk of bias. We rated the remaining 11 studies at high risk of bias.

##### Blinding of outcome assessment (detection bias)

We judged the risk of bias for blinding of the outcomes assessor as low for eight studies. Six studies reported insufficiently on whether the outcome assessor was blinded, and we therefore judged these as unclear risk of bias. School achievement was assessed by teachers who were aware of the group allocation in four studies (Barbosa Filho 2017 [pers comm]; Damsgaard 2017 [pers comm]; Johnston 2013; Nanney 2016) and so we rated these studies at high risk of detection bias.

#### Incomplete outcome data

We reported attrition rates and reasons for attrition in the Characteristics of included studies. Low levels of attrition and adequate handling of missing data were performed in five studies, which we rated at low risk of bias (Barbosa Filho 2017 [pers comm]; Davis 2011b; Johnston 2013; Treu 2017; Winter 2011). No imputation of missing data was evident in Damsgaard 2017 [pers comm], but attrition rates were low (less than 10%) and we judged this study as being at unclear risk of bias. Study details obtained from Gallotta 2015 were insufficient to assess the risk of attrition bias and thus we judged this study as being at unclear risk of bias. In Melnyk 2013, relevant outcome data were only collected at post‐intervention, which precluded assessment of attrition bias. We rated this study at unclear risk of bias. We judged the risk of attrition bias to be high in nine studies in which no imputation of missing data was performed or the level of attrition was high.

#### Selective reporting

We rated the risk of selective reporting as low in 10 studies, and unclear in seven studies which made no reference to a study protocol or trial register. We judged Huang 2015 to be at high risk of bias, because the cognitive outcomes and test batteries stated in the study protocol did not align with the Result report. According to the study protocol attention and processing speed were planned to be assessed using the Symbol Digit Modalities Test, and executive function and attention were planned to be assessed using the Trail Making Test. However, the Result report provided findings for executive function using the Stroop Colour and Word Test, and visuo‐spatial abilities and non‐verbal memory using the Rey complex Figure Test.

#### Other potential sources of bias

Comparability of baseline groups might be a potential source of bias in cluster‐RCTs, and RCTs with flaws in the randomisation procedure (Higgins 2011). Five cluster‐RCTs showed no difference between the experimental groups at baseline and we rated them at low risk of bias (Damsgaard 2017 [pers comm]; Johnston 2013; Nanney 2016; Resaland 2016; Winter 2011). We judged another five studies to be at unclear risk of bias (Ahamed 2007; Barbosa Filho 2017 [pers comm]; Gallotta 2015; Sánchez‐López 2017 [pers comm]; Wirt 2013 [pers comm]). There was evidence of between‐group differences at baseline in three studies, which we rated at high risk of bias (De Greeff 2016; Melnyk 2013; Treu 2017). Four of the five RCTs were at low risk of bias for random sequence generation and also reported no between‐group differences at baseline (Chen 2016; Davis 2011b; Huang 2015; Krafft 2014). We rated Staiano 2012 at unclear risk of bias for comparability of groups at baseline, because random sequence generation and allocation concealment were unclear and no formal assessment of the experimental groups at baseline was performed.

Cross‐contamination of the intervention to the comparison group or lack of adherence to the comparison condition might be a potential source of bias in RCTs. Cluster‐RCTs might be at risk of cross‐contamination where the units of randomisation were classes within the same school or where randomised schools were in close proximity. The risk of cross‐contamination was low in Huang 2015 and Staiano 2012. Due to insufficient reporting related to the adherence to the comparison condition, we rated the risk of bias as unclear in the remaining three RCTs (Chen 2016; Davis 2011b; Krafft 2014) and four cluster‐RCTs (De Greeff 2016; Johnston 2013; Resaland 2016; Wirt 2013 [pers comm]). The risk of cross‐contamination was low in the remaining nine cluster‐RCTs.

We identified two studies with other sources of bias. Huang 2015 included children that did not meet the inclusion criteria, so this study was at high risk of bias for violation of the study protocol. In Melnyk 2013, the school district administrator selected participating schools and the schools were offered financial incentives which might have introduced an additional selection bias. We did not detect any other risk of bias in the remaining studies and thus rated them at low risk of bias.

### Effects of interventions

See: Table 1; Table 2; Table 3

We summarised and analysed the three intervention groups in separate comparisons and generated a 'Summary of findings' table of the most important outcomes for each comparison (see Table 1; Table 2; Table 3). The intervention groups consisted of physical activity only, physical activity combined with healthy lifestyle education, and dietary interventions. We reported the secondary outcomes (future success and obesity indices) combined for all three comparisons, due to the low number of studies providing suitable data.

#### Primary outcomes

##### Comparison 1: Physical activity only interventions versus standard practice

Eight studies delivered physical activity‐only interventions and compared them to standard practice (see Table 4 and Characteristics of included studies). Of these, seven studies provided suitable data for inclusion in meta‐analyses. However, the number of studies included for the same class of outcome was low, ranging from one to three studies. We performed sensitivity analyses, as specified in Data collection and analysis. However, the low number of studies make the outcome of a sensitivity analysis less meaningful, as the number of included studies is reduced further. Data were available for the outcomes mathematics, reading and language achievement, and composite executive functions and inhibition control, which we include in Table 1. Study authors also provided data for the outcomes of attention, working memory, visuo‐spatial abilities, cognitive flexibility, non‐verbal memory, and general intelligence.

###### 1.1. School achievement

####### Mathematics achievement

Three studies were included which used different scales: broad mathematics scale of the Woodcook‐Johnson Test of Achievement III (Davis 2011b), a standardised national mathematics test (Resaland 2016), and numerical quantitative concepts scale of the General Differential Aptitude Battery (Sánchez‐López 2017 [pers comm]). We therefore calculated the effect estimate as the standardised mean difference. We calculated subtotals of change from baseline data from Resaland 2016 and Sánchez‐López 2017 [pers comm] (both cluster‐RCTs), and combined post‐intervention data from Davis 2011b (RCT) and Resaland 2016 separately. We converted the reported standard error for post‐intervention data in Davis 2011b into standard deviations.

Meta‐analysis findings (see Analysis 1.1)

Analysis of change from baseline data indicated 0.49 standard deviation higher mean mathematics achievement (95% confidence interval (CI) ‐0.04 to 1.01) in the physical activity group compared to standard practice (2 studies, 255 children, I^2^ = 57%, Tau^2^ = 0.09). We downgraded the quality of evidence by one level for high risk of attrition bias present in the two studies (Resaland 2016; Sánchez‐López 2017 [pers comm]). Pooled post‐intervention data resulted in a SMD of 0.19 (95% CI ‐0.03 to 0.42; 2 studies, 314 children, I^2^ = 0%, Tau^2^ = 0.00). Sensitivity analysis for high risk of attrition bias and cluster‐RCT design involved removing Resaland 2016 from the latter analysis. The overall conclusion of the evidence did not change with Davis 2011b remaining (SMD 0.31, 95% CI ‐0.10 to 0.71; 1 study, 96 children).

####### Reading achievement

Two studies provided data on the intervention effect of physical activity on reading achievement compared to standard practice. Both studies used different scales: broad reading scale of the Woodcook‐Johnson Test of Achievement III (Davis 2011b) and a standardised national reading test (Resaland 2016). We therefore calculated the standardised mean difference to estimate the pooled difference between the experimental groups. Resaland 2016 was a cluster‐RCT and Davis 2011b a RCT. Davis 2011b provided standard errors for the post‐intervention data which we converted into standard deviation scores prior to entering these in the meta‐analysis. We combined post‐intervention endpoint data.

Meta‐analysis findings (see Analysis 1.2)

Our analysis suggested that there was no statistically significant difference between physical activity and standard practice on reading achievement in children aged seven to 11 years with overweight, including obesity (SMD 0.10, 95% CI ‐0.30 to 0.49; 2 studies, 308 children, I^2^ = 63%, Tau^2^ = 0.05). This finding was of moderate quality and we downgraded it by one level due to high risk of attrition bias in Resaland 2016. Removing this study from the analysis did not change the conclusion (SMD 0.33, 95% CI ‐0.08 to 0.73; 1 study, 96 children).

####### Language achievement

This outcome was assessed by two studies. However, one study (Sánchez‐López 2017 [pers comm]) assessed native language achievement (Spanish) and another study provided data for English language achievement in people whose first language was Norwegian (Resaland 2016). We therefore did not combine these outcomes in a meta‐analysis, as different concepts were measured. For native language achievement, we reported the mean difference and standardised mean difference of the intervention effect, to allow comparison with studies included in Comparison 2 (physical activity combined with healthy lifestyle education versus standard practice).

There was no evidence of a beneficial effect of the physical activity programme Movi‐Kids (Sánchez‐López 2017 [pers comm]; see Table 4 for details) on native language achievement in children aged four to seven years with obesity or overweight (MD 2.38, 95% CI ‐4.75 to 9.51, scale range 0 to 36; SMD 0.23, 95% CI ‐0.50 to 0.95; 1 study, 31 children; Analysis 1.3). The quality of this evidence was low; we downgraded the quality twice for high risk of attrition bias and imprecision due to the low sample size. This outcome was measured using the analogical and complex verbal order scale of the General Differential Aptitude Battery.

Similarly, the Active Smarter Kids intervention (Resaland 2016; see Table 4 for details) did not yield improved second‐language achievements, assessed using standardised national tests, in 217 children aged 10 to 11 years with overweight (including obesity) compared to standard practice: MD 1.52, 95% CI ‐0.02 to 3.06; scale mean (SD) = 50 (10), see Analysis 1.4.

####### Additional educational support

None of the studies assessing the effect of physical activity interventions compared to standard practice in children with obesity or overweight reported findings on additional educational support needs.

###### 1.2. Cognitive function

####### Composite executive functions

Three studies measured composite executive functions, of which two studies provided suitable data for inclusion in the meta‐analysis. Krafft 2014 provided a narrative description of the findings only and we were not able to obtain the quantitative data from the study authors for inclusion in the meta‐analysis. Composite executive functions were measured using the Planning Scale of the Das‐Naglieri‐Cognitive Assessment System. The study authors reported that their eight‐month aerobic physical activity programme, delivered five days a week after school, did not result in statistically significant differences in composite executive functions compared to sedentary activities such as art and board games in 175 children aged eight to 11 years with obesity or overweight.

The two studies included in the meta‐analysis used different scales: Planning scale of the Das‐Naglieri‐Cognitive Assessment System (Davis 2011b), which is a composite of three separate tasks, and Design Fluency and Trail‐Making subscales of the Delis‐Kaplan Executive Function System (Staiano 2012). Both studies were RCTs, with one study reporting change from baseline data (Staiano 2012) and the other post‐intervention data (Davis 2011b). We therefore did not pool the two studies. Staiano 2012 included two intervention arms which we entered separately into the meta‐analysis. We divided the sample size of the control group by the number of intervention arms (i.e. two). We calculated mean differences and the standardised mean difference, to be able to compare the effect estimates between the two studies. We converted post‐intervention standard errors to standard deviation scores from Davis 2011b.

Meta‐analysis findings (see Analysis 1.5)

Analysis of post‐intervention data suggested that the mean composite executive functions were five scale points higher (95% CI 0.68 to 9.32; scale mean = 100, SD = 15; SMD 0.42, 95% CI 0.05 to 0.78) in the after‐school physical activity intervention group compared to standard practice in children aged eight to 11 years with obesity or overweight (1 study, 116 children). This evidence was of high quality. There was no evidence of a beneficial effect of exergaming interventions on change in mean composite executive function compared to standard care in 54 adolescents (MD 8.45 points, 95% ‐1.67 to 18.56 points; 1 study, scale mean = 10, SD = 3; SMD 0.58, 95% CI ‐0.02 to 1.18). The quality of this evidence was low, due to a high risk of attrition bias and imprecision of the effect estimate.

####### Inhibition control

One physical activity study measured inhibition control using the Stroop Colour Word Test (De Greeff 2016). The authors provided unpublished data for children with obesity or overweight for a mid‐term assessment at six‐month follow‐up and post‐intervention data at 18‐month follow‐up. We reported both the mean difference and the standardised mean difference of the intervention effect to allow comparison with other intervention types reported in this review. We conduced separate analyses for each time point and included post‐intervention follow‐up outcome data in Table 1.

Compared to standard practice, there was no evidence of a beneficial effect of physically active mathematics and language lessons on inhibition control in children aged seven to nine years with obesity or overweight at either follow‐up time point. At six‐month follow‐up, the mean inhibition control was 0.35 scale points higher (95% CI ‐2.59 to 3.29, scale range 0 to 100; SMD 0.04, 95% CI ‐0.33 to 0.41, 112 children; Analysis 1.6) in the intervention group compared to standard practice. At post‐intervention, the group difference was small (MD ‐1.55, 95% CI ‐5.85 to 2.75, scale range 0 to 100; SMD ‐0.15, 95% CI ‐0.58 to 0.28; 1 study, 84 children). This finding was of very low quality, suggesting low confidence in the effect estimate. We downgraded the quality by three levels for high risk of selection and attrition bias, and imprecision due to the low sample size.

####### Attention

Three studies measured attention abilities using different scales: Attention scale of the Das‐Naglieri‐Cognitive Assessment System (Davis 2011b; Krafft 2014) and the D2‐R test of attention (Gallotta 2015). Two of the studies were suitable for inclusion in the meta‐analysis for which we reported the effect sizes as the standardised mean difference of post‐intervention data. Krafft 2014 did not provide data for inclusion in the meta‐analysis. Narrative description of the findings indicate no effect of an eight‐month aerobic physical activity programme, delivered five days a week after school, compared to sedentary activities in favour of the intervention in 175 children aged eight to 11 years with obesity or overweight (Krafft 2014).

Meta‐analysis findings (see Analysis 1.7)

Gallotta 2015 provided unpublished data for the subgroup with obesity/overweight for three measures of attention: processing speed, concentration, and performance quality. We included only concentration performance because it was the most comparable measure with Davis 2011b. The two studies included in the meta‐analysis differed in that one was a RCT of a 13‐week after‐school physical activity programme (Davis 2011b), and one was a five‐month cluster‐RCT with two intervention arms delivered in the primary/elementary school setting (Gallotta 2015; see Table 4 for details). We included both intervention arms separately in the meta‐analysis and divided the sample size of the comparison group between them.

There was no evidence of a beneficial effect of the physical activity interventions compared to standard practice for eight to 11 year‐olds with obesity or overweight (SMD 0.46, 95% CI ‐0.16 to 1.08; 2 studies, 157 children, I^2^ = 41%, Tau^2^ = 0.14). The sensitivity analysis for cluster‐RCT design resulted in a SMD of 0.15 (95% CI ‐0.22 to 0.51; 1 study, 116 children).

####### Working memory

Only De Greeff 2016 provided data (unpublished specifically for children with obesity/overweight) for verbal working memory at six‐month follow‐up (mid‐term) and 18‐month follow‐up (post‐intervention data), measured using the Digit Span Backward test. The authors also provided non‐verbal working memory data obtained from the Visual Span Backward test. See Analysis 1.8; Analysis 1.9.

There was no evidence of a beneficial effect of physically active mathematics and language lessons on verbal working memory in children aged seven to nine years with obesity or overweight compared to standard practice at either follow‐up time point. At six‐month follow‐up, the mean verbal working memory was 0.15 points higher (95% CI ‐0.49 to 0.79, scale range 0 to 100) in the intervention group compared to standard practice (113 children). At 18‐month follow‐up, the mean verbal working memory was 0.06 points lower (95% CI ‐0.99 to 0.87, scale range 0 to 100) in the intervention group compared to standard practice (84 children). Our analysis found similar results for non‐verbal working memory at six‐month follow‐up (MD 0.27, 95% CI ‐0.40 to 0.94, scale range 0 to 100; 111 children). At 18‐month follow‐up, i.e. immediately post‐intervention, mean non‐verbal working memory was 0.62 points lower (95% CI ‐1.23 to ‐0.01, scale range 0 to 100) in the intervention group compared to standard practice (83 children) .

####### Visuo‐spatial abilities

Three studies assessed visuo‐spatial abilities of children with obesity or overweight using different scales: Simultaneous processing scale of the Das‐Naglieri‐Cognitive Assessment System (Davis 2011b; Krafft 2014) and the logical puzzle figures test of the General Differential Aptitude Battery (Sánchez‐López 2017 [pers comm]).

Similar to the previous outcomes assessed by Krafft 2014, composite executive functions and attention, the narrative description of the findings indicated no beneficial effect of an eight‐month aerobic physical activity programme, delivered five days a week after school compared to sedentary activities on visuo‐spatial abilities in eight to 11 year‐olds with obesity or overweight. We did not combine the two studies that provided data because Davis 2011b provided baseline‐adjusted post‐intervention data, while Sánchez‐López 2017 [pers comm] provided unpublished change‐from‐baseline data. We converted the reported standard errors in Davis 2011b to standard deviation scores.

Mean change in visual‐spatial abilities was 4.71 scale points higher (95% CI 0.40 to 9.02 scale points, scale range 0 to 36) in the Movi‐Kids intervention group compared to standard practice in 39 children with obesity or overweight (SMD 0.70, 95% CI 0.03 to 1.37; Sánchez‐López 2017 [pers comm]; Analysis 1.10). There was no evidence of a beneficial intervention effect on post‐intervention visuo‐spatial abilities of an after‐school physical activity programme compared to standard practice in 116 children (MD 4.00, 95% CI ‐0.44 to 8.44, scale mean 100, SD 15; SMD 0.33, 95% CI ‐0.04 to 0.69, Davis 2011b).

####### Cognitive flexibility

We included two studies which used a similar scale, the Wisconsin Card Sorting Test, but different measures were reported. De Greeff 2016 reported an efficiency score which considered the number of errors and unused cards, whereas Chen 2016 reported the total number of errors only. We therefore calculated the standardised mean difference. To allow comparability in terms of measurement time points we used the six‐month follow‐up of De Greeff 2016 and excluded the 18‐month follow‐up from the analysis. The immediate post‐intervention follow‐up in Chen 2016 was three months. We conducted sensitivity analyses for the cluster‐RCT (De Greeff 2016).

Meta‐analysis findings (see Analysis 1.11)

The mean cognitive flexibility performance was 0.06 standard deviations lower (95% CI ‐0.37 to 0.25, I^2^ = 0%, Tau^2^ = 0.00) in the physical activity intervention group compared to standard practice, indicating no beneficial effect in favour of the intervention group (162 children). Both studies were at high risk for attrition bias. Sensitivity analysis for cluster‐randomisation did not change the overall conclusion (SMD 0.14, 95% CI ‐0.41 to 0.70, 1 study, 50 children).

####### Non‐verbal memory

Two studies assessed non‐verbal memory using the same scale (Successive processing scale of the Das‐Naglieri‐Cognitive Assessment System) and employing the same physical activity intervention (Davis 2011b; Krafft 2014). Only Davis 2011b reported quantitative data consisting of baseline‐adjusted post‐intervention outcomes. Their findings indicated that an aerobic physical activity programme, delivered for 13 weeks on five days a week after school, resulted in 3.00 points higher (95% CI 0.51 to 5.49, scale mean 100, SD 15, Analysis 1.12) mean non‐verbal memory compared to standard practice in children aged eight to 11 years with obesity or overweight (SMD 0.43, 95% CI 0.07 to 0.80, 116 children). This effect estimate suggested a small difference between the intervention and comparison groups.

####### General intelligence

Sánchez‐López 2017 [pers comm] was the only study which provided measures of general intelligence, using the General and Differential Aptitude Battery. The mean change from baseline was 17.14 points higher (95% CI 7.24 to 27.04, scale range 0 to 108) in the intervention group (Movi‐Kids, see Table 4 for details) compared to the standard practice group (34 children, see Analysis 1.13). We are moderately confident in the effect estimate but it is likely that further research may change the estimate. Sánchez‐López 2017 [pers comm] was at high risk for attrition bias and imprecision of the effect estimate. However, we upgraded the quality of evidence due to the large effect size.

###### 1.3. Adverse outcomes

No study reported data on adverse events while or after taking part in physical activity interventions.

##### Comparison 2: Physical activity interventions combined with healthy lifestyle education versus standard practice

In total, seven studies delivered physical activity combined with healthy lifestyle education interventions and compared them to standard practice (see Table 4 and Characteristics of included studies). All studies provided suitable data for inclusion in meta‐analyses. However, the number of studies included for the same outcome was low, ranging between one and three studies. We performed sensitivity analyses as specified in Sensitivity analysis. However, as with Comparison 1, the low number of studies makes the outcome of a sensitivity analysis less meaningful as the number of included studies is further reduced.

Data were available for the outcomes mathematics, reading and language achievement, and inhibition control, which we included in Table 2. Study authors also provided data for the average achievement across subjects taught at school, attention, visuo‐spatial abilities, and non‐verbal memory.

###### 2.1 School achievement

####### Average achievement across subjects taught at school

One study provided unpublished data for the average score of mathematics, reading and language, using the Canadian Achievement Test 3 (Ahamed 2007). The mean change in average school achievement was 6.37 grade points lower (95% CI ‐36.83 to 24.09, scale mean 500, SD 70) in the intervention group ('Action Schools! BC') compared to standard practice in 31 children aged seven to 11 years with obesity or overweight (SMD ‐0.18, 95% CI ‐0.93 to 0.58; Analysis 2.1). Ahamed 2007 was at high risk of attrition bias and at unclear risk of randomisation bias (Figure 4) and we therefore downgraded the evidence by two levels.

####### Mathematics achievement

The effects of physical activity intervention combined with healthy lifestyle education on mathematics achievement were assessed in three studies using different scales: Canadian Achievement Test 3 (Ahamed 2007), standardised national mathematics test (Barbosa Filho 2017 [pers comm]), and AIMSweb standardised Mathematics Concepts and Application Test (Treu 2017). The scale used by Treu 2017 measured mathematical problem‐solving skills. Although additional outcomes obtained from Mathematics‐Curriculum‐Based‐Measurement scale were also provided by Treu 2017, we did not include this outcome because data were not available from all participating schools. We used change from baseline for all studies and calculated the standardised mean difference. We included data for children with overweight separately from data of children with obesity (Barbosa Filho 2017 [pers comm]), and also included the two intervention arms in Treu 2017 separately. We divided the sample size of the comparison group to estimate group differences. All studies were cluster‐RCTs, and so we conducted sensitivity analysis for risk of bias only.

Meta‐analysis findings (see Analysis 2.2)

There was no evidence of a beneficial effect for the intervention on mathematics achievement compared to standard practice (SMD 0.02, 95% CI ‐0.19 to 0.22; I^2^ = 0%, Tau^2^ = 0.00) in 384 children and adolescents aged eight to 18 years. This finding was of very low quality, suggesting that the true effect is likely to be substantially different from the estimated effect and we are confident that further research will result in different estimates. We downgraded the quality for high risk of bias (sequence generation, blinding of the outcome assessor, attrition), inconsistency, and imprecision of estimates. Barbosa Filho 2017 [pers comm] provided separate data for 64 children with overweight and 35 children with obesity. The single study effect estimates were statistically non‐significant for both subgroups.

Sensitivity analysis for high risk of sequence generation in Treu 2017 indicated no changes to the overall conclusion (SMD ‐0.07, 95% CI ‐0.41 to 0.28, 2 studies, 140 children). Removing the studies with high risk of attrition bias did not influence the overall conclusion (SMD ‐0.03, 95% CI ‐0.43 to 0.38; 1 study, 99 participants).

####### Reading achievement

Two cluster‐RCTs were included using different scales: Canadian Achievement Test 3 (Ahamed 2007) and AIMSweb standardised Reading Curriculum Based Measurement (Treu 2017). We therefore calculated standardised mean differences of change from baseline data. Treu 2017 also provided data obtained from the MAZE reading test which we did not include, because the curriculum‐based measurement appeared to be more comparable with the outcome reported by Ahamed 2007. We included the two intervention arms in Treu 2017 separately and distributed the sample size of the comparison between them.

Meta‐analysis findings (see Analysis 2.3)

There was low‐quality evidence of no difference between the intervention and comparison groups for reading achievement (SMD 0.00, 95% CI ‐0.24 to 0.24; 2 studies, 284 children, I^2^ = 0%, Tau^2^ = 0.00). We downgraded the evidence for risk of bias and inconsistency of effect estimates, suggesting little confidence in the effect estimate. Sensitivity analysis of high risk of selection bias (Treu 2017) and attrition bias (Ahamed 2007) did not change the overall conclusion.

####### Language achievement

We included three cluster‐RCTs which measured language achievement on different scales: Canadian Achievement Test 3 for English language (Ahamed 2007), standardised national test in Portuguese language (Barbosa Filho 2017 [pers comm]), and Peabody Picture Vocabulary Test in English language (Winter 2011). All studies provided unpublished change‐from‐baseline data for native language achievement of children with overweight/obesity. We calculated standardised mean differences due to the difference in scales used. We included the data set with imputed missing data (last observation carried forward) in Winter 2011, and conducted a sensitivity analysis using per‐protocol data.

Meta‐analysis findings (see Analysis 2.4)

Compared to standard practice, the mean language achievement was 0.13 standard deviations higher (95% CI ‐0.12 to 0.39, I^2^ = 0%, Tau^2^ = 0.00) in interventions combining physical activity with healthy lifestyle education (244 participants). This evidence was of very low quality, due to imprecision in effect estimates and high risk of attrition, selection and detection bias. This indicated that our confidence in the effect estimate is limited and further research is very likely to change the estimate. Sensitivity analysis for attrition bias in one study (Ahamed 2007) and imputation of missing data (Winter 2011) did not change the overall conclusion: SMD 0.12 (95% CI ‐0.18 to 0.43; 2 studies, 173 children) and SMD 0.11 (95% CI ‐0.17 to 0.40, 3 studies, 197 participants).

Two studies provided separate data for children with overweight and children with obesity (Barbosa Filho 2017 [pers comm]; Winter 2011). For children with obesity, mean change in language achievement was 0.28 standard deviations higher (95% CI ‐0.20 to 0.77) in the intervention group compared to standard practice (70 children, 2 studies). The effect favouring the intervention group was lower in children with overweight (SMD 0.02, 95% CI ‐0.37 to 0.41, 103 children, 2 studies).

####### Health class achievement

Melnyk 2013 assessed the effect of the 15‐week COPE Healthy Lifestyle TEEN programme (see Table 4 for details) compared to an attention control (health topics other than physical activity and nutrition) on teacher‐assessed grades in health class courses of adolescents aged 14 to 16 years. The authors provided unpublished post‐intervention data separately for adolescents with overweight and adolescent with obesity, which we entered as two comparisons in the meta‐analysis. We calculated the between‐group mean difference.

The mean health class achievement was 0.05 scores lower (95% CI ‐0.38 to 0.29, scale range 0 to 4, 263 adolescents; Analysis 2.5) in the intervention group compared to the control group, suggesting a small statistically non‐significant difference in favour of the control group. There was no between‐group difference between 108 adolescents with overweight and 155 adolescents with obesity. We judged this study to be of moderate quality and reduced the quality rating due to high risk of bias for the comparability of the experimental groups at baseline and selection bias.

####### Additional educational support

No study provided data on the effects of physical activity plus healthy lifestyle education interventions on additional educational support needs for children and adolescents with obesity or overweight.

###### 2.2. Cognitive functions

####### Inhibition control

We included two studies which measured inhibition control on different scales: Stroop Colour and Word Test (Huang 2015) and the Go/No‐go test of the Attention test battery for children (Wirt 2013 [pers comm]). We therefore report the standardised mean difference. Since only post‐intervention data were available for Wirt 2013 [pers comm], we used the post‐intervention data reported by Huang 2015. This study also reported mid‐term outcomes at six‐week follow‐up. We used the immediate post‐intervention time point for combining the study with Wirt 2013 [pers comm]. We performed a sensitivity analysis for the cluster‐RCT (Wirt 2013 [pers comm]).

Meta‐analysis finding (see Analysis 2.6)

There was low‐quality evidence of lower mean inhibition control by 0.67 standard deviations (95% ‐1.50 to 0.16) in physical activity plus healthy lifestyle education intervention compared to standard practice/attention control in 110 children aged six to 13 years with obesity or overweight. We downgraded the evidence for high risk of attrition bias and selective reporting. The statistical heterogeneity was substantial (I^2^ = 68%, Tau^2^ = 0.25), most likely owing to methodological variability in the interventions and population characteristics (see Characteristics of included studies). The sensitivity analysis did not change the conclusion.

####### Attention

One study reported intervention effects on attention using the sustained attention scale of the Attention Test Battery for children (Wirt 2013 [pers comm]). For comparability of the effect estimates with Comparison 1 and 3, we report the estimates as the mean difference (see Analysis 2.7) and standardised mean difference.

Compared to standard practice, analysis of the unpublished data indicate no beneficial effect of physically active school breaks combined with healthy lifestyle education for one school year in 27 children with obesity or overweight aged six to eight years. The mean attention ability was 4.47 lower (95% CI ‐8.55 to ‐0.39, scale range 0 to 100) in the intervention group compared to the control group (SMD ‐0.71, 95% CI ‐1.54 to 0.12; Analysis 2.7). Imprecision due to the low sample size and high risk of attrition bias limit our confidence in the effect estimate.

####### Visuo‐spatial abilities

Huang 2015 was the only study that measured visuo‐spatial abilities in children with obesity or overweight, and used the copy trial of the Rey Complex Figure Test at six‐week follow‐up (mid‐term) and 13‐month follow‐up (post‐intervention). We calculated between‐group mean differences of post‐intervention data. We also report the published effect estimates of change from baseline expressed as fitted mean of standardised outcomes which were adjusted for sex and cohort.

Analysis of crude post‐intervention data suggested no statistically significant effect favouring the intervention group at six‐week follow‐up (MD 0.29 points, 95% CI ‐1.52 to 2.10; scale range 0 to 36, SMD 0.07, 95% CI ‐0.34 to 0.47; 94 children; Analysis 2.8) and 13‐month follow‐up (MD ‐0.45 points, 95% CI ‐2.58 to 1.68; scale range 0 to 36; SMD ‐0.09, 95% CI ‐0.52 to 0.33; 86 children). The quality of evidence at both time points was low, due to high risk of attrition and imprecision of effect estimates.

There was evidence of a beneficial effect on visuo‐spatial abilities in favour of the intervention compared to the attention control when expressed as fitted mean adjusted for sex and cohort at six‐week follow‐up (SMD 0.47, 95% CI 0.08 to 0.86, 94 children), indicating a moderate difference between the two experimental groups. The beneficial effect was not maintained at 13‐month follow‐up (SMD 0.21, 95% CI ‐0.26 to 0.67, 86 children).

####### Non‐verbal memory

Huang 2015 was the only study that measured non‐verbal memory and was part of the Odense Overweight Intervention Study. This study used the immediate recall trial of the Rey Complex Figure Test to measure non‐verbal memory at six‐week follow‐up (mid‐term) and 13‐month follow‐up (post‐intervention). We calculated mean differences and standardised mean differences of post‐intervention data, and report the published standardised and adjusted change from baseline of this study.

Mean non‐verbal memory was 2.05 points lower (95% CI ‐5.03 to 0.93; scale range 0 to 36; SMD ‐0.28, 95% CI ‐0.69 to 0.13; 94 children) in the intervention group compared to attention control at six‐week follow‐up when analysing post‐intervention data. At 13‐month follow‐up the MD was ‐3.42 points (95% CI ‐6.30 to ‐0.54; scale range 0 to 36; SMD ‐0.52, 95% CI ‐0.95 to ‐0.08; 86 children; Analysis 2.9). Huang 2015 was at high risk for attrition bias and the effect estimates indicate imprecision, which leaves us with limited confidence in the estimate. The true effect might be substantially different for the reported estimates.

The sex‐adjusted mean difference in change from baseline was 0.19 standard deviations higher (95% CI ‐0.10 to 0.48, 94 children) in the intensive day‐camp intervention group compared to the attention placebo control group which received a low‐intensity physical activity and health education intervention (see Table 4 for details) at six‐week follow‐up. At 13‐month follow‐up, there was also no evidence of beneficial effects of the intervention on non‐verbal memory compared to attention control in 86 children with obesity or overweight aged 12 to 13 years (SMD ‐0.005, 95% CI ‐0.35 to 0.34).

###### 2.3. Adverse outcomes

No study reported adverse outcome data for physical activity plus healthy lifestyle education interventions.

##### Comparison 3: Dietary interventions versus standard practice

Three studies compared dietary intervention with a standard practice (see Table 4 and Characteristics of included studies). Data were available for four outcomes which we include in the Table 3: average achievement across subjects taught at school (two studies), mathematics achievement (one study), language achievement (one study), and attention (one study). All studies were cluster‐RCTs and two studies provided unpublished data for children with obesity or overweight (Damsgaard 2017 [pers comm]; Nanney 2016).

###### 3.1. School achievement

####### Average achievement across subjects taught at school

Johnston 2013 and Nanney 2016 assessed the average school year performance of mathematics, reading and science scores by generating a Grade Point Average. School achievement was assessed by teachers in both studies, but the scales varied: scale range 0 to 4 in Nanney 2016, scale range 0 to 100 in Johnston 2013. We therefore calculated the standardised mean difference. Both studies reported change‐from‐baseline data. Nanney 2016 provided separate data for children with overweight and children with obesity, which we have included as separate subgroups in the meta‐analysis. We conducted a sensitivity analysis for per‐protocol data of Nanney 2016.

Meta‐analysis findings (see Analysis 3.1)

The mean average across subjects taught at school was 0.32 standard deviations higher (95% CI ‐0.07 to 0.70) in the dietary intervention groups compared to standard practice for 439 children and adolescents aged 7 to 17 years with obesity or overweight, suggesting a small statistically non‐significant difference between the experimental groups.

Given that the participants in Johnston 2013 had an average BMI in the 95th percentile, we performed a subgroup analysis for body weight status, classifying Johnston 2013 under the subgroup ‘children with obesity’. Considering data of children with obesity only, there was a moderate effect estimate of 0.45 standard deviation in favour of the intervention group (95% CI 0.25 to 0.66, 379 participants). There was no evidence of a beneficial effect of the intervention in children with overweight (SMD ‐0.17, 95% CI ‐0.70 to 0.36, 1 study, 55 participants). The subgroup analysis identified Nanney 2016 as the source of statistical heterogeneity, with the I^2^ statistic reduced from 62% to 0% (Tau^2^ 0.07 to 0.00). Formal testing indicated a significant subgroup difference (Chi^2^ = 4.60, P = 0.03). This finding was of low quality, indicating that further research is very likely to have an important impact on our confidence in the effect estimate and may change the estimate. We downgraded the quality due to high risks of detection and attrition bias.

The sensitivity analysis for per‐protocol data indicated a mean average across subjects taught at school of 0.30 standard deviations higher (95% CI 0.04 to 0.55; 2 studies, 422 children, I^2^ = 20%) in the intervention group compared to standard practice. The effect estimate for children with obesity decreased from moderate to small (SMD 0.34, 95% CI 0.05 to 0.63; 2 studies, 380 children). The effect estimate for children with overweight shifted in favour of the intervention group (SMD 0.03, 95% CI ‐0.59 to 0.64, 1 study, 42 children).

####### Mathematics achievement

Damsgaard 2017 [pers comm] assessed the effect of the New Nordic Diet compared to standard school meals on mathematics achievement, measured using standardised national tests. This study provided unpublished data for children with overweight and for children with obesity, which we entered separately in the meta‐analysis (see Analysis 3.2). For comparability with the effect estimates of Comparisons 1 and 2, we calculated both mean difference and standardised mean difference for change from baseline.

There was low‐quality evidence of no beneficial effect of the dietary intervention compared to standard practice on mathematics achievement (MD ‐2.18, 95% CI ‐5.83 to 1.47, scale range: 0 to 69; SMD ‐0.26, 95% CI ‐0.72 to 0.20) in 76 children aged nine to 11 years with obesity or overweight. We downgraded the quality for a high risk of detection bias and imprecision of the effect estimate, probably due to the small sample size. This indicates low confidence in the effect estimate and that further research is very likely to change the estimate. There was no difference in effect estimates for children with overweight and children with obesity.

####### Reading achievement

Damsgaard 2017 [pers comm] also measured reading achievement using standardised national tests. The mean change in reading achievement was 0.13 standard deviations higher (95% CI ‐0.35 to 0.61; MD 1.17, 95% CI ‐4.40 to 6.73, scale range: 0 to 108) in the intervention group compared to standard practice, indicating a small statistically non‐significant difference between the experimental groups (67 children, see Analysis 3.3). This finding was of low quality, as we downgraded the evidence for risk of detection bias and imprecision of the effect estimate, probably due to the small sample size. Inspection of the effect estimates for overweight and obesity suggested statistically non‐significantly higher standardised reading achievement in favour of the control group for children with obesity, while for children with overweight the effect estimate was in favour of the intervention.

####### Additional educational support

None of the studies assessing the effect of dietary interventions compared to standard practice in children with obesity or overweight reported findings on additional educational support needs.

###### 3.2. Cognitive functions

####### Attention

Attention performance was assessed by one study. Damsgaard 2017 [pers comm] measured attention using the D2‐R test of attention. We included concentration performance as a measure of attention and discharged processing speed to allow the comparison of the effect estimates with those under Comparison 1. We used the change from baseline of the unpublished data and calculated MD and SMD (see Analysis 3.4).

Compared to standard practice, there was no evidence of a beneficial effect of the New Nordic Diet on attention performance (MD 1.68, 95% CI ‐7.86 to 11.22, scale range:‐359 to 299; SMD 0.04, 95% CI ‐0.55 to 0.62; 61 children). The analysis suggests inconsistency in the effect estimates for children aged nine to 11 years with overweight and children with obesity: statistically non‐significant higher attention performance of children with obesity in the intervention group, and higher attention performance in control group children with overweight.

The quality of this evidence was low; high risk of detection bias and imprecision of the estimate resulted in downgrading of the evidence. Our confidence in the effect estimate is therefore limited and the true effect of dietary interventions may be substantially different.

###### 3.3. Adverse outcomes

No study reported data on adverse events resulting from participating in the dietary interventions.

#### Secondary outcomes for comparisons 1 to 3

##### 1. Future success

No study provided data on the effects of any physical activity interventions, physical activity plus healthy lifestyle education, and dietary interventions on future success, such as years of schooling, college enrolment or income for children and adolescents with obesity or overweight.

##### 2. Obesity indices

We assessed the effects of behavioural interventions on change from baseline in BMI z‐scores, total body fat and waist circumference for studies that provided suitable data. We reported the effect estimates on obesity indices by the following subgroups:

1. Beneficial intervention effect on school achievement;
2. No beneficial intervention effect on school achievement;
3. Beneficial intervention effect on cognitive functions;
4. No beneficial intervention effect on cognitive functions.

We performed this data synthesis descriptively, rather than combining the effect estimates of individual studies, because of substantial differences in intervention and outcome characteristics. We calculated individual study between‐group mean differences where unpublished data were made available.

###### Body mass index (BMI) z‐scores

Six studies (two RCTs and four cluster‐RCTs) provided change‐from‐baseline BMI z‐scores (Damsgaard 2017 [pers comm]; Davis 2011b; Huang 2015; Johnston 2013; Sánchez‐López 2017 [pers comm]; Treu 2017). Wirt 2013 [pers comm] reported post‐intervention BMI z‐scores. We estimated the effective sample size for the cluster‐RCTs and used an ICC of 0.01 for BMI based on Berry 2012. We plotted mean differences by subgroups relative to intervention effectiveness and outcome category (i.e. school achievement or cognitive function; see Figure 5; Analysis 4.1).

**5:**
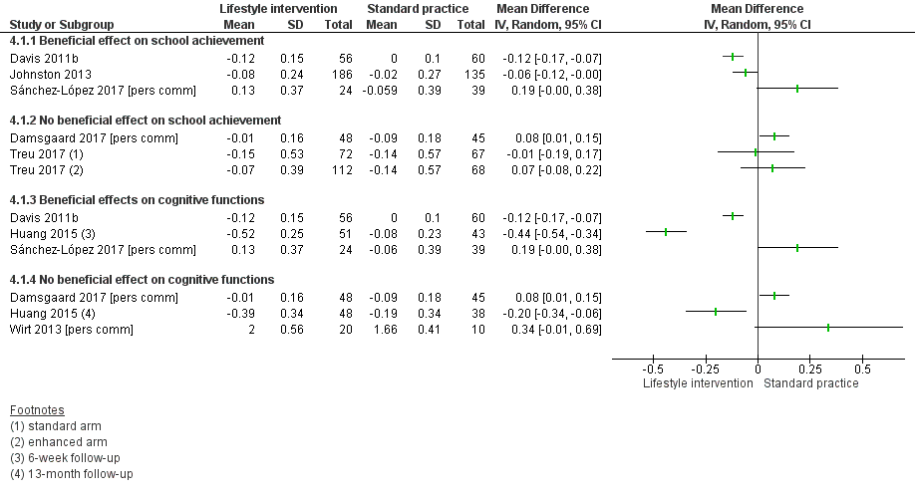
Forest plot of comparison: 4 Lifestyle intervention versus control, outcome: 4.1 BMI z‐score.

There was no evidence of a beneficial effect on change in BMI z‐scores of a school‐based physical activity intervention (Movi‐Kids, Sánchez‐López 2017 [pers comm]) compared to standard practice, despite increased school and cognitive outcomes (e.g. general intelligence) in favour of the intervention group. In fact, the change in BMI z‐score was in favour of the standard practice condition (MD 0.19, 95% 0.00 to 0.38; 62 children). In contrast, the after‐school physical activity intervention by Davis 2011b resulted in reduced BMI z‐scores in favour of the intervention group (MD ‐0.12, 95% CI ‐0.17 to ‐0.07; 116 children); the intervention resulted in improved mathematics attainment and cognitive functions (composite executive functions, non‐verbal memory) in the intervention group compared to the controls.

The physical activity plus health education intervention by Huang 2015 (Odense Overweight Intervention Study) was effective in reducing BMI z‐scores in favour of the intervention group at both follow‐up time points. At six‐week follow‐up, the intervention resulted in improved cognitive outcomes (visuo‐spatial abilities) and reduced BMI z‐score (MD ‐0.44, 95% CI ‐0.54 to ‐0.34; 94 children). At 13‐month follow‐up, there was no evidence of improved cognitive outcomes and, on average, children increased their BMI z‐score but less in the intervention group compared to standard practice (MD ‐0.20, 95% ‐0.34 to ‐0.06; 86 children). Both intervention arms of the complex physical activity plus healthy education intervention by Treu 2017 (ASCEND) resulted in no beneficial effect on BMI z‐scores in children with obesity or overweight compared to standard practice. This study also showed no beneficial effect on school achievement in favour of the intervention. Similarly, there was no evidence of a beneficial effect either on cognitive function (attention) or on post‐intervention BMI z‐scores in Wirt 2013 [pers comm] (MD 0.34, 95% CI ‐0.01 to 0.69; 30 children).

One dietary intervention, which resulted in improvements in school achievement reported a small reduction in BMI z‐scores change by 0.06 in favour of the intervention group (95% CI ‐0.12 to 0.00, 321 children; Johnston 2013). Another dietary intervention, which indicated no intervention benefits for school achievement or concentration performance, suggested a small reduction in BMI z‐score change by 0.08 in favour of standard practice (95% CI 0.01 to 0.15, 93 children; Damsgaard 2017 [pers comm]).

###### Total body fat percentage

We included three studies (see Figure 6; Analysis 4.2); one RCT (Chen 2016) and two cluster‐RCTs (Gallotta 2015; Huang 2015). We estimated the effective sample size of Gallotta 2015 using an ICC of 0.01 (Berry 2012).

**6:**
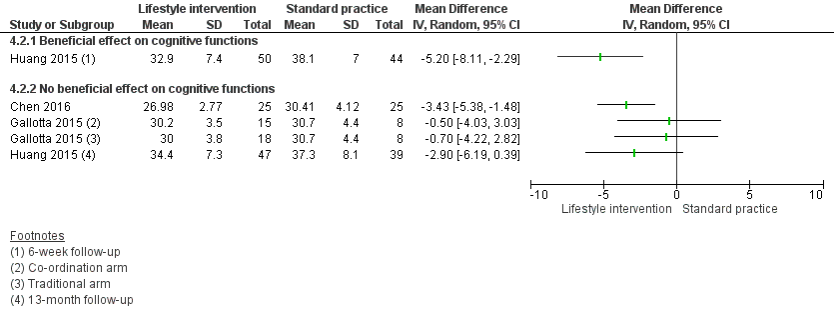
Forest plot of comparison: 4 Lifestyle intervention versus control, outcome: 4.2 Total body fat (%).

One study, which showed improved cognitive function in some domains of children with obesity or overweight after a six‐week day camp, indicated a mean reduction of 5.2% (95% CI ‐8.1% to ‐2.3%, 94 children) in total body fat in favour of the intervention compared to standard practice (Huang 2015). The statistically significant beneficial effects on cognitive functions and total body fat disappeared at 13‐month follow‐up (MD ‐2.90% 95% CI ‐6.19% to 0.39%, 86 children).

The two physical activity‐only interventions resulted in conflicting findings (Chen 2016; Gallotta 2015). Although both interventions suggested no beneficial effects on cognition in favour of the intervention group, Chen 2016 reported that the mean percentage body fat was 3.43% lower (95% CI ‐5.38% to ‐1.48%, 50 children) in the intervention group compared to standard practice/wait‐list control. Neither intervention arm in Gallotta 2015 showed evidence of a reduced total body fat compared to standard practice.

###### Waist circumference

Suitable data on change of waist circumference in children with obesity or overweight were available from only one study (Huang 2015). Improvements in cognitive function in favour of the intervention coincided with beneficial changes in waist circumference at six‐week follow‐up (MD ‐5.4 cm, 95% CI ‐7.4 cm to ‐3.5 cm; 94 children). At 13‐month follow‐up no beneficial effects on cognition or waist circumference were detected (MD ‐2.0 cm, 95% CI ‐4.5 cm to 0.6 cm; 86 children).

## Discussion

### Summary of main results

We identified five RCTs and 13 cluster‐RCTs evaluating the effectiveness of physical activity, dietary or other behavioural interventions for improving cognition and school achievement in children and adolescents with obesity or overweight. Eight studies offered a physical activity‐only intervention, seven studies combined physical activity with healthy lifestyle education, and three studies implemented a dietary intervention.

#### Physical activity only interventions

Based on a single study, there was high‐quality evidence for improvements in mean composite executive functions and non‐verbal memory when compared to continuation of usual activities. Offering school‐based extracurricular activities in combination with the restructuring of the playground environment indicated large benefits in mean general intelligence scores compared to standard practice. This finding was of moderate quality. No beneficial effects of physical activity interventions compared to standard practice were evident for mathematics, reading and language achievement, inhibition control, attention, cognitive flexibility, or visuo‐spatial abilities. The evidence of no effect was of moderate quality for mathematics and reading achievement and of very low quality for inhibition control.

#### Physical activity plus healthy lifestyle education

Combined physical activity and healthy lifestyle education interventions resulted in no improvements in the average achievement across subjects taught at school, mathematics achievement, reading achievement, health class achievement, inhibition control, attention, visuo‐spatial abilities and non‐verbal memory. The quality of the evidence of no effect was low to very low for all school achievement and cognitive outcomes.

#### Dietary interventions

Interventions targeting the improvement of the school food environment in conjunction with nutrition education resulted in a moderate difference in average achievement across subjects taught at school compared to standard practice in adolescents with obesity, but not in adolescents with overweight. However, the evidence was of low quality. There was no evidence that replacing packed school lunch with a diet rich in berries, root vegetables, whole grains and seafood (New Nordic Diet) improved attention, mathematics or reading achievement in children with obesity or overweight. This finding was also of low quality and further research is very likely to change the effect estimates.

#### Change in obesity by intervention effectiveness on school or cognitive outcomes

Based on our descriptive analysis, we were not able to detect a conclusive pattern linking improved school or cognitive outcomes with a reduction in obesity. Three studies indicated that highly‐intense interventions that involve daily exposure to physical activity or nutrition education, or both, can result in both significant change in obesity indicators and cognitive and academic outcomes compared to standard practice. However, one high‐intensity study that indicated a significant reduction in total body fat did not result in improved cognitive outcomes for the intervention group. Another study showed improved school attainment and cognitive functions but benefits on BMI z‐scores were not evident in the intervention group compared to standard practice.

The absence of an effect on school achievement or cognitive outcomes, or both, might be attributable to poor adherence to the experimental condition, particularly when the intervention was applied in participants' homes (e.g. physical activity homework tasks). Assessment of participants' compliance with the intervention was often poorly reported. We observed a similar bias for assessment of adherence to the control condition. Most studies did not attempt to evaluate or report whether the control group maintained its ‘standard practice’ during the trial period. For example, changes in school policy concerning healthy lifestyle factors such as improved school meals or physical activity opportunities during recess could potentially bias the intervention effects of experimental trials. The same may account for engagement in lifestyle changes at the family or child level.

The included studies provided no evidence of harm in terms of deterioration in any of the cognitive or school achievement outcomes. No data currently exist on whether lifestyle interventions for weight management of children and adolescents with obesity or overweight influence the need for additional educational support and indices of future success once schooling has been completed.

### Overall completeness and applicability of evidence

Our population group of interest ‐ children and adolescents with obesity or overweight ‐ is a very specific yet substantial and globally increasing subgroup of the general population. Of the 18 included studies, the study population of only six studies were children or adolescents with obesity or overweight. Most of the identified studies aimed to prevent obesity in the general population and did not report cognitive and academic outcomes of the subgroup with obesity or overweight separately from those of children in the healthy weight category. This was surprising, since 11 of the 18 studies stated cognitive function or school achievement among their primary outcomes. Despite our efforts to obtain them, the subgroup data for some studies have not been available to date (see Characteristics of studies awaiting classification). Based on our assessment of the quality of the evidence, we are confident that further research is likely to influence the estimates of the intervention effects for all assessed outcomes (see Quality of the evidence). Overall, the results of this review suggest applicability of the findings for public health practice for some but not all assessed outcomes (see Implications for practice).

Most of the included studies were conducted in the primary/elementary school setting. Only two studies contributed to the evidence on intervention effects in preschool‐aged children and five studies targeted adolescents enrolled in secondary (junior high/high) school. One plausible reason for this imbalance might be that primary/elementary‐school‐aged children seem old enough to understand instructions and young enough to comply with the intervention protocol. The influence of puberty on cognitive development might also contribute to more researchers focusing on pre‐pubertal adolescents (Juraska 2014). However, the developmental trajectories of cognitive abilities related to school achievement span preschool age and late adolescence (Boelema 2014; Davidson 2006; Waber 2007), and differential effects of behaviour change interventions at different ages are plausible. The overall low number of studies included for each outcome did not allow us to formally test the effectiveness of the intervention by age group. Nevertheless, we identified two ongoing trials in preschool‐aged children (Po'e 2013; Stanley 2016) and two in adolescents (Bau 2016; RBR‐38p23s) which assessed intervention effects on cognitive and academic outcomes in participants with obesity or overweight.

All but one (Barbosa Filho 2017 [pers comm]) of the included studies were conducted in high‐income countries and most studies (14/18 studies) included children primarily from middle‐income families. The reported evidence might therefore not be applicable to low‐ and middle‐income countries. In addition, a potentially differential effect of physical activity, diet and other behavioural interventions on cognition and school achievement of children with obesity or overweight growing up in a socio‐economically deprived environment remains to be investigated. The evidence on the association between obesity and poverty (Hardy 2017; Lissner 2016; Wang 2012), and the associations between lower education and cognitive skills and poverty (Cooper 2013; Marteau 2013) support further efforts in identifying who could benefit most from obesity‐related health behaviour interventions.

There was no evidence available on the effect of interventions targeting the quality and duration of sleep, or sedentary behaviour, or both, despite its association with obesity and impaired cognitive or academic performance or both. However, the healthy lifestyle education component of Barbosa Filho 2017 [pers comm] and Wirt 2013 [pers comm] included lessons on reducing media screen time, and Ahamed 2007, De Greeff 2016, Johnston 2013 and Resaland 2016 delivered physically active classroom lessons. Both education on reducing media screen time and physically active lessons might be considered as an intervention component to decrease sedentary behaviour (time spent sitting). In addition, one feasibility trial is currently ongoing, testing whether reduced sitting time in school can improve cognitive outcomes (‘Stand Up For Health’ study, trial register: ACTRN12614001001684). Although this trial listed obesity/overweight as a target health condition, the trial register entry did not mention assessment of change in adiposity.

Although two studies provided outcome data for two follow‐up time points (De Greeff 2016; Huang 2015), the data related to mid‐ and immediately post‐intervention. Participants in Huang 2015 received a low‐intensity maintenance intervention after completion of the intense six‐week day camp. We therefore could not fully explore the retention effect of interventions for weight management on school achievement and cognitive functions in children and adolescents with obesity or overweight. Nevertheless, the findings of Huang 2015 indicate that the beneficial effect of the day‐camp intervention compared to attention control on visuo‐spatial abilities was not maintained after completion of the 13‐month family‐based maintenance intervention.

It remains unclear whether changes in academic and cognitive abilities were connected to changes in indices of obesity, due to the small amount of suitable data, and variations in study architecture (Davis 2011b; Huang 2015; Johnston 2013).

### Quality of the evidence

We separately assessed the quality of evidence of the most important outcomes for decision‐making for each comparison of the three intervention types (see Table 1; Table 2; Table 3).

The quality of evidence for Comparison 1 ‐ physical activity‐only interventions compared to standard practice ‐ was high to very low. The reason for downgrading the evidence on mathematics achievement and reading achievement was a high risk of attrition bias. The attrition rate was 14% to 16% in most of the studies contributing to the evidence. No imputation of missing data was performed and we found higher attrition in the comparison condition compared to the intervention group. We downgraded the quality of evidence for inhibition control by three levels, for high risk of attrition and selection bias and for imprecision. Missing outcome data were not accounted for and the sample sizes were 31 participants for language achievement and 84 for inhibition control. For inhibition control, the method of randomisation was unclear, with a high risk of bias in the comparability of groups at baseline.

The quality of evidence for Comparison 2 – physical activity plus healthy lifestyle education intervention compared to standard practice – was low to very low. We downgraded the quality of evidence for mathematics achievement by three levels for inconsistency of the effect estimates, imprecision of the effect estimate and methodological shortcomings related to a high risk of bias for sequence generation, blinding of outcome assessors and attrition. We downgraded the quality of evidence for reading by two levels, for effect estimates of reading achievement being inconsistent between studies and for risk of bias in the study methodology. One of the two included studies was at high risk of bias for sequence generation and one study was at high risk of attrition bias, with 29% of incomplete outcome data. We downgraded the quality of evidence for inhibition control by two levels because we detected selection bias and a high risk of attrition bias characterised by twice as much missing outcome data in the control group compared to the intervention group (26% versus 13%).

The quality of evidence for Comparison 3 – dietary intervention compared to standard practice ‐ was low for average achievement across subjects taught at school, mathematics and reading achievements and attention performance. We downgraded the quality of evidence for average achievement across subjects taught at school by two levels for methodological shortcomings in blinding of the outcome assessor and for attrition bias (21% to 29%). We downgraded the quality of the remaining outcomes by two levels for imprecision (the sample sizes ranged between 76 and 61 children) and for not blinding the outcome assessor.

### Potential biases in the review process

We searched 17 electronic databases, two trial registers and handsearched one journal to identify published and ongoing trials. We also contacted 15 trial authors to obtain unpublished data and obtained unpublished outcome data from 12 studies. However, we acquired adverse‐events data only from published records.

Nevertheless, we intended to review evidence in a specific subgroup of the general population; the following limitation should therefore be considered. The unpublished data provided by the study authors were extracted for a subgroup of the total study sample, leading to overall small sample sizes for inclusion in this review. This might have affected the studies' power to detect an intervention effect. Studies which provided unpublished data for the subgroup of children with obesity or overweight may have been powered for the total study sample.

Included studies used a wide range of school achievement and cognitive function test tools. Previous reviews, such as that of Smith 2011, suggest that obesity might have a detrimental impact on some aspects of cognition, so we conducted a categorisation of outcome measures. The use of composite scores in some studies precluded more fine‐grained synthesis. For example, the planning subscale of the cognitive assessment system (CAS) is a composite score from three different measures of executive function, none of which are comparable to more traditional measures of planning such as the Tower of London task. As composite scores were reported in some cases, we categorised outcome measures as 'general executive function', rather than more discrete aspects of executive function (e.g. inhibition). Alternative categorisation of cognitive outcomes might impact on the conclusions drawn. Even though there tend to be correlations between cognitive function tests (because of the general cognitive factor *g*), different cognitive tests vary in their specificity for different cognitive domains. Moreover, successive testing before and after the intervention is likely to improve participant scores through repeated measures and regression to the mean. Thus, an improvement may not be due to the intervention, although the use of change‐from‐baseline data and the use of a comparison group allows some control for this. On the other hand, small participant numbers limit the ability to minimise bias.

### Agreements and disagreements with other studies or reviews

To our knowledge, no primary studies have been conducted other than those reviewed here. The systematic literature review by Bustamante 2016 narratively synthesised the evidence of physical activity interventions on cognitive and academic outcomes in youth with obesity or overweight. The authors included quasi‐experimental and randomised controlled trials published in peer‐reviewed journals before December 2015. The literature search was conducted in three selected electronic databases (PubMed, Journals@OVID, and Web of Science). Five RCTs were included in the review, of which we include four in this Cochrane Review (Davis 2011b; Huang 2015; Krafft 2014; Staiano 2012). We did not include the remaining RCT (Crova 2014) because the study did not aim to modify body weight status and so was not eligible for inclusion. The quasi‐experimental studies included in Bustamante 2016 were uncontrolled single‐group trials, case‐control studies or short, single‐session (acute bout) physical activity interventions, which we did not consider eligible for inclusion in this review. Bustamante 2016 concluded that, based on a single RCT, regular physical activity was more beneficial for improving executive functions than monthly lifestyle education classes (Davis 2011b). This finding is consistent with our results.

Bustamante 2016 argued that when regular physical activity interventions are compared to an attention control activity that involved organised activities supervised by adults, the beneficial effect of the physical activity intervention on academic and cognitive outcomes (detected using psychometric test batteries) is outweighed by the attention received in the comparison group (Krafft 2014). Findings of our evidence synthesis suggest otherwise. Studies that compare physical activity interventions with standard practice, which typically also involve organised activities supervised by adults (i.e. teachers), resulted in significant improvements in academic and cognitive outcomes (e.g. Resaland 2016; Sánchez‐López 2017 [pers comm]). Furthermore, the comparison condition in Huang 2015 was an active intervention providing attention to participants. Huang 2015 demonstrated a beneficial intervention effect on some cognitive skills compared to attention control.

Several systematic reviews are available on the effects of physical activity (Donnelly 2016; Fedewa 2011; Sibley 2003, Vazou 2016; Verburgh 2014), dietary (Ells 2008) and general school health interventions (Langford 2014; Murray 2007) on school achievement and cognitive outcomes in the general population. Although these systematic reviews may include some children with obesity or overweight, they lack a separate analysis of the effect estimates in our population groups of interest. Research suggests a greater benefit of obesity‐related health behaviour interventions in children with obesity or overweight compared to children with healthy weight (Crova 2014; Grieco 2009; Vazou 2014). These reviews are therefore not directly comparable with our review.

## Authors' conclusions

**Implications for practice**

This review provides some evidence that interventions which promote physical activity may be effective in producing small improvements in composite executive functions and non‐verbal memory in primary/elementary school‐aged children with obesity or overweight specifically. However, this evidence is based on a small number of studies. On current evidence, we are unable to determine the impact of these interventions on school achievement or cognitive skills. The current evidence on the effectiveness of interventions that combine healthy lifestyle education with physical activity promotion and dietary interventions does not allow us to draw definitive conclusions on their impact on cognitive and academic outcomes. In the absence of data, it is not possible to determine the impact of physical activity, dietary and other behavioural interventions on additional educational support, adverse events or outcomes related to future educational achievements such as years of schooling, employment rates or income.

Evidence on the effects of physical activity or dietary interventions on school achievement and cognitive functions in children with obesity or overweight conducted in clinical settings (e.g. hospitals, outpatient clinics, primary care) is missing, so we cannot offer implications for clinical practice in settings beyond school and community settings.

**Implications for research**

We identified studies in school, after‐school and community settings, but we found no evidence on cognitive and academic outcomes of behavioural weight management interventions in a clinical setting. However, our findings indicate beneficial effects of physical activity interventions on cognitive outcomes, namely cognitive executive functions, in children with obesity or overweight. Cognitive executive functions have been associated with the ability to control food intake (Bartholdy 2016; Jansen 2015) and engagement in health behaviour (Hall 2014). Child and adolescent weight management programmes in a clinical setting should include measurements of cognitive outcomes for two reasons. Firstly, the most effective strategies for weight management could be informed when linking cognitive abilities with behaviour change. Secondly, children with obesity or overweight are the target population of weight management programmes in clinical settings. If studies of interventions in clinical settings were to include measures of cognitive outcomes and related school achievement, these would help to boost the power of studies to identify potential gains in these areas. Similarly, community‐based interventions which directly target children and adolescents with obesity and which assess cognitive and academic outcomes are needed to advance the evidence. In addition, the availability of larger studies might allow the assessment of a differential intervention effect for participants with overweight and participants with obesity in relation to school achievement and cognitive functions.

In terms of the targeted obesity‐related health behaviours, evidence was available for solely physical activity interventions, physical activity plus healthy lifestyle education interventions and dietary interventions, which also included nutrition education. Our findings suggest that interventions focusing on one target behaviour, i.e. physical activity, yielded beneficial effects on composite executive functions, non‐verbal memory and general intelligence compared to standard practice. In contrast, interventions targeting several health behaviours through healthy lifestyle education and active physical activity programmes did not result in beneficial effects on these outcomes compared to standard practice. It might be that the positive effect of the physical activity programme on those cognitive functions is diluted with increasing complexity of the interventions. The intensity of the physical activity component might be reduced when additional intervention activities, such as healthy lifestyle education sessions, are implemented. Adjustments to the duration and frequency of physical activity programmes might have been required to keep the burden on the school personnel manageable. While interventions with multiple strategies appear successful for obesity prevention and treatment (Al‐Khudairy 2017; Colquitt 2016; Mead 2017; Waters 2011), a sufficient intensity and quality of the effective intervention components might be required for improving cognitive functions. We were not able to provide a similar observation with dietary intervention because none of the included studies applied an intervention without an additional nutrition education programme.

Given the importance of adequate physical and cognitive development of young children for their later life, further evidence is needed on the effectiveness of physical activity, dietary and other behavioural interventions on cognition and school achievement in the preschool years. In addition, the evidence is insufficient for adolescents who have reached puberty. The effectiveness of obesity‐related behaviour change interventions on cognition and school achievement in this age group is of particular importance, because of the direct implications for adult health and socio‐economic success of the individual and the nation. The extent to which sex and ethnicity influence the effect of physical activity and dietary interventions on cognition and school achievement in children and adolescents with obesity or overweight remains unknown, and should be addressed in future research.

Future multicomponent obesity prevention and treatment programmes should consider implementing physical activity programmes which are effective in improving cognitive functions or school achievement.

Further research is needed in low‐ and middle‐income settings, to establish whether there are differential intervention effects on cognition and school achievement for children and adolescents with obesity or overweight living in socio‐economically deprived environments. The educational, societal and economic argument for implementing effective childhood obesity prevention and treatment programmes could be substantial.

Longer‐term follow‐up trials are needed to determine whether improvements in school achievement and cognitive function are sustainable over time and thus affect future success. High rates of loss to follow‐up assessment are a common problem in lifestyle interventions, particularly those involving children and adolescents with obesity or overweight. To reduce the risk of attrition bias, researchers might wish to consider methods to impute missing outcome data in their analysis and to report characteristics of and reasons for missing data.

Including brain‐imaging techniques might enable researchers to detect beneficial effects on cognition which are not detectable using psychometric tests of academic and cognitive abilities. Finally, more multivariate research is needed to further investigate associations, two‐way interactions and causal pathways between childhood obesity, lifestyle behaviour, cognitive abilities and academic outcomes.

## What's new

**Table:** 

**Date**	**Event**	**Description**
8 February 2018	New citation required but conclusions have not changed	Republished for immediate open access.

## History

Protocol first published: Issue 3, 2012 Review first published: Issue 3, 2014

**Table:** 

**Date**	**Event**	**Description**
21 July 2017	New search has been performed	Updated following a new search in February 2017.
21 July 2017	New citation required and conclusions have changed	We identified eligible dietary interventions that allowed us to draw conclusions about their effectiveness on school achievement. Evidence was available for achievement in additional school subjects and cognitive abilities. We included 12 new studies.

## Appendices

### Appendix 1. Search strategies

#### Cochrane Central Database of Controlled Trials (CENTRAL), in the Cochrane Library which includes the Cochrane Developmental, Psychosocial and Learning Problems Specialised Register

2012 Issue 2 searched on 2 March 2012 (2145 records) 2013 Issue 4 searched on 8 May 2013. Limited to publication year = 2012 to 2013 (98 records) 2017 Issue 1 searched on 02 February 2017: Limited to publication year = 2013 to 2017 (1854 records)

#1 MeSH descriptor Overweight explode all trees #2 MeSH descriptor Body Weight, this term only #3 (obes* or overweight or over‐weight) #4 MeSH descriptor Body Weight Changes explode all trees #5 (weight near/2 (loss or lost or losing or reduc*)) #6 (weight near/2 (gain* or increas*)) #7 MeSH descriptor Body Fat Distribution explode all trees #8 MeSH descriptor Body Mass Index explode all trees #9 MeSH descriptor Skinfold Thickness explode all trees #10 MeSH descriptor Waist‐Hip Ratio explode all trees #11 ("body weigh*" or bodyweigh* or "body mass*" or bodymass or "body fat*" or bodyfat*) #12 MeSH descriptor Overnutrition, this term only #13 (overeat* or over‐eat* or overnourish* or over‐nourish* or overnutrit* or over‐nutrit*) #14 (#1 OR #2 OR #3 OR #4 OR #5 OR #6 OR #7 OR #8 OR #9 OR #10 OR #11 OR #12 OR #13) #15 MeSH descriptor Child explode all trees #16 MeSH descriptor Adolescent, this term only #17 (child* or schoolchild* or preschool* or pre‐school* or schoolage* or school‐age* or schoolboy* or schoolgirl* or boy* or girl* or preteen* or teen* or adolescen* or youth* or "young people" or "young person*" or pediatr* or paediatr*) #18 (#15 OR #16 OR #17) #19 MeSH descriptor Exercise, this term only #20 MeSH descriptor Exercise Therapy, this term only #21 MeSH descriptor Physical Exertion, this term only #22 MeSH descriptor Motor Activity, this term only #23 MeSH descriptor Sports, this term only #24 (sport*) #25 MeSH descriptor Physical Education and Training explode all trees #26 (physical near/3 (activit* or education* or exertion* or training)) #27 (exercise*) #28 MeSH descriptor Diet Therapy explode all trees #29 ((diet or dieting) near/5 (health* or weight*)) #30 (calorie near/3 (control or reduc* or restriction)) #31 "food choice*" #32 ("fat camp*" or "weight loss camp*") #33 "nutrition education" #34 MeSH descriptor Nutrition Therapy, this term only #35 MeSH descriptor Behavior Therapy, this term only #36 MeSH descriptor Cognitive Therapy, this term only #37 MeSH descriptor Psychotherapy, this term only #38 (behavio?r* near/3 (therap* or technique* or modif* or intervention*)) #39 (cognit* near/3 (therap* or technique* or modif* or intervention*)) #40 CBT #41 (psychotherap* or psycho‐therap*) #42 MeSH descriptor Family Therapy, this term only #43 (family near/3 (therap* or intervention*)) #44 family‐based #45 MeSH descriptor Sedentary Lifestyle, this term only #46 sedentary near/3 (lifestyle or behavio?r*)) #47 MeSH descriptor Video Games, this term only #48 MeSH descriptor Television, this term only #49 (television or tv) #50 "screen time" #51 (psycho‐social or psychosocial) #52 MeSH descriptor Health Promotion explode all trees #53 MeSH descriptor Health Education, this term only #54 (health* near/3 (promot* or educat* or lifestyle)) #55 MeSH descriptor Life Style, this term only #56 (lifestyle* or life‐style*) #57 ((video or computer) next game*) #58 (#19 OR #20 OR #21 OR #22 OR #23 OR #24 OR #25 OR #26 OR #27 OR #28 OR #29 OR #30 OR #31 OR #32 OR #33 OR #34 OR #35 OR #36 OR #37 OR #38 OR #39 OR #40 OR #41 OR #42 OR #43 OR #44 OR #45 OR #46 OR #47 OR #48 OR #49 OR #50 OR #51 OR #52 OR #53 OR #54 OR #55 OR #56 OR #57) #59 (#14 AND #18 AND #58)

#### Ovid MEDLINE

1950 to 17 February 2012, searched 22 February 2012 (2145 records) 1946 to Week 4 April 2013, searched 7 May 2013, Limited to ED=20120217‐20130507 (1009 records) 1946 to January Week 4 2017, searched 2 February 2017, Limited to publication year = 2013 ‐ 2017 (3078 records)

1     exp Overweight/ 2     Body Weight/ 3     (obes$ or overweight or over‐weight).tw. 4     exp Body Weight Changes/ 5     (weight adj2 (loss or lost or losing or reduc$)).tw. 6     (weight adj2 (gain$ or increas$)).tw. 7     exp body fat distribution/ or body mass index/ or skinfold thickness/ or waist‐hip ratio/ 8     (body weigh$ or bodyweigh$ or body mass$ or bodymass or body fat$ or bodyfat$).tw. 9     Overnutrition/ 10     (overeat$ or over‐eat$ or overnourish$ or over‐nourish$ or overnutrit$ or over‐nutrit$).tw. 11     or/1‐10 12     exp Child/ 13     Adolescent/ 14     (child$ or schoolchild$ or preschool$ or pre‐school$ or schoolage$ or school‐age$ or schoolboy$ or schoolgirl$ or boy$ or girl$ or preteen$ or teen$ or adolescen$ or youth$ or young people or young person$ or pediatr$ or paediatr$).tw. (1087380) 15     12 or 13 or 14 16     Exercise/ or Exercise Therapy/ 17     Physical Exertion/ 18     Motor Activity/ 19     Sports/ 20     sport$.tw. 21     exp "Physical Education and Training"/ 22     (physical adj3 (activit$ or education$ or exertion$ or training)).tw. 23     exercise$.tw. 24     exp diet therapy/ 25     ((diet or dieting) adj5 (health$ or weight$)).tw. 26     (calorie adj3 (control or reduc$ or restriction)).tw. 27     food choice$.tw. 28     (fat camp$ or weight loss camp$).tw. 29     nutrition education.tw. 30     Nutrition Therapy/ 31     behavior therapy/ 32     Cognitive Therapy/ 33     psychotherapy/ 34     (behavio?r$ adj3 (therap$ or technique$ or modif$ or intervention$)).tw. 35     (cognit$ adj3 (therap$ or technique$ or modif$ or intervention$)).tw. 36     CBT.tw. 37     (psychotherap$ or psycho‐therap$).tw. 38     family therapy/ 39     (family adj3 (therap$ or intervention$)).tw. 40     family‐based.tw. 41     sedentary lifestyle/ 42     (sedentary adj3 (lifestyle or behavio?r$)).tw. 43     video games/ 44     television/ 45     (television or tv).tw. 46     "screen time".tw. 47     (psycho‐social or psychosocial).tw. 48     exp Health Promotion/ 49     Health Education/ 50     (health$ adj3 (promot$ or educat$ or lifestyle)).tw. 51     lifestyle/ 52     (lifestyle$ or life‐style$).tw. 53     ((video or computer) adj game$).tw. 54     or/16‐53 55     11 and 15 and 54 56     randomized controlled trial.pt. 57     controlled clinical trial.pt. 58     randomi#ed.ab. 59     placebo$.ab. 60     drug therapy.fs. 61     randomly.ab. 62     trial.ab. 63     groups.ab. 64     or/56‐63 65     exp animals/ not humans.sh. 66     64 not 65 67     55 and 66

#### Ovid MEDLINE Epub Ahead of Print

Searched 2 February 2017 (275 records)

1 (obes$ or overweight or over‐weight).tw. 2 (weight adj2 (loss or lost or losing or reduc$)).tw. 3 (weight adj2 (gain$ or increas$)).tw. 4 (body weigh$ or bodyweigh$ or body mass$ or bodymass or body fat$ or bodyfat$).tw. 5 (overeat$ or over‐eat$ or overnourish$ or over‐nourish$ or overnutrit$ or over‐nutrit$).tw. 6 or/1‐5 7 (child$ or schoolchild$ or preschool$ or pre‐school$ or schoolage$ or school‐age$ or schoolboy$ or schoolgirl$ or boy$ or girl$ or preteen$ or teen$ or adolescen$ or youth$ or young people or young person$ or pediatr$ or paediatr$).tw. 8 6 and 7 9 sport$.tw. (8 10 (physical adj3 (activit$ or education$ or exertion$ or training)).tw. 11 exercise$.tw. (2 12 ((diet or dieting) adj5 (health$ or weight$)).tw. 13 (calorie adj3 (control or reduc$ or restriction)).tw. 14 food choice$.tw. 15 (fat camp$ or weight loss camp$).tw. 16 (behavio?r$ adj3 (therap$ or technique$ or modif$ or intervention$)).tw. 17 (cognit$ adj3 (therap$ or technique$ or modif$ or intervention$)).tw. 18 CBT.tw. 19 (psychotherap$ or psycho‐therap$).tw. 20 (family adj3 (therap$ or intervention$)).tw. 21 family‐based.tw. 22 (sedentary adj3 (lifestyle or behavio?r$)).tw. 23 (television or tv).tw. 24 "screen time".tw. 25 ((video or computer) adj game$).tw. 26 (psycho‐social or psychosocial).tw. 27 (lifestyle$ or life‐style$).tw. 28 (health$ adj3 (promot$ or educat$)).tw. 29 (multi‐component$ or multiple component$).tw. 30 or/9‐29 31 8 and 30 32 (Random$ or trial$ or control$ or placebo$ or blind$ or prospectiv$ or meta‐analysis or group or systematic review).tw. 33 31 and 32

#### Ovid MEDLINE In‐Process & Other Non‐Indexed Citations

Searched 2 February 2017 (918 records)

1 (obes$ or overweight or over‐weight).tw. 2 (weight adj2 (loss or lost or losing or reduc$)).tw. 3 (weight adj2 (gain$ or increas$)).tw. 4 (body weigh$ or bodyweigh$ or body mass$ or bodymass or body fat$ or bodyfat$).tw. 5 (overeat$ or over‐eat$ or overnourish$ or over‐nourish$ or overnutrit$ or over‐nutrit$).tw. 6 or/1‐5 7 (child$ or schoolchild$ or preschool$ or pre‐school$ or schoolage$ or school‐age$ or schoolboy$ or schoolgirl$ or boy$ or girl$ or preteen$ or teen$ or adolescen$ or youth$ or young people or young person$ or pediatr$ or paediatr$).tw. 8 6 and 7 9 sport$.tw. (8 10 (physical adj3 (activit$ or education$ or exertion$ or training)).tw. 11 exercise$.tw. (2 12 ((diet or dieting) adj5 (health$ or weight$)).tw. 13 (calorie adj3 (control or reduc$ or restriction)).tw. 14 food choice$.tw. 15 (fat camp$ or weight loss camp$).tw. 16 (behavio?r$ adj3 (therap$ or technique$ or modif$ or intervention$)).tw. 17 (cognit$ adj3 (therap$ or technique$ or modif$ or intervention$)).tw. 18 CBT.tw. 19 (psychotherap$ or psycho‐therap$).tw. 20 (family adj3 (therap$ or intervention$)).tw. 21 family‐based.tw. 22 (sedentary adj3 (lifestyle or behavio?r$)).tw. 23 (television or tv).tw. 24 "screen time".tw. 25 ((video or computer) adj game$).tw. 26 (psycho‐social or psychosocial).tw. 27 (lifestyle$ or life‐style$).tw. 28 (health$ adj3 (promot$ or educat$)).tw. 29 (multi‐component$ or multiple component$).tw. 30 or/9‐29 31 8 and 30 32 (Random$ or trial$ or control$ or placebo$ or blind$ or prospectiv$ or meta‐analysis or group or systematic review).tw. 33 31 and 32

#### Embase Ovid

1980 to Week 7 2012, searched 22 February 2012 (3887 records) 1980 to Week 18 2013, searched 7 May 2013. Limited to EM=201209‐21318 (860 records) 1974 to Week 05 2017, searched 3 February 2017, Limited to year: 2013 to current (4255 records)

1     exp Overweight/ 2     Body Weight/ 3     (obes$ or overweight or over‐weight).tw. 4     exp Body Weight Changes/ 5     (weight adj2 (loss or lost or losing or reduc$)).tw. 6     (weight adj2 (gain$ or increas$)).tw. 7     exp body fat distribution/ or body mass index/ or skinfold thickness/ or waist‐hip ratio/ 8     (body weigh$ or bodyweigh$ or body mass$ or bodymass or body fat$ or bodyfat$).tw. 9     Overnutrition/ 10     (overeat$ or over‐eat$ or overnourish$ or over‐nourish$ or overnutrit$ or over‐nutrit$).tw. 11     or/1‐10 12     exp Child/ 13     Adolescent/ 14     (child$ or schoolchild$ or preschool$ or pre‐school$ or schoolage$ or school‐age$ or schoolboy$ or schoolgirl$ or boy$ or girl$ or preteen$ or teen$ or adolescen$ or youth$ or young people or young person$ or pediatr$ or paediatr$).tw. 15     12 or 13 or 14 16     Exercise/ or Exercise Therapy/ 17     Physical Exertion/ 18     Motor Activity/ 19     Sports/ 20     sport$.tw. 21     exp "Physical Education and Training"/ 22     (physical adj3 (activit$ or education$ or exertion$ or training)).tw. 23     exercise$.tw. 24     exp diet therapy/ 25     ((diet or dieting) adj5 (health$ or weight$)).tw. 26     (calorie adj3 (control or reduc$ or restriction)).tw. 27     food choice$.tw. 28     (fat camp$ or weight loss camp$).tw. 29     nutrition education.tw. 30     Nutrition Therapy/ 31     behavior therapy/ 32     Cognitive Therapy/ 33     psychotherapy/ 34     (behavio?r$ adj3 (therap$ or technique$ or modif$ or intervention$)).tw. 35     (cognit$ adj3 (therap$ or technique$ or modif$ or intervention$)).tw. 36     CBT.tw. 37     (psychotherap$ or psycho‐therap$).tw. 38     family therapy/ 39     (family adj3 (therap$ or intervention$)).tw. 40     family‐based.tw. 41     sedentary lifestyle/ (1338) 42     (sedentary adj3 (lifestyle or behavio?r$)).tw. 43     video games/ 44     television/ 45     (television or tv).tw. 46     "screen time".tw. 47     (psycho‐social or psychosocial).tw. 48     exp Health Promotion/ 49     Health Education/ 50     (health$ adj3 (promot$ or educat$ or lifestyle)).tw. 51     lifestyle/ 52     (lifestyle$ or life‐style$).tw. 53     ((video or computer) adj game$).tw. 54     or/16‐53 55     11 and 15 and 54 56     random$.tw. 57     factorial$.tw. 58     crossover$.tw. 59     cross over$.tw. 60     cross‐over$.tw. 61     placebo$.tw. 62     (doubl$ adj blind$).tw. 63     (singl$ adj blind$).tw. 64     assign$.tw. 65     allocat$.tw. 66     volunteer$.tw. 67     Crossover Procedure/ 68     double‐blind procedure.tw. 69     Randomized Controlled Trial/ 70     Single Blind Procedure/ 71     or/56‐70 72     55 and 71 

#### PsycINFO Ovid

1806 to Week 2 February 2012, searched 22 February 2012 (1460 records) 1806 to Week 4 April 2013, searched 7 May 2013, limited to UP=20120218‐20130507 (311 records) 1806 to Week 5 January 2017, searched 3 February 2017, limited to up=20130501‐20170130 (723 records)

1     exp Overweight/ 2     Body Weight/ 3     (obes$ or overweight or over‐weight).tw. 4     (weight adj2 (loss or lost or losing or reduc$)).tw. 5     (weight adj2 (gain$ or increas$)).tw. 6     exp body fat distribution/ or body mass index/ or skinfold thickness/ or waist‐hip ratio/ 7     (body weigh$ or bodyweigh$ or body mass$ or bodymass or body fat$ or bodyfat$).tw. 8     (overeat$ or over‐eat$ or overnourish$ or over‐nourish$ or overnutrit$ or over‐nutrit$).tw. 9     (child$ or schoolchild$ or preschool$ or pre‐school$ or schoolage$ or school‐age$ or schoolboy$ or schoolgirl$ or boy$ or girl$ or preteen$ or teen$ or adolescen$ or youth$ or young people or young person$ or pediatr$ or paediatr$).tw. 10     Exercise/ or Exercise Therapy/ 11     Physical Activity/ 12     Sports/ 13     sport$.tw. 14     exp Physical Education/ 15     (physical adj3 (activit$ or education$ or exertion$ or training)).tw. 16     exercise$.tw. 17     ((diet or dieting) adj5 (health$ or weight$)).tw. 18     (calorie adj3 (control or reduc$ or restriction)).tw. 19     food choice$.tw. 20     (fat camp$ or weight loss camp$).tw. 21     nutrition education.tw. 22     behavior therapy/ 23     Cognitive Therapy/ 24     psychotherapy/ 25     (behavio?r$ adj3 (therap$ or technique$ or modif$ or intervention$)).tw. 26     (cognit$ adj3 (therap$ or technique$ or modif$ or intervention$)).tw. 27     CBT.tw. 28     (psychotherap$ or psycho‐therap$).tw. 29     family therapy/ 30     (family adj3 (therap$ or intervention$)).tw. 31     family‐based.tw. 32     sedentary lifestyle/ 33     (sedentary adj3 (lifestyle or behavio?r$)).tw. 34     video games/ 35     television/ 36     (television or tv).tw. 37     "screen time".tw. 38     (psycho‐social or psychosocial).tw. 39     exp Health Promotion/ 40     Health Education/ 41     (health$ adj3 (promot$ or educat$ or lifestyle)).tw. 42     lifestyle/ 43     (lifestyle$ or life‐style$).tw. 44     ((video or computer) adj game$).tw. 45     or/1‐8 46     or/10‐44 47     9 and 45 and 46 48     Treatment Effectiveness Evaluation/ 49     exp Treatment Outcomes/ 50     Psychotherapeutic Outcomes/ 51     PLACEBO/ 52     exp Followup Studies/ 53     placebo$.tw. 54     random$.tw. 55     comparative stud$.tw. 56     randomi#ed controlled trial$.tw. 57     (clinical adj3 trial$).tw. 58     (research adj3 design).tw. 59     (evaluat$ adj3 stud$).tw. 60     (prospectiv$ adj3 stud$).tw. 61     ((singl$ or doubl$ or trebl$ or tripl$) adj3 (blind$ or mask$)).tw. 62     control$.tw. 63     62 or 54 or 52 or 60 or 59 or 55 or 48 or 53 or 49 or 61 or 57 or 51 or 50 or 58 or 56 64     47 and 63

#### CINAHL Plus EBSCOhost (Cumulative Index to Nursing and Allied Health Literature)

1937 to current, searched 22 February 2012 (1933 records) 1937 to current, searched 7 May 2013, limited to EM=20120222 ‐ current (484 records) 1937 to current, searched 3 February 2017, limited to EM 20130501 ‐ current (2729 records]

S47  (S44 or S45) and (S43 and S46) S46  S44 or S45 S45  (MH "Randomized Controlled Trials") S44  ((random* or blind* or allocat* or assign* or trial* or placebo* or crossover* or cross‐over*)) S43  S9 and S10 and S42 S42  (S11 or S12 or S13 or S14 or S15 or S16 or S17 or S18 or S19 or S20 or S21 or S22 or S23 or S24 or S25 or S26 or S27 or S28 or S29 or S30 or S31 or S32 or S33 or S34 or S35 or S36 or S37 or S38 or S39 or S40 or S41) S41  (((video or computer) N1 game*)) S40  ((lifestyle* or life‐style*)) S39  ((health* N3 (promot* or educat* or lifestyle))) S38  ((psycho‐social or psychosocial)) S37 ("screen time") S36  ((television or tv)) S35 ((sedentary N3 (lifestyle or behavio?r*))) S34  (family‐based) S33 ((family N3 (therap* or intervention*))) S32 ((psychotherap* or psycho‐therap*)) S31 CBT S30 ((cognit* N3 (therap* or technique* or modif* or intervention*))) S29 ((behavio#r* N3 (therap* or technique* or modif* or intervention*))) S28 ("nutrition education") S27 (("fat camp*" or "weight loss camp*")) S26 ("food choice*") S25 ((calorie N3 (control or reduc* or restriction))) S24  (((diet or dieting) N5 (health* or weight*))) S23 (exercise*) S22 ((physical N3 (activit* or education* or exertion* or training))) S21 (sport*) S20 (MH "Health Education") S19 (MH "Health Promotion") S18 (MH "Life Style") S17 (MH "Television") S16 (MH "Video Games") S15 (MH "Family Therapy") S14 (MH "Cognitive Therapy") S13 (MH "Diet Therapy") OR (MH "Behavior Therapy") S12 (MH "Sports") S11 (MH "Exercise") OR (MH "Physical Fitness") S10 ((child* or schoolchild* or preschool* or pre‐school* or schoolage* or school‐age* or schoolboy* or schoolgirl* or boy* or girl* or preteen* or teen* or adolescen* or youth* or young people or young person* or pediatr* or paediatr*)) S9  S1 or S2 or S3 or S4 or S5 or S6 or S7 or S8 S8  ((overeat* or over‐eat* or overnourish* or over‐nourish* or overnutrit* or over‐nutrit*)) S7  (("body weigh*" or bodyweigh* or body mass* or bodymass or "body fat*" or bodyfat*)) S6  ((weight N2 (gain* or increas*))) S5  ((weight N2 (loss or lost or losing or reduc*))) S4 (MH "Hyperphagia") S3  (MH "Weight Loss") S2 (MH "Obesity") S1 ((obes* or overweight or over‐weight)) 

#### ERIC Proquest (Educational Resources Information Centre)

1966 to current, searched 22 February 2012 (1363 records) 1966 to current, searched 8 May 2013, limited to publication year 2012 to 2013 (205 records) 1966 to current, searched 3 February 2017, limited to publication year 2013 to 2017 (223 records)

S1 ((obes* or overweight or over‐weight)) S2 ((weight near/2 (loss or lost or losing or reduc*))) S3 ((weight near/2 (gain* or increas*))) S4 (("body weigh*" or bodyweigh* or body mass* or bodymass or "body fat*" or bodyfat*)) S5 ((overeat* or over‐eat* or overnourish* or over‐nourish* or overnutrit* or over‐nutrit*)) S6 s1 or s2 or s3 or s4 or s5 S7 ((child* or schoolchild* or preschool* or pre‐school* or schoolage* or school‐age* or schoolboy* or schoolgirl* or boy* or girl* or preteen* or teen* or adolescen* or youth* or young people or young person* or pediatr* or paediatr*)) S8 (sport*) S9 ((physical near/3 (activit* or education* or exertion* or training))) S10 (exercise*) S11 (((diet or dieting) near/5 (health* or weight*))) S12 ((calorie near/3 (control or reduc* or restriction))) S13 ("food choice*") S14 (("fat camp*" or "weight loss camp*")) S15 ("nutrition education") S16 ((behavio?r* near/3 (therap* or technique* or modif* or intervention*))) S17 ((cognit* near/3 (therap* or technique* or modif* or intervention*))) S18 (CBT) S19 ((psychotherap* or psycho‐therap*)) S20 ((family near/3 (therap* or intervention*))) S21 (family‐based) S22 ((sedentary near/3 (lifestyle or behavio?r*))) S23 ((television or tv)) S24 ("screen time") S25 ((psycho‐social or psychosocial)) S26 ((health* near/3 (promot* or educat* or lifestyle))) S27 ((lifestyle* or life‐style*)) S28 (((video or computer) near/1 game*)) S29 s8 or s9 or s10 or s11 or s12 or s13 or s14 or s15 or s16 or s17 or s18 or s19 or s20 S30 s21 or s22 or s23 or s24 or s25 or s26 or s27 or s28 S31 s29 or s30 S32 s6 and s7 and s31

#### SPORTDiscus EBSCO

Searched from 1980 to current on 05 March 2012 and 06 May 2013, 6 February 2017, limited to 2013 to current (2186 records)

S66 (S63 and S65) S65 S17 and S57 and S64 S64 S1 or S2 or S3 or S4 or S5 or S6 or S7 or S8 or S9 or S10 or S11 or S12 or S13 S63 S61 NOT S62 S62 SU animals NOT SU humans S61 (S58 or S59 or S60) S60 AB (random* or blind* or allocat* or assign* or trial* or placebo* or crossover or cross‐over) S59 SU controlled clinical trial S58 SU randomized controlled trials S57 (S19 or S20 or S21 or S22 or S23 or S24 or S25 or S26 or S27 or S28 or S29 or S30 or S31 or S32 or S33 or S34 or S35 or S36 or S37 or S38 or S39 or S40 or S41 or S42 or S43 or S44 or S45 or S46 or S47 or S48 or S49 or S50 or S51 or S52 or S53 or S54 or S55 or S56) S56 TX ((computer or video or internet) N1 game) S55 SU computer game S54 TX lifestyle* or life‐style* S53 TX (health* N3 (lifestyle or promotion or education or behavio?r)) S52 SU lifestyle S51 SU Health Education or SU Health Promotion S50 TX psycho‐social or psychosocial S49 TX "screen time" S48 TX television or TV S47 SU video games S46 SU television S45 TX (Sedentary N3 (behavio?r or lifestyle)) S44 SU Sedentary S43 TX family‐based S42 TX (family N3 (therap* or intervention*)) S41 SU family therapy S40 TX psychotherap* or psycho‐therap* Rerun View Details Edit Interface ‐ S39 TX (behavio?r N3 (therap* or technique* or modif* or intervention*)) S38 TX CBT S37 SU Cognitive therapy S36 SU Behavior therapy S35 SU Psychotherapy S34 TX "food choice" S33 TX (calorie N3 (control or reduc* or restriction)) S32 TX ((diet or dieting) N5 (health* or weight*)) S31 TX "fat camp*" or "weight loss camp*" S30 SU food habit S29 SU nutrition therapy S28 SU diet therapy S27 TX exercise* S26 TX sport* S25 TX (Physical N2 (activit* or education* or training or fitness)) S24 SU Physical training S23 SU Physical activity S22 SU Physical education S21 SU Sport S20 SU Exercise Therapy S19 SU Exercise S18 (S14 or S15 or S16 or S17) S17 TX child* or schoolchild* or preschool* or pre‐school* or schoolage* or school‐age* or schoolboy* or schoolgirl* or boy* or girl* or preteen* or teen* or adolescen* or youth* or young people or young person* or pediatr* or paediatr* S16 SU teenager S15 SU adolescent S14 SU child S13 TX Overeat* or over‐eat* or overnourish* or over‐nourish* or overnutrit* or over‐nutrit* S12 TX "waist‐hip ratio" S11 TX "body weigh*" or bodyweigh* or body mass* or bodymass or "body fat*" or bodyfat* S10 TX waist‐hip ration S9 TX skin fold thickness S8 TX body fat distribution S7 SU body composition S6 TX (weight N2 (gain* or increas*)) S5 TX (weight N2 (loss or lost or losing or reduc*)) S4 TX obes* or overweight or over‐weight S3 SU body weight change S2 SU body weight S1 SU overweight

#### IBSS (International Bibliography of Social Studies) Proquest

1951 to current, searched 22 February 2012 (459 records) 1951 to current, searched 8 May 2013, limited to publication year 2012 to 2013 (113 records) 1951 to current searched 3 Feburary 2017, limited to publication year 2013 to 2017 (200 records)

S1 ((obes* or overweight or over‐weight)) S2 ((weight near/2 (loss or lost or losing or reduc*))) S3 ((weight near/2 (gain* or increas*))) S4 (("body weigh*" or bodyweigh* or body mass* or bodymass or "body fat*" or bodyfat*)) S5 ((overeat* or over‐eat* or overnourish* or over‐nourish* or overnutrit* or over‐nutrit*)) S6 s1 or s2 or s3 or s4 or s5 S7 ((child* or schoolchild* or preschool* or pre‐school* or schoolage* or school‐age* or schoolboy* or schoolgirl* or boy* or girl* or preteen* or teen* or adolescen* or youth* or young people or young person* or pediatr* or paediatr*)) S8 (sport*) S9 ((physical near/3 (activit* or education* or exertion* or training))) S10 (exercise*) S11 (((diet or dieting) near/5 (health* or weight*))) S12 ((calorie near/3 (control or reduc* or restriction))) S13 ("food choice*") S14 (("fat camp*" or "weight loss camp*")) S15 ("nutrition education") S16 ((behavio?r* near/3 (therap* or technique* or modif* or intervention*))) S17 ((cognit* near/3 (therap* or technique* or modif* or intervention*))) S18 (CBT) S19 ((psychotherap* or psycho‐therap*)) S20 ((family near/3 (therap* or intervention*))) S21 (family‐based) S22 ((sedentary near/3 (lifestyle or behavio?r*))) S23 ((television or tv)) S24 ("screen time") S25 ((psycho‐social or psychosocial)) S26 ((health* near/3 (promot* or educat* or lifestyle))) S27 ((lifestyle* or life‐style*)) S28 (((video or computer) near/1 game*)) S29 s8 or s9 or s10 or s11 or s12 or s13 or s14 or s15 or s16 or s17 or s18 or s19 or s20 S30 s21 or s22 or s23 or s24 or s25 or s26 or s27 or s28 S31 s29 or s30 S32 s6 and s7 and s31

#### Conference Proceeding Citation Index–Science (CPCI‐S) and Conference Proceeding Citation Index–Social Sciences & Humanities (CPCI‐SS&H) Web of Science (Clarivate)

1990 to 17 February 2012, searched 22 February 2012 (871 records) 1990 to 3 May 2013, searched 8 May 2013 (12 records) 1990 to 2 February 2017, searched 3 February 2017, limited to 2013 to current (35 records)

#32 #31 AND #30 #31 Topic=((random* or blind* or allocat* or assign* or trial* or placebo* or crossover* or cross‐over*)) #30 #29 AND #7 #29 #28 OR #27 OR #26 OR #25 OR #24 OR #23 OR #22 OR #21 OR #20 OR #19 OR #18 OR #17 OR #16 OR #15 OR #14 OR #13 OR #12 OR #11 OR #10 OR #9 OR #8 #28 Topic=(((video or computer) near/1 game*)) #27 Topic=((lifestyle* or life‐style*)) #26 Topic=((health* near/3 (promot* or educat* or lifestyle))) #25 Topic=((psycho‐social or psychosocial)) #24 Topic=("screen time") #23 Topic=((television or tv)) #22 Topic=((sedentary near/3 (lifestyle or behavio?r*))) #21 Topic=(family‐based) #20 Topic=((family near/3 (therap* or intervention*))) #19 Topic=((psychotherap* or psycho‐therap*)) #18 Topic=(CBT) #17 Topic=((cognit* near/3 (therap* or technique* or modif* or intervention*))) #16 Topic=((behavio?r* near/3 (therap* or technique* or modif* or intervention*))) #15 Topic=("nutrition education") #14 Topic=(("fat camp*" or "weight loss camp*")) #13 Topic=("food choice*") #12 Topic=((calorie near/3 (control or reduc* or restriction))) #11 Topic=(((diet or dieting) near/5 (health* or weight*))) #10 Topic=(exercise*) #9 Topic=((physical near/3 (activit* or education* or exertion* or training))) #8 Topic=(sport*) #7 Topic=((child* or schoolchild* or preschool* or pre‐school* or schoolage* or school‐age* or schoolboy* or schoolgirl* or boy* or girl* or preteen* or teen* or adolescen* or youth* or young people or young person* or pediatr* or paediatr*)) #6 #5 OR #4 OR #3 OR #2 OR #1 #5 Topic=((overeat* or over‐eat* or overnourish* or over‐nourish* or overnutrit* or over‐nutrit*)) #4 Topic=(("body weigh*" or bodyweigh* or body mass* or bodymass or "body fat*" or bodyfat*)) #3 Topic=((weight near/2 (gain* or increas*))) #2 Topic=((weight near/2 (loss or lost or losing or reduc*))) #1 Topic=((obes* or overweight or over‐weight))

#### Cochrane Database of Systematic Reviews (CDSR) part of the Cochrane Library

2012 (Issue 12), searched 15 January 2012 (22 records) 2013 (Issue 4), searched 8 May 2013, limited to publication year 2012 to 2013 (11 records) 2017 (Issue 1), searched 2 February 2017, limited to online publications date from May 2013 to Jan 2017 (32 records)

#1MeSH descriptor: [Overweight] explode all trees #2MeSH descriptor: [Body Weight] this term only #3(obese or obesity or overweight or over‐weight):ti,ab #4MeSH descriptor: [Body Weight Changes] explode all trees #5(weight near/2 (loss or lost or losing or reduc*)):ti,ab #6(weight near/2 (gain* or increas*)):ti,ab #7MeSH descriptor: [Body Fat Distribution] explode all trees #8MeSH descriptor: [Body Mass Index] explode all trees #9MeSH descriptor: [Skinfold Thickness] explode all trees #10MeSH descriptor: [Waist‐Hip Ratio] explode all trees #11("body weigh*" or bodyweigh* or "body mass*" or bodymass or "body fat*" or bodyfat*):ti,ab #12MeSH descriptor: [Overnutrition] this term only #13(overeat* or over‐eat* or overnourish* or over‐nourish* or overnutrit* or over‐nutrit*):ti,ab #14#1 or #2 or #3 or #4 or #5 or #6 or #7 or #8 or #9 or #10 #11 or #12 or #13 #15MeSH descriptor: [Child] explode all trees #16MeSH descriptor: [Adolescent] this term only #17(child* or schoolchild* or preschool* or pre‐school* or schoolage* or school‐age* or schoolboy* or schoolgirl* or boy* or girl* or preteen* or teen* or adolescen* or youth* or "young people" or "young person*" or pediatr* or paediatr*):ti,ab #18#15 or #16 or #17 #19#14 and #18 #20MeSH descriptor: [Exercise] this term only #21MeSH descriptor: [Exercise Therapy] this term only #22MeSH descriptor: [Physical Exertion] this term only #23MeSH descriptor: [Motor Activity] this term only #24MeSH descriptor: [Sports] this term only #25(sport*):ti,ab #26MeSH descriptor: [Physical Education and Training] explode all trees #27(physical near/3 (activit* or education* or exertion* or training)):ti,ab #28(exercise*):ti,ab #29MeSH descriptor: [Diet Therapy] explode all trees #30((diet or dieting) near/5 (health* or weight*)):ti,ab #31(calorie near/3 (control or reduc* or restriction)):ti,ab #32("food choice*"):ti,ab #33("fat camp*" or "weight loss camp*"):ti,ab #34("nutrition education") ti,ab #35MeSH descriptor: [Nutrition Therapy] this term only #36MeSH descriptor: [Behavior Therapy] this term only #37MeSH descriptor: [Cognitive Therapy] this term only #38MeSH descriptor: [Psychotherapy] this term only #39((behavior* or behavior*) near/3 (therap* or technique* or modif* or intervention*)):ti,ab #40(cognit* near/3 (therap* or technique*or modif* or intervention*)):ti,ab #41(CBT) ti,ab #42(psychotherap* or psycho‐therap*) ti,ab #43MeSH descriptor: [Family Therapy] this term only #44(family near/3 (therap* or intervention*)):ti,ab #45(family‐based):ti,ab #46MeSH descriptor: [Sedentary Lifestyle] this term only #47(sedentary near/3 (lifestyle or behavio*r*)):ti,ab #48MeSH descriptor: [Video Games] this term only #49MeSH descriptor: [Television] this term only #50(television or tv):ti,ab #51("screen time"):ti,ab #52(psycho‐social or psychosocial):ti,ab #53MeSH descriptor: [Health Promotion] explode all trees #54MeSH descriptor: [Health Education] this term only #55(health* near/3 (promot* or educat* or lifestyle)):ti,ab #56MeSH descriptor: [Life Style] this term only #57(lifestyle* or life‐style*):ti,ab #58((video or computer) next game*):ti,ab #59#20 or #21 or #22 or #23 or #24 or #25 or #26 or #27 or #28 or #29 or #30 or #31 or #32 or #33 or #34 or #35 or #36 or #37 or #38 or #39 or #40 or #41 or #42 or #43 or #44 or #45 or #46 or #47 or #48 or #49 or #50 or #51 or #52 or #53 or #54 or #55 or #56 or #57 or #58 #60#19 and #59

#### Database of Abstracts of Reviews of Effects (DARE) part of the Cochrane Library

2012 (4), searched 15 January 2013 (8 records) 2013 (2), searched 8 May 2013, limited to publication year 2012 to 2013 (16 records) 2015 (2), searched 2 February 2017 (0 records)

#1MeSH descriptor: [Overweight] explode all trees #2MeSH descriptor: [Body Weight] this term only #3(obese or obesity or overweight or over‐weight):ti,ab #4MeSH descriptor: [Body Weight Changes] explode all trees #5(weight near/2 (loss or lost or losing or reduc*)):ti,ab #6(weight near/2 (gain* or increas*)):ti,ab #7MeSH descriptor: [Body Fat Distribution] explode all trees #8MeSH descriptor: [Body Mass Index] explode all trees #9MeSH descriptor: [Skinfold Thickness] explode all trees #10MeSH descriptor: [Waist‐Hip Ratio] explode all trees #11("body weigh*" or bodyweigh* or "body mass*" or bodymass or "body fat*" or bodyfat*):ti,ab #12MeSH descriptor: [Overnutrition] this term only #13(overeat* or over‐eat* or overnourish* or over‐nourish* or overnutrit* or over‐nutrit*):ti,ab #14#1 or #2 or #3 or #4 or #5 or #6 or #7 or #8 or #9 or #10 #11 or #12 or #13 #15MeSH descriptor: [Child] explode all trees #16MeSH descriptor: [Adolescent] this term only #17(child* or schoolchild* or preschool* or pre‐school* or schoolage* or school‐age* or schoolboy* or schoolgirl* or boy* or girl* or preteen* or teen* or adolescen* or youth* or "young people" or "young person*" or pediatr* or paediatr*):ti,ab #18#15 or #16 or #17 #19#14 and #18 #20MeSH descriptor: [Exercise] this term only #21MeSH descriptor: [Exercise Therapy] this term only #22MeSH descriptor: [Physical Exertion] this term only #23MeSH descriptor: [Motor Activity] this term only #24MeSH descriptor: [Sports] this term only #25(sport*):ti,ab #26MeSH descriptor: [Physical Education and Training] explode all trees #27(physical near/3 (activit* or education* or exertion* or training)):ti,ab #28(exercise*):ti,ab #29MeSH descriptor: [Diet Therapy] explode all trees #30((diet or dieting) near/5 (health* or weight*)):ti,ab #31(calorie near/3 (control or reduc* or restriction)):ti,ab #32("food choice*"):ti,ab #33("fat camp*" or "weight loss camp*"):ti,ab #34("nutrition education") ti,ab #35MeSH descriptor: [Nutrition Therapy] this term only #36MeSH descriptor: [Behavior Therapy] this term only #37MeSH descriptor: [Cognitive Therapy] this term only #38MeSH descriptor: [Psychotherapy] this term only #39((behavior* or behavior*) near/3 (therap* or technique* or modif* or intervention*)):ti,ab #40(cognit* near/3 (therap* or technique*or modif* or intervention*)):ti,ab #41(CBT) ti,ab #42(psychotherap* or psycho‐therap*) ti,ab #43MeSH descriptor: [Family Therapy] this term only #44(family near/3 (therap* or intervention*)):ti,ab #45(family‐based):ti,ab #46MeSH descriptor: [Sedentary Lifestyle] this term only #47(sedentary near/3 (lifestyle or behavio*r*)):ti,ab #48MeSH descriptor: [Video Games] this term only #49MeSH descriptor: [Television] this term only #50(television or tv):ti,ab #51("screen time"):ti,ab #52(psycho‐social or psychosocial):ti,ab #53MeSH descriptor: [Health Promotion] explode all trees #54MeSH descriptor: [Health Education] this term only #55(health* near/3 (promot* or educat* or lifestyle)):ti,ab #56MeSH descriptor: [Life Style] this term only #57(lifestyle* or life‐style*):ti,ab #58((video or computer) next game*):ti,ab #59#20 or #21 or #22 or #23 or #24 or #25 or #26 or #27 or #28 or #29 or #30 or #31 or #32 or #33 or #34 or #35 or #36 or #37 or #38 or #39 or #40 or #41 or #42 or #43 or #44 or #45 or #46 or #47 or #48 or #49 or #50 or #51 or #52 or #53 or #54 or #55 or #56 or #57 or #58 #60#19 and #59

#### DoPHER (Database of Promoting Health Effectiveness Reviews)

(www.eppi.ioe.ac.uk/webdatabases4/SearchIntro.aspx)

Searched 23 February 2012, 06 May 2013 and 06 February 2017 (113 records)

(Child* OR adolesc* OR youth OR boy* OR girl* OR paediatr* OR pediatr*) AND (obes* OR overweight OR BMI OR “body mass index” OR “body weight change”)

#### Bibliomap

(eppi.ioe.ac.uk/webdatabases/SearchIntro.aspx)

Searched 23 February 2012, 06 May 2013 and 06 February 2017 (0 records)

(child* OR adolesc* OR youth OR boy* OR girl* OR paediatr* OR pediatr*)) AND (obes* OR overweight)

#### Trials Register of Promoting Health Interventions (TRoPHI)

(eppi.ioe.ac.uk/webdatabases4/SearchIntro.aspx)

Searched 23 February 2012, 06 May 2013 and 06 February 2017 (255 records)

(child* OR adolesc* OR youth OR boy* OR girl* OR paediatr* OR pediatr*) AND (obes* OR overweight)

#### Dissertations and Theses Global (Proquest)

2012 to 2017, searched 8 February 2017, limited to publication year 2013 to 2017 (24 records)

(ab(weight NEAR/2 (gain* OR increas*)) OR ab(obes* or overweight or over‐weight) OR ab(weight NEAR/2 (loss OR lost OR losing OR reduc*)) OR ab(("body weigh*" OR bodyweigh* OR body mass* OR bodyarts OR "body fat*" OR bodyfat*)) OR su(obesity) OR su(overweight))

AND

(su(child) OR ab(child* OR schoolchild* OR preschool* OR pre‐school* OR schoolage* OR school‐age* OR schoolboy* OR schoolgirl* OR boy* OR girl* OR preteen* OR teen* OR adolescen* OR youth* OR young people OR young person* OR pediatr* OR paediatr*))

AND

(ab(physical NEAR/3 (activit* OR education* OR exertion* OR training)) OR ab(exercis* OR sport*) OR ab((diet OR dieting) NEAR/5 (health* OR weight*)) OR ab(calorie NEAR/3 (control OR reduc* OR restriction)) OR ab("fat camp*" OR "weight loss camp*") OR ab("nutrition education") OR su(nutrition education) OR ab(behavio?r* NEAR/3 (therap* OR technique* OR modif* OR intervention*)) OR ab(cognit* NEAR/3 (therap* OR technique* OR modif* OR intervention*)) OR ab(psychotherap* OR psycho‐therap*) OR ab(family near/3 (therap* or intervention*)) OR ab(sedentary NEAR/3 (lifestyle OR behavio?r*)) OR ab(“screen time”) OR ab(health* near/3 (promot* or educat* or lifestyle)) OR ab(lifestyle* or life‐style*))

AND

(ab("random* controlled trial*") OR ab(random* controlled trial*) OR su(randomized controlled trial))

#### ISRCTN Registry

(www.isrctn.com)

2001 to current, searched 08 February 2017 (67 records)

Obes* child* Obes* youth Obes* adolesc* Overweight child*

#### World Health Organization International Clinical Trials Registry Platform (WHO ICTRP)

(www.who.int/trialsearch)

searched 27 February 2012, 06 May 2013, and 08 February 2017 limited to 07/05/2013 ‐ 31/01/2017 (600 records)

Condition: (obes% or overweight) restricted to “Search for clinical trials in children” option

#### Database on Obesity and Sedentary Behaviour Studies

Searched 23 February 2012 and 06 May 2013

Child* OR adolesc* OR youth OR boy* Or girl* Or paediatr* OR pediatr*

#### MIT Cognet

Searched 23 February 2012 and 06 May 2013

(child* OR adolesc*) AND (obes* OR overweight)

#### Networked Digital Library of Theses and Dissertations

Searched 28 February 2012 and 06 May 2013

(children OR adolescents OR youth) and (overweight OR obesity) AND (randomised controlled trial)

#### OpenSIGLE (Open Grey)

searched 23 February 2012 and 06 May 2013

(child* OR adolesc* OR youth or boy* or girl*) AND (obes* OR overweight)

### Appendix 2. Additional methods

**Table:**

**Method item**	**Additional methods**
**Measures of treatment effect**	**Dichotomous data** Dichotomous outcomes will be summarised as a risk ratio (RR) with a 95% confidence interval (CI). Using the risk ratio rather than the odds ratio minimises misinterpretation of the occurrence of the treatment effect and avoids subsequent conversion of odds ratios to risk ratios for correct interpretation. In the ‘Summary of findings’ table, we will express dichotomous data as relative (risk ratio) and absolute (number of children per 1000) risk. **Ordinal data** For ordinal data, we will analyse longer ordinal scales (e.g. Wechsler Intelligence Scale for Children) as continuous data (Section 9.2.4. in Higgins 2011). When studies use short ordinal scales (e.g. A to F classification of educational achievement), we will convert these to dichotomous data by combining adjacent categories and calculating the risk ratio (Section 9.2.4. in Higgins 2011). Dichotomisation will be done according to the cut‐offs considered as ‘pass’ or ‘fail’
**Unit of analysis issues**	**Multiple time points** We will analyse data from studies that reported results at more than one time point in a separate meta‐analysis with comparable data from other studies at similar time points. We will group post‐intervention time points as immediately after intervention, one to five months, six to 11 months, 12 to 23 months and ≥ 24 months after intervention
**Assessment of reporting biases**	We will assess reporting bias by using a funnel plot to evaluate the association between effect size and standard error, if a sufficient number of studies (at least 10 studies) are included in a meta‐analysis. An asymmetrical plot may indicate publication bias or a real relationship between study size and effect size, as when larger trials have lower compliance rates and compliance is positively related to effect size. If we find such a relationship, we will explore clinical variation as a possible explanation. When the number of included studies is low, an asymmetrical funnel plot may be due to heterogeneity in the intervention effect or chance
**Synthesis of continuous and dichotomous data**	If similar outcome data are extracted as both dichotomous and continuous measures (e.g. exam results expressed as pass or fail or as a percentage score), we will used the inverse variance method to combine data; to do this, we will convert the risk ratio to lnRR and standard error (SE) of lnRR for entry into Review Manager 5
**Subgroup analysis and investigation of heterogeneity**	We will conduct subgroup analyses on the following: Participant characteristics Age (preschool vs primary or elementary school vs secondary or high school) Gender (male vs female) Weight status (overweight vs obesity) Location (low‐ and middle‐income countries vs high‐income countries) Study design characteristics Setting (home vs clinic vs school vs community) Intervention duration (< six months vs ≥ six months) Type of intervention (single component vs multicomponent; energy balance intervention vs behavioural intervention) Type of outcome assessment (formal educational assessment vs non‐formal assessment (e.g. research‐only data)) These subgroups are exploratory because they are based on non‐experimental conditions (cross‐sectional studies); large numbers of subgroup analyses may lead to misleading conclusions (Oxman 1992; Yusuf 1991). We will therefore treat any conclusions with caution when performing subgroup analyses.

### Appendix 3. Intercluster correlation coefficients used for estimating the effective sample size in cluster RCTs of primary outcomes

**Table:**

**Study ID**	**ICC**	**95% CI**	**Analysis method**	**Source**	**Intervention type**
**Grade‐Point Average**					
**Ahamed 2007**	0.18	0.11 to 0.27	One‐way ANOVA, baseline data	Re‐analysed from raw data	PA + Education
**Nanney 2016**	0.05	0.02 to 0.09	One‐way ANOVA, baseline data	Re‐analysed from raw data	Diet + Education
**Mathematics Achievement**					
**Ahamed 2007**	0.10	0.04 to 0.18	One‐way ANOVA, baseline data	Re‐analysed from raw data	PA + Education
**Barbosa Filho 2017 [pers comm]**	0.03	0.00 to 0.13	One‐way ANOVA, baseline data	Trial authors	PA + Education
**Damsgaard 2017 [pers comm]**	0.05	‐	‐	Nanney 2016	Diet + Education
**Sánchez‐López 2017 [pers comm]**	0.31	0.08 to 0.65	Mixed effects models using baseline data adjusted by age, sex and socioeconomic level	Trial authors	PA only
**Treu 2017**	0.05	Not reported	Not reported	Trial authors	PA + Education
**Reading Achievement**					
**Ahamed 2007**	0.10	0.04 to 0.18	One‐way ANOVA, baseline data	Re‐analysed from raw data	PA + Education
**Damsgaard 2017 [pers comm]**	0.05	‐	‐	Nanney 2016	Diet + Education
**Treu 2017**	0.05	Not reported	Not reported	Trial authors	PA + Education
**Language Achievement**					
**Ahamed 2007**	0.25	0.17 to 0.35	One‐way ANOVA, baseline data	Re‐analysed from raw data	PA + Education
**Barbosa Filho 2017 [pers comm]**	0.01	0.00 to 0.07	One‐way ANOVA, baseline data	Trial authors	PA + Education
**Sánchez‐López 2017 [pers comm]**	0.54	0.27 to 0.79	Mixed effects models using baseline data adjusted by age, sex and socioeconomic level	Trial authors	PA only
**Winter 2011**	0.01	Not reported	Not reported	Report article	PA + Education
**Inhibition control**					
**Wirt 2013 [pers comm]**	0.03	‐	‐	Wright 2016	PA + Education
**Visuo‐spatial abilities**					
**Sánchez‐López 2017 [pers comm]**	0.32	0.1 to 0.64	Mixed effects models using baseline data adjusted by age, sex and socioeconomic level	Trial authors	PA only
**Attention**					
**Damsgaard 2017 [pers comm]**	0.05	‐	‐	Nanney 2016	Diet + Education
**Gallotta 2015**	0.03	‐	‐	Wright 2016	PA + Education
**Wirt 2013 [pers comm]**	0.03	‐	‐	Wright 2016	PA + Education
**General Intelligence**					
**Sánchez‐López 2017 [pers comm]**	0.44	0.18 to 0.73	Mixed effects models using baseline data adjusted by age, sex and socioeconomic level	Trial authors	PA only

**ICC**: Intracluster correlation coefficient, **CI**: Confidence interval, **ANOVA**: Analysis of variance, **PA**: Physical Activity

Note: De Greeff 2016 and Resaland 2016 corrected the sample size for cluster randomisation a priori using an ICC of 0.10 (De Greeff 2016) and an ICC of 0.15 (Resaland 2016). The ICC used in Melnyk 2013 was not obtainable from the trial authors. The report states "A number of simulations were run to assess power for the omnibus ANOVA test and the a priori comparison of between group differences at each time point, varying both the class size and the intraclass correlation coefficient" (page 410). Johnston 2013 provided outcome data calculated using generalised linear models, which accounted for the clustered nature of the data (i.e. students nested within schools).

### Appendix 4. Summary of school achievement and cognitive function measures and test tools used in included studies

**Table:**

**Outcomes**	**Tests**	**Cognitive processes**		**Standardised score/scale range**	**Units**		**Scale direction**
**SCHOOL ACHIEVEMENT**							
**Mathematics**	CAT‐3	Number concepts, measurement, patterns, data analysis and probability, geometry and spatial sense		M = 500, SD = 70	Number of correct answers		High = better performance
	W‐J Tests of Achievement III (broad math)	Simple and complex calculation skills, math fluency (number facility*)*, mathematical reasoning and problem analysis and solving		M = 100, SD = 15 (range zero to 200) ≥ 131 = very superior; 121 to 130 = superior; 111 to 120 = high average; 90 to 110 = average; 80 to 89 = low average; 70 to 79 = low; ≤ 69 = very low	Number of correct responses		High = better performance
	Standardised Norwegian national tests	Not reported		M = 50, SD = 10	Number of correct responses		High = better performance
	BADyG‐I (Numerical quantitative concepts)	Numerical reasoning, number comprehension		Scale range: 0 to 36	Number of correct responses		High = better performance
	MCAP	Mathematics problem‐solving skills		Not obtainable	Number of correct responses		High = better performance
	Danish standard test	Mathematics problem‐solving skills. The tests are diagnostic tests designed to measure math skills relative to the grade level		Scale range 3rd grade: 0 to 50 Scale range 4th grade: 0 to 69	Number of correctly solved problems		High = better performance
**Native Language**	Standardised Brazilian National Test (Portuguese)	‐		Scale range = 0 to 10	‐		‐
	Danish standard tests for mathematics proficiency	Mathematics problem solving		Not reported	Number of correctly solved problems		High = better performance
	CAT‐3 (English)	Sentence structure, writing conventions, paragraph structure, information management		M = 500, SD = 70	Number of correct responses		High = better performance
	PPVT III (English)	Receptive vocabulary acquisition		M =100, SD = 15	Number of correct responses		High = better performance
	BADyG‐I Analogical relations and Complex verbal orders scale (Spanish)	Measures the ability to discover relationships between concepts and verbal comprehension.		Scale range: 0 to 36	Number of correct responses		High = better performance
	Standardised Brazilian National Test (Portuguese)	Not obtainable		Scale range = 0 to 10	Number of correct responses		High = better performance
**Second Language**	Standardised Norwegian national tests in English	Not obtainable		Mean = 50, SD = 10	Number of correct responses		High = better performance
**Reading**	CAT‐3	Reading decoding (letter‐word identification), words/phrases in context, reading comprehension (stated information, visual materials, central thought), analysis of text, critical assessment		M = 500, SD = 70	Number of correct responses		High = better performance
	W‐J Tests of Achievement III (broad reading)	Reading decoding (letter‐word identification), reading fluency (speed), reading comprehension of textual information		M = 100, SD = 15 (range zero to 200) ≥ 131 = very superior; 121 to 130 = superior; 111 to 120 = high average; 90 to 110 = average; 80 to 89 = low average; 70 to 79 = low; ≤ 69 = very low	Number of correct responses		High = better performance
	AIMSweb standardised test: Reading–Curriculum‐Based Measurement	Reading fluency		Not obtainable	Number of correct responses		High = better performance
	Standardised Norwegian national test	Not reported		Mean = 50, SD = 10	Number of correct responses		High = better performance
	The Sentence Reading Test 2 (Danish standard test)	Test performance draws on the working memory of the child and reflects the reading comprehension of the child, which includes accurate and fluent decoding of words, vocabulary knowledge, and thinking and reasoning skills. The sentences gradually become longer and more complicated, and as complexity increases, thoughtful analysis of content becomes more essential to comprehension in order to solve the task, e.g. the ability to make inferences		Scale range: 0 to 108	Number of correct responses (relates to the reading proficiency) Total number of sentences read (reflects the reading speed)		High = better performance
**Health Class**	Health Education tests	Course content included choosing and financing health services; communicable diseases; chronic disorders; abuse of drugs, alcohol, and tobacco, exercise, accidents, immunisation, nutrition and body care		Scale range: 0 to 4	not reported		High = better performance
**COGNITIVE FUNCTION**							
**Composite executive functions**	D–KEFS (Design Fluency and Trail‐Making)		Subscales measure visual–spatial skills, response inhibition, motor planning, visual scanning, speed and cognitive flexibility	M = 10, SD = 3	Number of correct responses		High = better performance
	CAS (Planning Scale)		Composite of scores for matching numbers, planned codes and planned connections tests. Strategy generation and application, self‐regulation, intentionality and utilisation of knowledge	M = 100, SD = 15	Sum of total time scale score and accuracy scale score (ratio of number of correct responses and total time)		High = better performance
**Inhibition control**	KiTAP (Go/No Go Task)		Impulsivity	M = 50, SD = 10	Number of errors minus reaction time		Low = better performance
	Stroop test (colour and words): Golden method		Inhibition	Scale range: 0 to100	Interference score: score for congruent condition minus incongruent condition.		Higher = better performance
**Memory**	Rey Complex Figure Test (immediate recall trial)		Accuracy of reproducing a visual pattern following a 3‐minute delay.	Scale range: 0 to 36	Number of correctly reproduced elements		Higher = better performance
	CAS (successive processing scale)		Composite of word series, sentence repetition, and sentence questions tasks. Remembering or completing information in a specific order or sequence	M = 100, SD = 15	Number of correct responses scale score and total time scale score		High = better performance
**Working memory**	Digit span backward		Verbal working memory task	Scale range: 0 to 21	Correctly recalled sequences		High = better performance
	Visual Span Backward test		Non‐verbal working memory task	Scale range: 0 to 12	Correctly tapped sequences		High = better performance
**Visuo‐spatial abilities**	CAS (Simultaneous processing^a^ scale)		Composite of nonverbal matrices, verbal‐spatial relations and figure memory tasks. Nonverbal and verbal processing, analyses and synthesis of logical and grammatical components of language and comprehension of word relationships, nonverbal matrices, verbal spatial relations and figure memory	M = 100, SD = 15	Scale score of number of correct responses		High = better performance
	Rey Complex Figure Test (Copy trial)		Accuracy of processing and reproducing a visual pattern	Scale range: 0 to 36	Number of correctly reproduced elements	Higher = better	
	BADyG‐I		Non‐verbal logical puzzle figures	Scale range: 0 to 36	Number of correct responses	Higher = better	
**Cognitive flexibility**	Modified version WCST		Set shifting	Not reported	Categorising efficiency score: for every correctly sorted rule 6 points were awarded and 1 point for each of the 48 cards not used.	Higher = better performance	
	WCST Computer Version 4 ‐Research Edition		Set shifting	Not reported	Total number of errors	Lower = better performance	
**Attention**	CAS^b^ (attention scale)		Composite of expressive attention, number detection and receptive attention tasks. Requires sustained, selective and focused attention including inhibiting responses	M = 100, SD = 15	Sum of scale scores of accuracy 1 and accuracy 2; accuracy 1 (ratio of number of correct responses and total time); accuracy 2 (ratio of (number of correct responses minus number of false detections) and total time)	High = better performance	
	d2‐R test of attention		The test determines the capacity to focus on 1 stimulus/fact, while suppressing awareness of competing distractors. Selective attention was also required. The performance on this test reflects visual perceptual speed and concentration capacities	Not reported	The total number of items processed (processing speed); Number of letters correctly marked minus errors of commission (concentration performance)	Higher = better	
	d2 test of attention		Involves mental concentration, visual perception, visual scanning ability and perceptual speed	‐359 to 299	Processed characters (defined as the number of correctly marked target characters minus errors of commission (incorrectly marked distractor characters) = concentration performance	Higher = better	
	KiTAP		Sustained attention including aspects of working memory and mental flexibility	M = 50, SD = 10 (range zero to 100)	Number of correct responses based on the difference in maximal numbers of possible errors and omissions	High = better performance	
**General intelligence**	BADyG‐I		Composite of non‐verbal tests (e.g. reasoning and logical puzzle figures), verbal tests (e.g. numerical quantitative concepts) and additional tests (e.g. auditory perception)	Scale range: 0 to 108	Number of correct responses	High = better performance	

CAT‐3: Canadian Achievement Test, version 3; W‐J: Woodcock‐Johnson; MCAT ‐ Mathematics Concepts and Applications Test, M‐CBM: Mathematics–Curriculum‐Based Measurement, PPVT III: Peabody Picture Vocabulary Test, version 3; CAS: Das‐Naglieri‐Cognitive Assessment System; KiTAP: [Kinderversion der Testbatterie zur Aufmerksamkeitsprüfung] Attention test battery for children; D–KEFS: Delis‐Kaplan Executive Function System. BADyG‐I: [Batería de aptitudes diferenciales y generals] Differential Aptitude Battery‐ General scale, WCST: Wisconsin card sorting test. ^a^Simultaneous processing includes tests of memory and executive function. ^b^CAS also includes measures that could be categorised as speed or executive function.

## Data and analyses

**Comparison 1:** Physical activity intervention versus standard practice

Outcome or subgroup title	No. of studies	No. of participants	Statistical method	Effect size
1 Mathematics achievement	3		Std. Mean Difference (IV, Random, 95% CI)	Subtotals only
1.1 Change from baseline	2	255	Std. Mean Difference (IV, Random, 95% CI)	0.49 [‐0.04, 1.01]
1.2 Endpoint	2	314	Std. Mean Difference (IV, Random, 95% CI)	0.19 [‐0.03, 0.42]
2 Reading achievement	2	308	Std. Mean Difference (IV, Random, 95% CI)	0.10 [‐0.30, 0.49]
3 Language achievement	1		Mean Difference (IV, Random, 95% CI)	Totals not selected
4 2nd Language achievement	1		Mean Difference (IV, Random, 95% CI)	Totals not selected
5 Composite executive functions	2		Mean Difference (IV, Random, 95% CI)	Subtotals only
5.1 Change from baseline	1	54	Mean Difference (IV, Random, 95% CI)	8.45 [‐1.67, 18.56]
5.2 Endpoint	1	116	Mean Difference (IV, Random, 95% CI)	5.0 [0.68, 9.32]
6 Inhibition control	1		Mean Difference (IV, Random, 95% CI)	Totals not selected
7 Attention	2	157	Std. Mean Difference (IV, Random, 95% CI)	0.46 [‐0.16, 1.08]
8 Verbal working memory	1		Mean Difference (IV, Random, 95% CI)	Totals not selected
9 Non‐verbal working memory	1		Mean Difference (IV, Random, 95% CI)	Totals not selected
10 Visuo‐spatial abilities	2		Mean Difference (IV, Random, 95% CI)	Totals not selected
10.1 Change from baseline	1		Mean Difference (IV, Random, 95% CI)	0.0 [0.0, 0.0]
10.2 Endpoint	1		Mean Difference (IV, Random, 95% CI)	0.0 [0.0, 0.0]
11 Cognitive flexibility	2	162	Std. Mean Difference (IV, Random, 95% CI)	‐0.06 [‐0.37, 0.25]
12 Non‐verbal memory	1		Mean Difference (IV, Random, 95% CI)	Totals not selected
13 General intelligence	1		Mean Difference (IV, Random, 95% CI)	Totals not selected

**Comparison 2:** Physical activity plus healthy lifestyle education interventions versus standard practice

Outcome or subgroup title	No. of studies	No. of participants	Statistical method	Effect size
1 Average school achievement	1		Mean Difference (IV, Random, 95% CI)	Totals not selected
2 Mathematics achievement	3	384	Std. Mean Difference (IV, Random, 95% CI)	0.02 [‐0.19, 0.22]
3 Reading achievement	2	284	Std. Mean Difference (IV, Random, 95% CI)	‐0.00 [‐0.24, 0.24]
4 Language achievement	3	244	Std. Mean Difference (IV, Random, 95% CI)	0.13 [‐0.12, 0.39]
5 Health class achievement	1	263	Mean Difference (IV, Random, 95% CI)	‐0.05 [‐0.38, 0.29]
6 Inhibition control	2	110	Std. Mean Difference (IV, Random, 95% CI)	‐0.67 [‐1.50, 0.16]
7 Attention	1		Mean Difference (IV, Random, 95% CI)	Totals not selected
8 Visuo‐spatial abilities	1		Mean Difference (IV, Random, 95% CI)	Subtotals only
9 Non‐verbal memory	1		Mean Difference (IV, Random, 95% CI)	Subtotals only

**Comparison 3:** Dietary interventions versus standard practice

Outcome or subgroup title	No. of studies	No. of participants	Statistical method	Effect size
1 Average school achievement	2	434	Std. Mean Difference (IV, Random, 95% CI)	0.32 [‐0.07, 0.70]
1.1 Children with obesity	2	379	Std. Mean Difference (IV, Random, 95% CI)	0.45 [0.25, 0.66]
1.2 Children with overweight	1	55	Std. Mean Difference (IV, Random, 95% CI)	‐0.17 [‐0.70, 0.36]
2 Mathematics achievement	1	76	Mean Difference (IV, Random, 95% CI)	‐2.18 [‐5.83, 1.47]
3 Reading achievement	1	67	Mean Difference (IV, Random, 95% CI)	1.17 [‐4.40, 6.73]
4 Attention	1	61	Mean Difference (IV, Random, 95% CI)	1.68 [‐7.86, 11.22]

**Comparison 4:** Lifestyle intervention versus control

Outcome or subgroup title	No. of studies	No. of participants	Statistical method	Effect size
1 BMI z‐score	7		Mean Difference (IV, Random, 95% CI)	Totals not selected
1.1 Beneficial effect on school achievement	3		Mean Difference (IV, Random, 95% CI)	0.0 [0.0, 0.0]
1.2 No beneficial effect on school achievement	2		Mean Difference (IV, Random, 95% CI)	0.0 [0.0, 0.0]
1.3 Beneficial effects on cognitive functions	3		Mean Difference (IV, Random, 95% CI)	0.0 [0.0, 0.0]
1.4 No beneficial effect on cognitive functions	3		Mean Difference (IV, Random, 95% CI)	0.0 [0.0, 0.0]
2 Total body fat (%)	3		Mean Difference (IV, Random, 95% CI)	Totals not selected
2.1 Beneficial effect on cognitive functions	1		Mean Difference (IV, Random, 95% CI)	0.0 [0.0, 0.0]
2.2 No beneficial effect on cognitive functions	3		Mean Difference (IV, Random, 95% CI)	0.0 [0.0, 0.0]

## Characteristics of studies

### Characteristics of included studies [ordered by study ID]

**Table:** Ahamed 2007

Methods	Study design: Cluster‐randomised controlled trial Unit of randomisation: Schools, stratified by size and geographical location. N schools: 8 (6 intervention schools, 2 control schools) Duration of intervention: 16 months Follow‐up: After 1 school year Unit of analysis: Child Setting: British Columbia, Canada Exclusion criteria: "school already undertaking a school‐based physical activity program" Classification of weight status: CDC BMI‐for‐age growth charts Start date: April 2003 End date: June 2004	
Participants	N (randomly assigned): 103 (78 intervention, 25 control) N (analysed): 73 (52 intervention, 21 control) Age range: 9 ‐ 11 years (4th and 5th grades), mean age: 10.1 ± 0.6 years Sex: Intervention group: 48% female, control group 19% female Ethnicity: Not reported for subgroup with obesity Attrition (children): 29.1% Reasons for attrition: Children moved schools or were absent on the day of testing (5 times higher in intervention than control school), school chose not to send participants` test results to the CAT‐3 test centre for scoring (control school), school administered the wrong test at follow‐up (intervention school)	
Interventions	**Comparison: Action Schools! BC versus standard practice** **Intervention:** Action Schools! BC is a comprehensive, multicomponent intervention providing tools for schools and teachers to use in promoting physical activity and healthy eating in different settings. These include the school environment (healthy eating posters), scheduled Physical Education, classroom action, family and community (e.g. walking school bus), extracurricular activities (e.g. dance club) and school spirit (e.g. Hike across Canada challenge). Extracurricular and school spirit activities were provided by only a small number of intervention schools **1. Physical activity:** Classroom‐ and/or school environment–based physical activity for 15 minutes a day on 5 days/wk delivered by trained classroom teachers. Activities included hip hop dancing, skipping, jumping, chair aerobics, yoga and strength work. This activity was provided in addition to 40 minutes of Physical Education twice a week to engage children in 150 minutes of physical activity/wk. Compliance with intervention was assessed by the classroom teacher through daily physical activity logs reporting type, duration and frequency **2. Nutrition:** Across the different settings, a fruit and vegetable (F&V) intervention was employed that focused on increasing intake of F&V; improving knowledge, attitudes and perceptions regarding F&V; and strengthening willingness to try new F&V **Standard practice:** Usual educational school practice	
Outcomes	**Outcome 1: *School achievement:*** Total and subject‐specific scores for mathematics, reading and language, assessed using the Canadian Achievement Test (CAT‐3). The test was administered by classroom teachers and was scored for all but 1 school at the CAT‐3 test centre. 1 school scored the test locally **Outcome 2: *Obesity indices:*** Weight and height were measured and BMI calculated	
Notes	Authors kindly provided raw data for children with obesity or overweight Sample size calculation was performed for total sample (children with normal weight and overweight/obesity) Funding sources: 2010 Legacies Now and the BC Provincial Health Service Authority in collaboration with the BC Ministry for Health Research Scholar	
***Risk of bias***		
**Bias**	**Authors' judgement**	**Support for judgement**
Random sequence generation (selection bias)	Unclear risk	Quote (from report): "Schools were then remotely randomized..." Quote (from email correspondence): "randomisation was done by random number draw by a third party " Judgement comment: Although the method of randomisation is appropriate to reduce selection bias, baseline differences in school achievement between intervention and control schools occurred. Baseline imbalances are a risk in cluster RCTs and might indicate inappropriate randomisation of clusters. However, it remains unclear whether the imbalances occurred by chance.
Allocation concealment (selection bias)	Low risk	Quote (from email correspondence): "Yes, the 10 schools were randomized at once"
Blinding of participants and personnel (performance bias) All outcomes	Unclear risk	Quote (from email correspondence): "The primary purpose of Action Schools! BC was not to improve academic performance" Comment: Blinding of children and personnel regarding the experimental condition is not possible in a lifestyle intervention. Email correspondence with authors confirmed that participants and personnel were blinded to the true purpose of the study
Blinding of outcome assessment (detection bias) All outcomes	Low risk	Quote (from report): "CAT‐3 tests were administered by classroom teachers to [...] students in INT [intervention] and UP [usual practice] schools"
Incomplete outcome data (attrition bias) All outcomes	High risk	Comment: Authors provided raw data from complete baseline and follow‐up data sets only. Incomplete follow‐up data were therefore not imputed and included in the analysis. Characteristics of missing data were not provided
Selective reporting (reporting bias)	Low risk	Judgement comment: All prespecified achievement outcomes were reported
Comparability of baseline groups	Unclear risk	Quote: "Schools were stratified by size and geographic location. [...] to accommodate different organisational structure of large versus small schools and different ethnic demographics between regions" Judgement comment: Differences in baseline characteristics between experimental groups were not significant besides school achievement scores, which were higher in the control school than in the intervention school
Cross‐contamination	Low risk	Quote: "We recruited elementary schools from the Vancouver and Richmond school districts in British Columbia, Canada...Schools were stratified by size (< 300 or > 300 students) and geographic location (Vancouver or Richmond)." Judgement comment: cluster randomisation of school and inclusion of large school districts reduced the risk of cross‐contamination
Other bias	Low risk	Judgement comment: None detected

**Table:** Barbosa Filho 2017 [pers comm]

Methods	Study design: Cluster‐randomised controlled trial Unit of randomisation: Schools N schools: 6 (3 intervention schools, 3 control schools) Duration of intervention: 4 months Follow‐up: Immediately post‐intervention Unit of analysis: Child Setting: Fortaleza, northeastern Brazil Exclusion criteria: Students who are younger than 12 years old and older than 15 years old not being full‐time schools with the programme called Programa Saúde na Escola in Fortaleza, not enrolled in grade 7 ‐ 9 Classification of weight status: IOTF cut offs for overweight and obesity Start date: 2nd semester of 2014 for 4 months	
Participants	N (randomly assigned): Not reported N (analysed): 138 (75 intervention, 63 control) Age range: 13 ‐ 18 years Sex: Intervention group ‐ 43.4% male; Control group ‐ 47.6% male Ethnicity: Not reported Socio‐economic status: Intervention group ‐ Most affluent (top 2 quintiles), 22.4%, Most deprived (bottom 3 quintiles): 77.6%; Control group ‐ Most affluent (top 2 quintiles): 17.5%, Most deprived (bottom 3 quintiles): 82.5% Attrition rates: Data could not be obtained specifically for adolescents with obesity or overweight Reasons for attrition: Dropping out of school was the main reason for non‐participation in post‐ intervention data collection	
Interventions	**Comparison: "Fortaleça sua Saúde" programme versus standard practice** **Intervention:** Fortaleça sua Saúde ('Strengthen your health') focus on teachers' training and activities on health in curriculum, active opportunities in the school environment, and health education. The intervention was delivered by trained school teachers **1. Physical activity (PA):** a) Training and activities in PE classes: structure predominantly active PE classes, even in classes with a theoretical content. The manual included 4 units: (i) PA and health (e.g. PA and leisure, co‐operative games, PA with parents); (ii) health factors (e.g. sedentary time, diabetes and hypertension, quality of life); (iii) sports (e.g. athletics, volleyball, functional training, combat sports); and (iv) popular games (e.g. games,dancing and adventure sports) b) Active opportunities in the school environment: Supervised 10 – 15‐minute sessions, called “Gym in School”, were performed twice a week. These sessions were composed of physical (e.g. stretching, located exercises) or dynamic (e.g. games and rhythmic activities) activities in small and large groups. Space and equipment were structured and made available to play games in free time during the school day **2. Healthy lifestyle education:** a) Training and activities in the general curriculum: The manual included proposals for activities according to knowledge areas (i.e. languages, social sciences, natural sciences and mathematics). For example, in mathematics, there was a proposal about teaching quantities and measures using body measurements, energy expenditure in physical activity or energy consumption in meals. Teachers were encouraged to undertake the activities or to create and implement similar strategies in the classroom during the semester b) Health education in the school community: Pamphlets with messages about active and healthy lifestyle were distributed. 3 pamphlets were directed at students: (i) PA and health; (ii) screen time and health; and (iii) healthy eating and healthy behaviours. 2 pamphlets were directed at parents: (i) PA and parents/the family; and (ii) screen time and parents/the family **Standard practice:** "Schools from the control group underwent 1 semester with the regular and conventional activities of a full‐time school. In general, the control schools had 2 weekly Physical Eduction classes that included content and activities according to the perspective of their teachers."	
Outcomes	**Outcome 1: School achievement:** Academic performance was evaluated considering scores from standardised tests during a school year in 2 areas: **Mathematics and Language (Portuguese)**. The crude scores (ranging from 0 ‐ 10 points) of each student were obtained from the schools and organised by semester to indicate the pre‐intervention period and during/post‐intervention. Z‐scores for Mathematics and Language by school and grade were calculated and provided for inclusion in this review	
Notes	The authors kindly provided unpublished data for children with obesity or overweight Sample size calculation was performed for total sample (children with normal weight and overweight/obesity) Funding/Sponsor: Universidade Federal de Santa Catarina, Universidade Federal do Ceara, Secretaria Municipal de Educação de Fortaleza, Ceará, Brazil	
***Risk of bias***		
**Bias**	**Authors' judgement**	**Support for judgement**
Random sequence generation (selection bias)	Unclear risk	Quote: "Thus, we performed the random selection of three schools to each condition" Quote: "This is a cluster‐randomized controlled trial and the school was the sample selection unit." Judgement comment: Described as cluster but no methods described as to how this was achieved. Unclear how and if a random sequence was generated
Allocation concealment (selection bias)	Unclear risk	Judgement comment: No information provided
Blinding of participants and personnel (performance bias) All outcomes	High risk	Quote: "The first stage was a four‐hour training input that took place at the beginning of the school semester. There were discussions of primary health concepts and the importance of these issues including the relationship between health, school and academic performance." Judgement comment: This quote suggests that the teachers were aware of the potential impact of the intervention on academic performance. True blinding of participants and personnel is not possible for this kind of intervention. It is unclear if the participants were aware of the potential of the intervention influencing academic performance
Blinding of outcome assessment (detection bias) All outcomes	High risk	Quote: "The notes and standardized scores of the students were obtained from the schools and organized by semester to indicate the pre‐intervention period (the first semester of 2014) and during/post‐intervention (the second semester of 2014)." Judgement comment: The schools providing the academic achievement scores were aware of the group allocation
Incomplete outcome data (attrition bias) All outcomes	Low risk	Quote: "The main reason for non‐participation at baseline was being absent from school on data collection days. Post‐intervention data collection included 1,085 students (response rate of 93.2 % and 90.4 % in intervention and control schools, respectively). Dropping out of school was the main reason for non‐participation in post‐intervention data collection" Judgement comment: Quote from Barbose Filho 2016: " Dropouts tended to be older than participant students (P < .001, Table 1)."
Selective reporting (reporting bias)	Low risk	Quote: "ClinicalTrials.Gov: NCT02439827." Quote: "Barbosa Filho et al. BMC Public Health (2015) 15:1212" Judgement Comment: The citation refers to the published study protocol. The authors provided unpublished data of which all were listed as outcomes in the study protocol
Comparability of baseline groups	Unclear risk	Quote from Barbosa Filho 2016: "There were no significant differences between the intervention and control groups for almost all variables at baseline. The exception was a higher proportion of active commuting to school among students from the control group and inactive students among intervention group (all P < .001)." Judgement comment: It is unclear if this was also the case for the population group with overweight/obesity, subject of this review
Cross‐contamination	Low risk	Quote: "The six schools had similar characteristics (e.g., size, target audience, curriculum, etc.) and were located in different administrative regions (geographically dispersed)."
Other bias	Low risk	Judgement comment: None detected

**Table:** Chen 2016

Methods	Study design: Randomised controlled trial Unit of randomisation: Child Duration of intervention: 3 months Follow‐up: Immediately post‐intervention Unit of analysis: Child Setting: New Taipei City, Taiwan Exclusion criteria: Cardiovascular disease, asthma, or cardiac dysrhythmia diagnosed by a medical doctor, or a neurological or psychiatric disorder diagnosed by a psychiatric professional Classification of weight status: Age‐ and gender‐specific BMI on the 95th percentile of the updated national growth norm in Taiwan Start date: October 2013 End date: October 2014	
Participants	N (randomly assigned): 36 intervention 21 control N (analysed): 50 (25 intervention, 25 control) Age range: 12 ‐ 15 years Mean age: Intervention 12.84 ± 0.75 years; control 12.64 ± 0.70 years Sex: Intervention group 36% female Control group 52% female Ethnicity: Not reported Socio‐economic status: Intervention group ‐ Most affluent 20%, most deprived: 80% Control group ‐ Most affluent: 20%, Most deprived: 80% Reasons for attrition: Intervention group n = 3 could not stand the intensity, n = 3 not available on measurement day; control group n = 3 not available on measurement day Attrition rates: 19% in intervention group, 14% in control group	
Interventions	**Comparison: Aerobic group physical activity versus wait‐list control** **Intervention:** Group physical activity programme including multiple types of moderate‐intensity exercises performed 4 times/week for 40 minutes a session (5 minutes each for warm‐up and cool‐down, 30 minutes for the main exercise). "The participants were instructed to attend an instructor‐monitored physical activity programme with an instructor: participant ratio of 1:10 at 1 of 3 times (i.e. morning, lunch break, or after school) during the school day." "Each participant received a physical activity manual that consisted of 3 sections: instructions for the warm‐up, descriptions of the exercises, and a daily exercise log. The participant was free to choose 1 of the provided exercise types (e.g. fast walking, stair climbing, jumping rope, or aerobic dancing) each time, with an emphasis on maintaining a moderate intensity of 60% to 70% of the maximal heart rate (220 minus age). The target heart rate was progressively increased based on each participant’s ability to meet the optimised target heart rate. Daily recording of the characteristics of the exercise performed and of adverse events in the exercise log was also performed." **Wait‐list control:** Participation in regular health education course following randomisation; opportunity was given "to participate in a similar physical activity programme after the intervention duration"	
Outcomes	**Outcome 1: Cognitive flexibility** (set shifting) measures using the computer version of the Wisconsin card sorting test (Version 4—Research Edition) **Outcome 2: Total body fat:** Body fat was assessed using a Karada Scan body composition monitor (HBF362, Omron, Kyoto, Japan)	
Notes		
***Risk of bias***		
**Bias**	**Authors' judgement**	**Support for judgement**
Random sequence generation (selection bias)	Low risk	Quote: "The participants were randomly assigned to a physical activity program or a wait‐list control group using random allocation software."
Allocation concealment (selection bias)	Unclear risk	Judgement comment: No information reported
Blinding of participants and personnel (performance bias) All outcomes	High risk	Quote: "The current study employed a randomized controlled trial (RCT) design to determine the effects of a physical activity intervention on the set‐shifting aspect of executive function in obese young adolescents." Judgement comment: True blinding for a physical activity intervention is not possible. Whether the participants were blinded to the true purpose of the study, in relation to executive function and anthropometrics, is unclear. However, it is very likely that the personnel was aware of the study aims. Physical intervention and waiting list control within the same school means staff and pupil blinding to intervention not possible
Blinding of outcome assessment (detection bias) All outcomes	Unclear risk	Judgement comment: No description given
Incomplete outcome data (attrition bias) All outcomes	High risk	Judgement comment: Substantive attrition from both groups; data from outcomes are reported but intention‐to‐treat analysis is not discussed. Attrition after randomisation, before baseline assessment: intervention = 20%, control group = 14%. Therefore, unclear risk of bias
Selective reporting (reporting bias)	Unclear risk	Judgement comment: No reference to a predefined study protocol or trial register
Comparability of baseline groups	Low risk	Judgement comment: Adequate random sequence generation
Cross‐contamination	Unclear risk	Quote: "The participants were recruited from a specific junior high school" Judgement comment: Despite the intervention being delivered in instructor‐led sessions, it is unclear whether contact between intervention and control participants led to sharing of physical activities
Other bias	Low risk	Judgement comment: No other bias detected

**Table:** Damsgaard 2017 [pers comm]

Methods	Study design: Cluster‐randomised controlled cross‐over trial Unit of randomisation: Schools stratified by year and group (grade 3 or 4) N schools: 9 schools (46 classes) Duration of intervention: 3 months Follow‐up: Immediately post‐intervention at 3 months (before cross‐over) Unit of analysis: Child Setting: Zealand and Lolland‐Falster, Denmark Exclusion criteria: Disease or condition that obstructs measurements or puts children at risk if eating the diet, participation in other scientific studies involving radiation or blood sampling, schools with unsuitable kitchen facilities, schools not located in the eastern part of Denmark Classification of weight status: Based on IOTF cut‐offs for overweight and obesity Start date: November 2005 End date: April 2007	
Participants	N (randomised): 109 (57 intervention, 52 control) N (analysed): 53 (intervention), 50 (control) Age range: 9 ‐ 11 years (3rd and 4th grades) Sex**:** Intervention group ‐ 72% female; control group ‐ 59% female Overweight: Intervention group 82%; Control group 88% Obesity: Intervention group 28%; Control group 22% Ethnicity: Intervention group ‐ white 98%; control group ‐ white 94% Attrition rate: 7% in intervention; 4% in control Reasons for attrition: Withdrew during the study mainly because they changed school or class, disliked or found the measurements too time‐consuming, or disliked the intervention school meals. The proportion of children who withdrew from the study was not different between the two clusters	
Interventions	**Comparison: OPUS School Meal (New Nordic Diet) versus standard practice** **Intervention:** "The New Nordic Diet (NND) contains seasonal, health‐promoting ingredients, for example, berries, root vegetables, whole grains, fish, shellfish, seaweed and rapeseed oil. Diet contains less meat than average Danish diet." "Children received daily servings of a mid‐morning snack, ad libitum hot lunch meal and afternoon snack (twice/week fresh fruit, dried berries or both, and nuts and muesli bar or bread roll). The meals met 40% to 45% of daily energy intake based on energy requirements of 11‐year‐old children". The meals were produced locally at each school by trained chefs and kitchen personnel hired for the study. School lunch breaks were increased from 15 minutes to 20 ‐ 25 minutes. "The children were encouraged to taste everything and to keep a reasonable plate distribution with vegetables and starchy foods filling the majority of the plate." "Each child spent 3 – 5 school half‐days during the NND period in the kitchen cooking, presenting, and serving the menu of the day to the other children." The teachers were encouraged to participate in the lunch meals. "Class teachers were given a box of teaching materials about the human body, the clinical measurements, and taste sensorics, including background information about NND and suggestions for related educational activities and games." Use of the material was optional. **Standard practice:** Usual packed lunch	
Outcomes	**Outcome 1: School achievement:** Teacher‐assessed **mathematics and reading** proficiency using age‐specific Danish standardised tests **Outcome 2: Cognitive function:** Assessment of **attention** using the D2 Test of Attention. Unclear who administered the test	
Notes	The authors kindly provided unpublished data for children with obesity or overweight Follow‐up data after cross‐over period at 6 months not included in this review Power calculation performed for total study sample based on metabolic syndrome test score Funding source: Nordea‐fonden	
***Risk of bias***		
**Bias**	**Authors' judgement**	**Support for judgement**
Random sequence generation (selection bias)	Low risk	Quote: "Within each of the two blocks, schools were allocated to the order of treatment and control for third and fourth grades by simple randomisation. The randomisation list was performed by a statistician not involved in data collection or analysis using the statistical software package R (R Foundation for Statistical Computing)"
Allocation concealment (selection bias)	Low risk	Quote: "Cluster randomisation was performed before the children were invited for participation."
Blinding of participants and personnel (performance bias) All outcomes	High risk	Quote: "The allocation order was not blinded to investigators, schools or participants." Quote from the study protocol (Damsgaaard 2012): "OPUS School Meal Study was a cluster‐randomised controlled unblinded cross‐over study."
Blinding of outcome assessment (detection bias) All outcomes	High risk	Quote: "The allocation order was not blinded to investigators."
Incomplete outcome data (attrition bias) All outcomes	Unclear risk	Quote: "A total of sixty‐nine children (8.3 %) withdrew during the study mainly because they changed school or class (n 29), disliked or found the measurements too time‐consuming (n 17), or disliked the intervention school meals (n 13). The proportion of children who withdrew from the study was not different between the two clusters (intervention – control 10.2 % v. control – intervention 6.5 % of the participants, P< 0.054)." Judgement comment: The figures refer to the total study sample which included children with healthy weight. The authors confirmed during email correspondence that no intention‐to‐treat analyses were performed. The attrition rates were low, with 7% and 4% in intervention and control group, respectively. As indicated by the quote, attrition did not differ between the experimental conditions
Selective reporting (reporting bias)	Low risk	Quote: "The study protocol is registered at www.clinicaltrials.gov (NCT 01457794)." Quote: "A comprehensive description of the study design and recruitment of schools and participants has been provided previously." Judgement Comment: All outcomes reported in the trial register and the published study protocol (Damsgaard et al 2012) were reported in the Results publication
Comparability of baseline groups	Low risk	Judgement comment: Inspection of unpublished participant characteristics tables suggest that the experimental groups were comparable at baseline
Cross‐contamination	Low risk	Quote: "To avoid peer contamination of diets and to incorporate the intervention into the regular school schedule, randomisation was performed in clusters of year group at each school, i.e. either third or fourth grade pupils had the intervention period in the first study period, whereas the other year group had the intervention in the second study period." Quote from the study protocol (Damsgaard et al 2012): "Prior to study start, the class teachers were given a box of teaching materials about the human body, the clinical measurements, and taste sensorics, including background information about NND and suggestions for related educational activities and games. Use of the material was optional, but the teachers were instructed not to use the material about NND during the control period."
Other bias	Low risk	Judgement comment: None detected

**Table:** Davis 2011b

Methods	Study design: Randomised controlled trial Unit of randomisation: Child Duration of intervention: 13 weeks Follow‐up: Immediately after intervention Unit of analysis: Child Setting: Georgia, USA Exclusion criteria: Regular physical activity > 1 hour/wk, medical condition that affects outcome or limits intervention participation, participation in another study, on medication other than for attention‐deficit disorder Classification of weight status: CDC growth charts Start date: 2003 End date: 2006	
Participants	N (randomly assigned): 116 (56 intervention, 60 control) N (completed): 110 (54 intervention, 56 control) N (analysed): 116 (110 + 6 LOCF) Age range: 7 ‐ 11 years, Mean age: Intervention group 9.3 ± 1.1 years, Control group 9.4 ± 1.1 years Sex: Intervention group 54% female; Control group 62% female Ethnicity: Intervention group ‐ 64% black, 36% white Control group ‐ 58% black, 42% white Reason for attrition: Refused post‐test (N = 2 intervention, N = 3 control), excluded because of psychiatric illness (N = 1, control) Attrition: 5.2% (6/116)	
Interventions	**Comparison: Aerobic group exercise versus standard practice** **Intervention:** Aerobic exercise group for 40 minutes a day, 5 times a week, over a mean total of 13 weeks. 5‐minute warm‐up phase consisting of brisk walking and static and dynamic stretching. "Activities were selected on the basis of ease of comprehension, fun and eliciting intermittent vigorous movements. Children were encouraged to maintain a heart rate > 150 beats/minute during running games, tag games, jump rope, modified basketball and soccer." No competition or skill enhancement. Intervention session ended with a cool‐down including such activities as water break, slow walking and static stretching. "The intervention was delivered by qualified and trained research staff in an after‐school programme at the gymnasium of the Georgia Prevention Institute." Compliance was assessed by observing and recording attendance and average heart rate daily for each child. This study included a 2nd intervention group, which was not included in this review (see Notes) **Standard practice:** Continuation of usual activities "All participating families were offered a monthly lifestyle education class covering the topics of healthy diet, physical activity and stress management."	
Outcomes	**Outcome 1: School achievement:** Broad mathematics and reading skills on the Woodcock‐Johnson Tests of Achievement III **Outcome 2: Cognitive function:** Subcales for planning, attention, simultaneous successive use of the Das‐Naglieri‐Cognitive Assessment System. Both tests were administered by a qualified psychologist and personnel with graduate training in psychological assessment **Outcome 3: Obesity indices:** Quote "Body weight (in shorts and t‐shirt) and height (without shoes) were measured with an electronic scale (Detecto, Web City, MO) and stadiometer (Tanita, Arlington Heights, IL) and converted to BMI and a BMI z‐score (Epi Info, Centers for Disease Control and Prevention, Atlanta, 2003)"	
Notes	Sample size calculation performed. 62 participants per group were estimated to provide 80% power to detect a difference between groups of 6.6 units The 2nd intervention arm included a 20‐minute physical activity intervention followed by 20 minutes of sedentary activities, such as board games, card games and drawing (low‐dose intervention arm). This intervention group was excluded because the sedentary activities might have affected cognitive function without being defined as lifestyle interventions Funding sources: National Institutes of Health, State of Georgia Biomedical Initiative grant to the Georgia Center for Prevention of Obesity and Related Disorders, Medical College of Georgia and University of Georgia	
***Risk of bias***		
**Bias**	**Authors' judgement**	**Support for judgement**
Random sequence generation (selection bias)	Low risk	Quote (from report): "... children were assigned randomly by a statistician..." Quote (from the report Davis 2012): "...each participant was assigned a uniform (0,1) random number [...] within their respective ethnicity and sex group. If the number was between 0 and 0.33 the child was randomised to the low‐dose group; between 0.34‐0.67, to the high‐dose group; and above 0.67, to the control group"
Allocation concealment (selection bias)	Low risk	Quote (from email correspondence): "I ensured allocation concealment by not permitting randomization by the statistician until baseline testing was completed. Only then were they randomized and their assignments communicated to the study coordinator, who informed the families."
Blinding of participants and personnel (performance bias) All outcomes	Unclear risk	True purpose of the study was blinded by advertising it as "trial of aerobic exercise on child's health" (quote from report) Judgement comment: Blinding of children and personnel regarding experimental condition is not possible in a physical activity intervention
Blinding of outcome assessment (detection bias) All outcomes	Low risk	Quote (from report): "...Outcome assessors were unaware of child's experimental condition..."
Incomplete outcome data (attrition bias) All outcomes	Low risk	Judgement comment: Provided participant flow chart indicated similar missing data in intervention and control groups Quote (from report): "Analyses were conducted using the last observation carried forward imputation for the [...] children who did not provide posttest data"
Selective reporting (reporting bias)	Low risk	Judgement comment: All previously reported outcomes were reported
Comparability of baseline groups	Low risk	Judgement comment: Random sequence adequately generated
Cross‐contamination	Unclear risk	Judgement comment: No details were reported on to what extent the control group adhered to 'usual activities'.
Other bias	Low risk	Judgement comment: None detected

**Table:** De Greeff 2016

Methods	Study design: Cluster‐randomised controlled trial Unit of randomisation: School classes N classes: 24 (12 intervention, 12 control); (12 schools) Duration of intervention: 3 months Follow‐up: 6 months (mid‐term), 18 months (immediately post‐intervention) Unit of analysis: Child Setting: Northern part of The Netherlands Exclusion criteria: None reported Classification of weight status: IOTF cut‐offs for overweight and obesity Start date: October 2012 End date: May 2014	
Participants	N (randomly assigned): 118 (60 intervention, 58 control) N (analysed): 6‐month follow‐up: 55 (intervention), 57 (control) 18‐month follow‐up: 40 (intervention), 44 (control) Age range: 7 ‐ 9 years Sex: Intervention group 52% female; control group 69% female Ethnicity: Not reported Reasons for attrition: 2 schools dropped out for reasons unrelated to the intervention. No reasons for child‐level attrition (missing data) reported) Attrition rates: 6‐month follow‐up: 8% (intervention), 2% (control) 18‐month follow‐up: 33% (intervention), 24% (control)	
Interventions	**Comparison: Physically active academic lessons versus standard practice** **Intervention:** "Fit en Vaardig op school" (Fit and academically proficient at school) involved physically activity academic lessons which ran over 44 weeks in total over 2 school years with 3 lessons/week. The lessons "had a duration of 20 – 30 minutes, with 10 – 15 minutes spent on solving mathematical problems and 10 – 15 minutes spent on language. During the school holidays the lessons were not continued." Each lesson was "supported by a PowerPoint presentation and a manual describing the tasks in detail." In year 1 the intervention was delivered by specially‐trained primary/elementary school teachers; in year 2 the intervention was delivered by trained regular class‐room teachers. "The physical activities were aimed to be of moderate‐to‐vigorous intensity. During the lessons all children started with performing a basic exercise, such as jogging, hopping in place or marching. A specific exercise was performed when the children solved an academic task. For example, for mathematics, children had to jump 8 times to solve the multiplication ‘42’. For language, children had to perform a squat for every spelled letter in the word ‘dog’. After performing the specific exercise, children had to continue performing the basic exercise until the next academic task was shown." **Standard practice**: Usual mathematics and language class	
Outcomes	**Outcome 1: Cognitive function: Inhibition control** assessed using the Golden Stroop Colour and Word test. **Working memory** was assessed using the Digit span backward and Visual span backward tests (data not included in this review). **Cognitive flexibility** was measured using a modified version of the Wisconsin card‐sorting test. The test were administered by trained researchers	
Notes	The authors kindly provided unpublished data for children with obesity or overweight Sample size calculation was performed for total sample (children with normal weight and overweight/obesity) Funding: Ministry of Education, Culture and Science (ODB10015)	
***Risk of bias***		
**Bias**	**Authors' judgement**	**Support for judgement**
Random sequence generation (selection bias)	Unclear risk	Quote: "A second or third grade class from each school was randomly assigned to serve as an intervention group....The class that was not assigned to the intervention group was automatically classified as the control group." Quote from de Greef et al 2016b: "Randomization was performed by the national Bureau for Economic Policy Analysis that was not involved in the study." Judgement comment: No indication of the methodology used. Imbalances in baseline differences between intervention and control participants might indicate inappropriate randomisation
Allocation concealment (selection bias)	Unclear risk	Judgement comment: The authors do not report details on allocation concealment.
Blinding of participants and personnel (performance bias) All outcomes	High risk	Judgement comment: Physical intervention and intervention and control classes within the same school mean participant blinding impossible for staff and children Quote: "The aim of this study was to examine the effects of physically active academic lessons on cardiovascular fitness, muscular fitness and EF after 2 years." Judgement Comment: True blinding to a lifestyle physical activity intervention is not possible. Some degree of blinding to the true purpose of the intervention (i.e. improving executive function) if unknown to pupils and teachers. It is unclear whether the pupils and teachers in school were aware of the study aims in relation to executive function
Blinding of outcome assessment (detection bias) All outcomes	Low risk	Quote: "Each instructed researcher received a 2 h training to get familiar with the EF and physical fitness tests and were mostly blinded to the condition children had been allocated to (during 88.6% of the measurements)." Judgement comment: Intention was that outcome assessors were blinded ‐ successful for most
Incomplete outcome data (attrition bias) All outcomes	High risk	Quote: "Due to circumstances not related to the intervention, two schools did not start the second intervention period, resulting in a lower sample size at T2 for both the control and intervention group. A loss of two schools was taken into account during the power analysis." Judgement comment: The proportion of missing data is high at T2 (18‐months follow‐up) and higher in the intervention group (33%) compared to the control group (24%). No methods of imputing missing data were applied. At mid‐term assessment (6‐months follow‐up), attrition was low overall (0% ‐ 8%)
Selective reporting (reporting bias)	Unclear risk	Judgement comment: Data presented at all time points, but pre‐published protocol is not reported
Comparability of baseline groups	High risk	Quote: "The control group consisted of a higher percentage of third grade children [Chi ^2^ (1) = 5.22; P = 0.025] and was significantly older [t(497) = 2.24; P = 0.026] due to a difference in number of children within each class. No significant age differences were found when analysing the second and third grade children separately." Judgement comment: The comparability of the experimental groups is at risk of bias.
Cross‐contamination	Unclear risk	Quote: "A second or third grade class from each school was randomly assigned to serve as an intervention group. All children from that class participated in the intervention program. The class that was not assigned to the intervention group was automatically classified as the control group." Judgement comment: No description of to what extent cross‐contamination was controlled for, in particular in relation to teaching staff. Were the intervention and control classes taught by different teachers? Was the same teacher teaching throughout the intervention? Teachers of both experimental groups could have shared teaching approaches
Other bias	Low risk	Judgement comment: None detected

**Table:** Gallotta 2015

Methods	Study design: Cluster‐randomised controlled trial Unit of randomisation: Schools N schools: 3 (1 for each study arm); (13 classes) Duration of intervention: 5 months Follow‐up: Immediately post‐intervention Unit of analysis: Child Setting: rural area North of Rome, Italy Exclusion criteria: Learning and academic difficulties, attention‐deficit disorders, neurological and developmental disorders, dyslexia, medical conditions that would affect study results or limit physical activity Classification of weight status: Children in relation to their body fat mass percentage (FM%) according to the McCarthy’s age‐sex specific cut‐offs:overweight/obesity (FM% > 85th centile) Start date: Not reported End date: Not reported	
Participants	N (randomly assigned): 23 (traditional physical activity), 19 (co‐ordinative physical activity ‐ arm), 11 (control) N (analysed): 23 (traditional physical activity), 19 (co‐ordinative physical activity ‐ arm), 11 (control) Age range: 8 ‐ 11 years Sex: Traditional physical activity group ‐ 52% female Co‐ordinative physical activity group ‐ 53% female Control group ‐ 36% female Ethnicity: Not reported	
Interventions	**Comparison: Traditional physical activity versus control; Co‐ordinative physical activity versus control** **Intervention:** "Both physical activity (PA) interventions differed in type and mode of physical activities in which children were engaged, but they were equivalent in structure, overall duration and intensity, and consisted of two 1‐hour sessions/week." "PA interventions were designed by a Physical Educator who supervised 1 of the 2 weekly lessons; the other was conducted by the classroom teacher. The two PA interventions had the same structure, and included 15 minutes of warm‐up, 30 minutes of moderate‐to‐vigorous physical activities, [...] and 15 minutes of cool‐down and stretching." **1. Traditional physical activity group:** "The traditional group PA intervention consisted of continuous aerobic circuit training followed by a sub‐maximal shuttle run exercise. This lesson was focused on the improvement of cardiovascular endurance by performing different types of gaits (e.g. fast walking, running, skipping) without any specific co‐ordinative request. The traditional PA lesson provided changes in executive modalities and some variations of intensity designed to promote health, fitness, sensory‐motor, social and communicative development." **2. Co‐ordinative physical activity group:** "The co‐ordinative group PA intervention aimed to develop both motor control abilities and perceptual‐motor adaptation abilities, by combining demands on gross‐motor and manipulative control abilities and perceptual‐motor adaptation abilities (particularly kinaesthetic differentiation and response orientation). It consisted of the sport‐unspecific use of basketballs in the context of mini‐games. The basketballs were used in unconventional ways with varying game rules (e.g. use of foot‐eye co‐ordination techniques with basketballs). These lessons were focused on the development of psychomotor competences and expertise in movement‐based problem‐solving through functional use of a common tool (e.g. basketball), and considering various tasks that involved decision‐making motor tasks and manipulative ball‐handling skills (e.g. bouncing, throwing, receiving a ball, and their combination)." **Control:** No details reported	
Outcomes	**Outcome 1: Cognitive function:** Assessment of **attention** using the D2 Test of Attention **Outcome 2: Obesity indices:** Body fat percentage was measured by multifrequency bioelectrical impedance analysis (IOI 353).	
Notes	The authors kindly provided unpublished data for children with obesity or overweight Sample size calculation was performed for the total sample (children with normal weight and overweight/obesity) Funding: Department of Movement, Human and Health Sciences (year 2013 ‐ Cod. RIC042013), University of Rome‘‘Foro Italico’’, Rome, Italy	
***Risk of bias***		
**Bias**	**Authors' judgement**	**Support for judgement**
Random sequence generation (selection bias)	Unclear risk	Quote: "cluster randomization" ... "The unit of randomization was the participating school." Judgement comment: Method not sufficiently described
Allocation concealment (selection bias)	Unclear risk	No details reported
Blinding of participants and personnel (performance bias) All outcomes	High risk	Quote: "PA interventions were designed by a Physical Educator who supervised one of the two weekly lessons; the other was conducted by the classroom teacher." Judgement comment: If not the participants, then the personnel (classroom and physical education teachers) were aware of the true purpose of the study. No attention control mentioned with regards to blinding attempts
Blinding of outcome assessment (detection bias) All outcomes	Unclear risk	Judgement comment: No details reported
Incomplete outcome data (attrition bias) All outcomes	Unclear risk	Quote: "Underweight children were excluded from the analyses, therefore, the final sample consisted of 156 primary school students with 56 children (33 normal weight and 23 overweight/obese) in the Traditional PA group, 59 children (40 normal weight and 19 overweight/obese) in the Coordinative PA, and 41 children (30 normal weight and 11 overweight/obese) in the Control group." Judgement comment: The authors did not provide a CONSORT flow diagram and no details on attrition and missing outcome data. No discussion of intention‐to‐treat analysis. It appears, though, that there was some extent of attrition as the difference in sample size between random allocation and inclusion in data analysis (based on the original sample, not only those included in this review) is unlikely to be attributable to underweight only. The proportion of children with underweight would be rather high: 28% (traditional PA), 29% (co‐ordinative PA), 41% (control group)
Selective reporting (reporting bias)	Unclear risk	Judgement comment: No reference to a published study protocol made.
Comparability of baseline groups	Unclear risk	Judgement comment: No details reported on the comparability of the experimental groups in terms of participant characteristics
Cross‐contamination	Low risk	Quote: "The study was designed as a cluster randomized controlled intervention in all classes (from Grade 3 to Grade 5) of three primary schools in a rural area located about 50 km north of the city of Rome (Italy)."
Other bias	Low risk	Judgement comment: None detected

**Table:** Huang 2015

Methods	Study design: Randomised controlled trial Unit of randomisation: Child Duration of intervention: 13 months Follow‐up measurements: Mid‐term at six weeks and immediately after the intervention at 13 months (52 weeks) Unit of analysis: Child Setting: Odense, Denmark Classification of weight status: Overweight or obesity according to IOTF cut‐offs Start date: May 2012 End date: June 2013	
Participants	N (randomised): 115 (59 intervention, 56 control) N (allocated): 106 (55 intervention, 51 control) N (analysed): Post‐intervention: 86 (48 intervention, 38 control) Age range: 10 ‐ 13 years Sex: Intervention 52.7% female, control 58.8% female Ethnicity: Intervention: Danish 62% , Non‐Danish 38% Control: Danish 71%, Non‐Danish 29% Exclusion criteria: Participation in other studies related to risk factors of heart disease, children who follow a special school programme, use of weight‐reducing medicine within 3 months before baseline measurements, children with motor skill conditions that hinder participation in the intervention Reason for attrition: 51 out of 55 children who were allocated to the camp programme completed the six weeks. One child was injured before the camp started, one child dropped out, and two children were expelled from the camp. Attrition rate: Six‐week follow‐up: Intervention 7%, Control 16% 13‐month follow‐up: Intervention 13%, Control 26%	
Interventions	**Comparison: Odense Overweight Intervention Study Day Camp versus standard practice** **Intervention:** The day‐camp intervention comprised 2 parts: "an intensive 6‐week day camp intervention and a subsequent 46‐week family‐based intervention programme (52 weeks in total)." "Participants stayed at a day camp from 7.30 a.m. to 8.30 p.m. for 7 days/week." **1. Physcial activity:** In the day camp, "children were engaged in physical activity and sports" for at least 3 hours/day, achieving about 90 minutes of moderate‐to‐vigorous physical activity a day measured by accelerometry. After the day camp, 1 physical activity day was offered as part of the family‐based intervention programme **2. Healthy lifestyle classes:** In the day camp, topics covered were nutrition, physical activity and health, goal‐setting, etc. The family‐based intervention programme comprised 4 parents‐involved meetings targeting daily physical activity and dietary behaviour **3. Diet:** In the day camp, 3 meals and 3 snacks were prepared and served according to the national Danish dietary recommendations with no caloric restrictions **Standard practice/attention control:** The standard intervention consisted of 1 weekly fun‐based **physical activity** session (2 hours duration) for 6 weeks. One **health and lifestyle educational** session for the parents was delivered by a dietician and physical activity specialist	
Outcomes	**Outcome 1: Cognitive functions: Inhibition control** measured using the Stroop Color and Word Test, **non‐verbal memory** measured using the Rey Complex Figure Test (immediate recall trial) and **visuo‐spatial abilities** assessed using the Rey Complex Figure Test (Copy trial) **Outcome 2: Obesity indices:** change in **BMI z‐scores** based on measured weight and height and calculated based on the IOTF growth charts; change in **total body fat mass (%)** measured using Dual energy X‐ray absorptiometry (DXA) which was performed by an experienced operator on a GE Lunar Prodigy (GE Medical Systems, Madison, WI); **waist circumference** was measured between the lower costal margin and the lilac crest; hip circumference will be measured at the level of the greater trochanter	
Notes	1. Funding source: TrygFonden	
***Risk of bias***		
**Bias**	**Authors' judgement**	**Support for judgement**
Random sequence generation (selection bias)	Low risk	Quote: "The allocation sequence was generated by sex stratified concealed block randomization (1:1) with a block size of 2 to 6 (random permuted blocks)." Quote from the study protocol (Larsen et al 2014): "The randomization was generated using the web‐based software http://www.randomization.com and http://www.random.org"
Allocation concealment (selection bias)	Unclear risk	Quote from the study protocol (Larsen et al 2014) : "Due to consideration for the participating families, and to avoid additional dropout on this account, it was necessary to inform participants of allocation three weeks prior to baseline measurements. Thus, allocation concealment was not possible." Judgement comment: Although the authors refer to the term allocation concealment, the term is not used in the same way as the Cochrane 'Risk of bias' tool. What the authors describe is the early knowledge of the allocation which had already occurred
Blinding of participants and personnel (performance bias) All outcomes	Unclear risk	Judgement comment: Participants and personnel were not blinded to the group allocation but both groups received an active intervention and so the control group condition (standard intervention arm) can be considered to some extent as 'attention control'. The protocol paper described the control condition as "shorter‐term and less intensive intervention program compare to the day‐camp group. This reflected a minimal effort to intervene in the children’s lifestyle and did not differ considerably from other initiatives being launched in Danish municipalities." It is unclear if the participants and the personnel were aware of the potential effect of the intervention on executive functions.
Blinding of outcome assessment (detection bias) All outcomes	Low risk	Quote from the study protocol: "Researchers were blinded at all assessments...."
Incomplete outcome data (attrition bias) All outcomes	High risk	Quote: "The analyses were conducted according to the intention to treat principle. Mixed effects models allow the inclusion of partial data of participants who may have dropped out or who were unavailable to follow‐ups. No imputation of data was applied. Maximum likelihood estimation was used for all models." Judgement comment: Attrition: at 6‐week follow‐up: Intervention 7%, Control 16%; at 52‐week follow‐up: Intervention 13%, Control 26% The distribution of drop‐outs is not even
Selective reporting (reporting bias)	High risk	Judgement comment: According to the study protocol 3 cognitive functions were intended to be assessed which were not reported in the Results article: attention and processing speed using the Symbol Digit Modalities Test (SDMT) and attention and executive function using the Trail Making Test A & B (TMT A & B). However, the Results article reported findings on executive functions obtained from the Behavior Rating Inventory of Executive Function (BRIEF), which was not prespecified in the study protocol (Larsen et al 2014)
Comparability of baseline groups	Low risk	Quote: "There were no significant between‐group differences on those characteristics at baseline."
Cross‐contamination	Low risk	Judgement comment: The experimental conditions varied substantially in terms of setting and timing and so cross‐contamination was unlikely.
Other bias	High risk	Quote: "It is noteworthy that six children who were slightly below the IOTF overweight cut‐points at screening were also suggested by the school nurses to participate in the OOIS intervention. This was due to the fact that the nurses thought that these children were at risk of being over‐ weight. Because the six children were included in the randomization, they were not excluded from the analyses." Judgement comment: Protocol violation for 6/115 participants coupled with high attrition predicts a high risk of bias

**Table:** Johnston 2013

Methods	Study design: Cluster‐randomised controlled trial Unit of randomisation: Schools N schools: 7 (4 intervention schools, 3 control schools) Duration of intervention: 2 years Follow‐up: Immediately after intervention Unit of analysis: Child Setting: Texas, USA Exclusion criteria: Not reported Classification of weight status: Data tables provided by the CDC Start date: Fall 2008 End date: Fall 2010	
Participants	N (randomly assigned): 321 (N intervention 186, N control 135) N (followed): 253 (N intervention 153, N control 100) Age: 7 ‐ 9 years, mean age: 7.8 ± 0.4 (intervention group), 7.7 ± 0.4 (control group) Sex: Intervention group 38.2% female, control group 45.9% female Ethnicity: intervention group Hispanic 27.4%, black 26.9%, Asian 24.3%, white 21.5% Control group Hispanic 29.6%, black 26.7%, Asian 16.3%, white 27.4% Reasons for attrition: Absent at follow‐up (N intervention 14, N control 11), no longer at school (N intervention 19, N control 24) Attrition: 21% (68/321)	
Interventions	**Comparison: Lifestyle education versus standard practice** **Intervention:** Whole‐school lifestyle education programme facilitated by a health professional involving curriculum material taught by trained teachers, school meal modification and nutrition counselling. Compliance with the intervention was assessed through direct weekly observation of teachers by the health professional and verbal self‐report from teachers **1. Nutrition/Diet:** Modification of school meals towards nutrient‐dense food. Nutrition counselling was provided on an informal basis by a school nurse **2. Health lifestyle education:** Teachers were provided with 50 integrated lessons‐worth of curriculum material aiming to improve healthy diet (increased fruit and vegetable, breakfast, healthy snack, water consumption) and increase physical activity. Teachers were encouraged to teach lifestyle integrated lessons once a week, to conduct health‐related activities every 2 weeks and to hold a school‐wide health event once a semester. The intervention component included provision of additional health information at school functions by health professionals and involvement of school libraries, computers, art, music and physical education in delivery/complementation of lifestyle education. **Standard practice:** "Even though intervention material was provided to control schools, teachers reported using the material once a month or less often."	
Outcomes	**Outcome 1: School achievement:** End‐of‐year final grades for reading, mathematics and science summarised as the GPA obtained from school records. The grade scale comprises scores between 0 and 100 points for each participant **Outcome 2: Obesity indices:** Age‐ and gender‐specific BMI percentiles and BMI z‐scores obtained from measured weight and height and by using formulas and data tables provided by the CDC. Overweight was defined as a BMI ≥ 85th percentile	
Notes	Authors were contacted No sample size calculation was reported. This study might therefore be at risk of a type two error Funding source: Not disclosed	
***Risk of bias***		
**Bias**	**Authors' judgement**	**Support for judgement**
Random sequence generation (selection bias)	Low risk	Quote: "7 schools were randomized using a random number generator"
Allocation concealment (selection bias)	Unclear risk	Judgement comment: No information provided
Blinding of participants and personnel (performance bias) All outcomes	High risk	Judgement comment: Blinding is not possible in lifestyle interventions. Unclear whether participants and personnel were blinded to the purpose of the study (in relation to the outcome of school achievement)
Blinding of outcome assessment (detection bias) All outcomes	High risk	Quote: "Student year‐end final grades for reading, math, and science were obtained from the school." Judgement comment: Teachers in the school were aware of the group allocation
Incomplete outcome data (attrition bias) All outcomes	Low risk	Quote: "Models were developed for both completers and intention‐to‐treat using the last observation carried forward (LOCF) method"
Selective reporting (reporting bias)	Unclear risk	Judgement comment: No information provided
Comparability of baseline groups	Low risk	Quote: "No differences were found between conditions with respect to baseline demographic or anthropometric variables" Judgement comment: Baseline GPA of intervention and control groups indicated no statistically significant differences between experimental groups
Cross‐contamination	Unclear risk	Quote: "All elementary schools (N=41 schools) from a large suburban independent school district located southwest of Houston, TX were recruited to participate in the study." Judgement comment: The geographic proximity of the schools is unclear
Other bias	Low risk	Judgement comment: None detected

**Table:** Krafft 2014

Methods	Study design: Randomised controlled trial (balanced by race, sex, and school to avoid imbalances on factors linked with differences in achievement) Unit of randomisation: Child Intervention duration: 8 months (average number of days offered 138 ± 9) Follow‐up: Immediately post‐intervention Unit of analysis: Child Setting: Georgia, USA Exclusion criteria: Any medical condition that would limit physical activity or affect study results (including neurological or psychiatric disorders) Classification of weight status: CDC growth charts for overweight and obesity Start date: May 2008 End date: April 2014	
Participants	N (randomised): 175 N (analyses): No details reported for relevant outcomes Age range: 8 ‐ 11 years, mean age: Intervention 9.7 ± 0.8 years; control 9.9 ± 0.9 years Sex: Intervention 71% female; control 58% female Ethnicity: 84% African American, 16% white Reason for attrition: Not reported Attrition rates: Not reported	
Interventions	**Comparison: Aerobic group exercise versus attention control** "Both groups were offered an after‐school programme every school day. All participants were transported by bus daily after school to the Georgia Prevention Center where they spent half an hour on supervised homework time and were provided with a snack. Both groups could earn points that were redeemed for small prizes weekly for performing desired behaviours. The reward schedule was periodically calibrated to keep the rewards offered to the groups similar." **Intervention:** "The aerobic exercise group engaged in instructor‐led aerobic activities (e.g. tag and jump rope) for 40 minutes a day. They wore heart‐rate monitors every day [...] with which they could monitor their own performance and from which data were collected daily. Points in the exercise group were earned for an average daily heart rate above 150 beats a minute, with more points for higher average heart rates." **Attention control:** Participants "engaged in instructor‐led **sedentary activities (e.g. art and board games)**. Points in the control group were earned for participation and good behaviour."	
Outcomes	**Outcome 1: Cognitive function:** The CAS was administered to assess **composite executive function** (Planning scale); **attention** (Attention scale), **non‐verbal memory** (Successive processing scale), and **visuo‐spatial abilities** (Simultaneous processing scale) **Outcome 2: Obesity indices:** Body fat was measured with a dual‐energy X‐ray absorptiometry (DXA) scan using a Hologic Discovery W (Hologic, Bedford, MA)	
Notes	We contacted the authors to obtain additional study details. We were not able to obtain details of outcome data for inclusion in the meta‐analysis, or additional study characteristics. A manuscript with relevant data is currently 'under review'. We did extract additional details from Bustamante 2016. Funding sources: National Institutes of Health (R01 HL87923) and the National Science Foundation Graduate Research Fellowship Program.	
***Risk of bias***		
**Bias**	**Authors' judgement**	**Support for judgement**
Random sequence generation (selection bias)	Low risk	Quote: "Randomization (balanced by race, sex, and school to avoid imbalances on factors linked with differences in achievement) was performed by the study statistician and concealed until after baseline testing was completed, at which point the study coordinator informed the families."
Allocation concealment (selection bias)	Low risk	Quote: "Randomization (balanced by race, sex, and school to avoid imbalances on factors linked with differences in achievement) was performed by the study statistician and concealed until after baseline testing was completed, at which point the study coordinator informed the families."
Blinding of participants and personnel (performance bias) All outcomes	Unclear risk	Quote:"The attention control group engaged in instructor‐led sedentary activities (e.g., art and board games)." Judgement comment: There is an attempt at attention control. Blinding to this kind of intervention is not possible
Blinding of outcome assessment (detection bias) All outcomes	Unclear risk	Judgement comment: No details regarding the blinding of outcome assessors reported. Details could not be obtained from study authors
Incomplete outcome data (attrition bias) All outcomes	High risk	Judgement comment: 11/54 drop‐outs polarised to the control condition: 1 vs 4 after baseline. We could not obtain information on the reasons for attrition and whether the authors dealt with missing data
Selective reporting (reporting bias)	Unclear risk	Judgement comment: No reference to a published study protocol or trial register made. However, the authors mention that this study builds on the study by Davis 2011b which is included in this review. The cognitive outcomes variables are similar in this study compared to Davis 2011b
Comparability of baseline groups	Low risk	Judgement comment: Random sequence adequately generated and allocation concealed
Cross‐contamination	Unclear risk	Quote: "The groups differed in that they participated in either an aerobic exercise or an attention control program. The aerobic exercise group engaged in instructor‐led aerobic activities (e.g., tag and jump rope) for 40 min per day." Judgement comment: Risk of cross‐contamination was low due to the nature of the intervention group: closed group‐exercise sessions. However, no details were reported on the extent to which the comparison group adhered to the 'sedentary activities' condition throughout the study duration
Other bias	Unclear risk	Judgement comment: Insufficient study details reported

**Table:** Melnyk 2013

Methods	Study design: Cluster‐randomised controlled trial Unit of randomisation: Schools N schools: 11 (distribution between intervention and control unclear) Intervention duration: 15 weeks Follow‐up: Immediately post‐intervention Unit of analysis: Adolescent Setting: Arizona, USA Exclusion criteria: Aged < 14 years (unlikely to have sufficient cognitive development to benefit from the proposed intervention), aged > 16 years (cognitive development of and social expectations for older teens requires a more complex and flexible intervention, potentially unavailable for 12‐month follow‐up sessions), medical conditions that would prevent them from participating in the physical activity component of the programme Classification of weight status: CDC growth charts for overweight and obesity Start date: January 2010 End date: December 2012	
Participants	N (randomised): 331 (161 intervention, 170 control) N (analysed): 263 (129 intervention, 134 control) Age range: 14 ‐ 16 years Mean age: Intervention 14.8 ± 0.8 years, control 14.7 ± 0.7 years Sex: Intervention 54% female, control 48% female Ethnicity: Intervention: Hispanic 79.5%, black/African American 9.9%, white 7.5%, Native American 1.2%, Asian 1.2%, other 0.6% Control: Hispanic 66.5%, black/African American 10.0%, white=12.9%, Native American 5.9%, Asian 4.1%, other 0.6% Reason for attrition: no longer at school, missed measurement days, asked to be withdrawn (no reasons reported), did not receive the intervention (no reason reported) Attrition rates: None reported	
Interventions	**Comparison: COPE versus attention control** "The attention control programme was administered in a format like that of the COPE intervention and included the same number and length of sessions as the experimental programme, but there was no overlap of content between the 2 programmes." **Intervention:** The Creating Opportunities for Personal Empowerment (COPE) programme "is a manualised 15‐session educational and cognitive–behavioural skills‐building programme delivered by trained high‐school health teachers." **1. Physical activity:** "Each session of COPE contains 15 – 20 minutes of physical activity (e.g. walking, dancing, kick‐boxing movements), not intended as an exercise training programme, but rather to build beliefs in the teens that they can engage in and sustain some level of physical activity on a regular basis. Pedometers were used throughout the intervention in order to reinforce the physical activity education component of COPE. Students were asked to increase their step counts by 10% each week, regardless of baseline levels and to keep track of their daily steps on a tracking sheet so they could calculate a weekly average and determine if they met their weekly goal." **2. Healthy lifestyle education:** The COPE Healthy Lifestyles TEEN (Thinking, Emotions, Exercise, Nutrition) Programme "was delivered once a week in students' health course for 15 weeks." "Participants received a COPE manual with homework activities for each of the 15 sessions that reinforced the content and skills in the programme." "A parent newsletter describing the content of the COPE programme was sent home 4 times during the course of the 15‐week programme." **Cognitive‐behavioural skills building:** Self‐esteem; positive thinking/self‐talk; goal‐setting; problem‐solving; stress and coping; emotional and behavioural regulation; effective communication; personality and communication styles; barriers to goal progression and overcoming barriers **Nutritional topics**: Food groups and a healthy body; stoplight diet: red, yellow, and green; nutrients to build a healthy body; reading labels; effects of media and advertising on food choices, portion sizes; influence of feelings on eating; social eating; strategies for eating during parties, holidays, and vacations; snacks; eating out **Physical activity topics**: Energy balance; ways to increase physical activity and associated benefits; heart rate; stretching **Attention control:** "The Healthy Teens programme was designed as a 15‐week attention control programme to control for the time the health teachers spent in the COPE group. The content was manualised and focused on safety and common **health topics**/issues for adolescents, such as **road safety, dental care, infectious diseases, immunisations, and skin care**." Participants "also received a manual with homework assignments each week that focused on the topics being covered in class and were asked to review with his or her parent a newsletter that was sent home with the teens 4 times during the programme." "Attention control participants were provided with a pedometer for use only during the first week and post‐intervention week for assessment purposes only."	
Outcomes	**Outcome 1: School achievement:** Health class grades assessed by school teachers	
Notes	The authors kindly provided unpublished data for adolescents with obesity or overweight The sample size calculation was based on the total study sample (participants in any weight group) Funding source: National Institute of Health/ National Institute of Nursing Research 1R01NR012171.	
***Risk of bias***		
**Bias**	**Authors' judgement**	**Support for judgement**
Random sequence generation (selection bias)	Unclear risk	Quote: "randomly assigned to receive either the COPE TEEN program or the Healthy Teens attention control program by placing all of the school names in a hat and then randomly drawing them out." Judgement comment: Restricted randomisation. The method is random but it could be easily manipulated. Imbalances in baseline differences between intervention and control participants might indicate inappropriate randomisation
Allocation concealment (selection bias)	Unclear risk	Quote: "The Healthy Teens attention control program by placing all of the school names in a hat and then randomly drawing them out." Judgement comment: Names are essentially concealed in the hat, which offers randomisation and allocation concealment. However names could easily be re‐drawn from the hat. It is unclear who performed the randomisation
Blinding of participants and personnel (performance bias) All outcomes	Unclear risk	Judgement comment: The trial register entry indicated that this was a double‐blind (Participant, Investigator) trial. Delivering health‐related content in a curriculum‐based Health Class might be a way of blinding the participants. However, the COPE intervention arm also offered active physical activity sessions to which, by nature, participants cannot be blinded
Blinding of outcome assessment (detection bias) All outcomes	Unclear risk	Quote: "Academic achievement was measured with the student's health course grade." Judgement comment: It is unclear if the same teacher who delivered the intervention also assessed academic performance in the health course
Incomplete outcome data (attrition bias) All outcomes	Unclear risk	Judgement comment: Academic achievement data were only available at post‐intervention, so assessment of missing data was not possible
Selective reporting (reporting bias)	Low risk	Judgement comment: NCT01704768 registry entry. All relevant outcomes were reported in the study protocol. The authors provided the unpublished outcomes data for adolescents with overweight/obesity
Comparability of baseline groups	High risk	Quote: "There are more male parents participating in the Healthy Teens group than the COPE TEEN group (p = .00). More parents are Hispanic in the COPE TEEN group versus the Healthy Teens group (p = 00). COPE TEEN parents have lower education levels (p = .00) and report more public assistance (p = .00) than Healthy Teens parents. COPE TEEN parents reported lower annual household incomes (p = .00)." Judgement comment: The quote relates to the entire study sample. The authors provided demographic characteristics of the subgroup with overweight/obesity which show similar differences between the experimental groups. The comparability of the experimental groups is at high risk of bias
Cross‐contamination	Low risk	Quote: "The first school district is located in the heart of the metropolitan city with the other district being located within a large suburb, which serves students from all socioeconomic backgrounds." Quote: "The decision was made to randomly assign schools to one of the two interventions (e.g., instead of randomly assigning classrooms within the schools) in order to decrease the probability of cross‐contamination and minimize threats to internal validity."
Other bias	High risk	Quote: "District administrators in both districts chose which schools could participate in the study." Quote: "All participants received incentives for their involvement in the intervention." Judgement comment: Selection bias introduced by financial incentives offered and the selection of schools by District administrators

**Table:** Nanney 2016

Methods	Study design: Cluster‐randomised controlled trial Unit of randomisation: Schools N schools: 8 (4 intervention, 4 control) Intervention duration: 1 year Follow‐up: Immediately post‐intervention Unit of analysis: Adolescents Setting: Rural Minnesota, USA Classification of weight status: CDC growth charts for overweight and obesity Start date: 2012 End date: 2014	
Participants	N (randomised): 323 (175 intervention, 148 control) N (analysed): 173 (95 intervention, 78 control) Age range: 15 ‐ 17 years Mean age: Intervention 15.2 ± 0.8 years, control = 15.2 ± 0.7 years Sex: Intervention 51%female, control 51% female Ethnicity (non‐white): Intervention 46%, control 36% Exclusion criteria: Already eating breakfast from any source at least 4 days in a usual school week, no access to the Internet or phone Reason for attrition: Not reported Attrition rates: Intervention 9%, control 29%	
Interventions	**Comparison: Project breakFAST versus wait‐list control** **Intervention:** "Aimed to improve student school breakfast programme (SBP) participation by ameliorating the following environmental factors in the high‐school setting." "As part of the Project breakFAST (Fueling Academics and Strengthening Teens) a grab‐and‐go style cart or breakfast line located outside the cafeteria in a high‐traffic hallway, atrium or common area was implemented, developed individually at each intervention school to meet unique needs of each school." "School‐wide marketing campaigns were developed by a community partner which worked with a group of students to design the marketing campaign at each intervention school." "Positive interactions and social support were created by developing school policies, if not already in place, to allow students to eat breakfast in the hallway. Schools were also encouraged to allow eating breakfast in some classrooms when appropriate. Teachers and school staff were asked to encourage the breakfast programme." "Development of a School Breakfast Expansion Team was encouraged at each intervention school. These teams were to consist of a variety of contributors including, but not limited to, the principal, food service director, nurse, students, wellness co‐ordinator, and teachers." "Extension Co‐ordinators were to provide support to schools in intervention development and implementation, communicated progress, successes." **Wait‐list control/standard practice:** Delayed treatment for the 1st year of follow‐up. "Schools implemented a modified form of the intervention in the 2nd year of follow‐up" (follow‐up data not included in this review). "Comparison schools received the same monetary incentive as intervention schools, as well as research study staff support in implementing the delayed intervention. The main difference was the marketing package offered to intervention schools, but not to comparison schools."	
Outcomes	**Outcome 1: School achievement:** assessed using weighted cumulative GPA. GPA covers academic years since 9th grade: pre‐cumulative GPA covers 1 academic year for 9th‐graders and 2 academic years for 10th‐graders; post‐cumulative GPA covers 2 academic years for 9th‐graders (became 10th‐graders at post‐test) and 3 academic years for 10th‐graders (became 11th‐graders at post‐test). The scale range for unweighted GPA was 0 ‐ 4	
Notes	The authors kindly provided unpublished data for adolescents with obesity or overweight The sample size calculation was based on the total study sample (participants in any weight group) Funding source: National Heart, Lung and Blood Institute of the National Institutes of Health (5R01‐ HL113235‐03).	
***Risk of bias***		
**Bias**	**Authors' judgement**	**Support for judgement**
Random sequence generation (selection bias)	Low risk	Quote: "The process of randomization of schools to treatment assignment was blocked so that 4 schools were assigned to intervention and 4 to control in each wave. As of the start of wave 1, only 13 schools had been recruited (see 4. Limitations), so a simple random subsample of 8 was assigned to wave 1. Within this subsample of 8, schools were randomly assigned to treatment or control by selection of a random permutation of the 8 labels (4 intervention, 4 control).For wave 2, three additional schools had been recruited prior to the randomization for wave 2, for a total of 8 schools. " Judgement comment: Adequate method used for wave 1 randomisation. Violation of randomisation procedure for wave 2 does not affect the data included in this study. We included wave 1 end‐point data only
Allocation concealment (selection bias)	Unclear risk	Judgement comment: No details reported
Blinding of participants and personnel (performance bias) All outcomes	Unclear risk	Judgement comment: Blinding to this kind of intervention is not possible. However, it is unclear if participants and personnel were aware of the potential benefits of the intervention on academic performance. The primary aim of the study was to increase breakfast uptake, whereas change in academic performance was an exploratory variable rather than a primary or secondary outcome
Blinding of outcome assessment (detection bias) All outcomes	High risk	Quote: "Each school received an excel file of variables and definitions requested. For each student, demographic information (e.g., race, ethnicity, grade level); grade point average (GPA) (term/semester GPAs and cumulative GPAs)..." Judgement comment: Schools and so teachers were aware of group allocation
Incomplete outcome data (attrition bias) All outcomes	High risk	Judgement comment: The authors provided the raw data for the children with obesity or overweight. We applied LOCF methods as an intention‐to‐treat approach. The attrition rates were 9% in the intervention group, and 29% in the control group
Selective reporting (reporting bias)	Low risk	Judgement comment: The unpublished data provided align with the outcomes reported in the study protocol
Comparability of baseline groups	Low risk	Judgement comment: The provided unpublished data of baseline characteristics in both experimental groups indicate a low risk of bias for comparability of the groups at baseline
Cross‐contamination	Low risk	Quote: "During the first year of follow‐up for each wave, four fidelity observations of both the intervention and delayed intervention groups were conducted to evaluate adherence to requirement of either making these necessary changes to the SBP (intervention) or not making any changes to regular breakfast service (control)." Judgement comment: Cluster randomisation and assessment of adherence to the experimental condition suggest a low risk of cross‐contamination bias
Other bias	Low risk	Judgement comment: None detected

**Table:** Resaland 2016

Methods	Study design: Cluster‐randomised controlled trial Unit of randomisation: School N schools: 60 (30 intervention, 30 control) Intervention duration: 4 months Follow‐up: Immediately post‐intervention Unit of analysis: Children Setting: Norway Classification of weight status: Not reported Start date: April 2014 End date: June 2015	
Participants	N (randomised): 229 (119 intervention, 110 control) N (analysed): 218 (117 intervention, 101 control) Age range: 10 ‐ 11 years Mean age: Intervention 10.2 ± 0.3, control 10.2 ± 0.3 Sex: Intervention 47% female, control 50% Ethnicity: Data not collected; birth place Norway: Intervention 93%, control 94% Reason for attrition: Not specific for the subgroup: moving away, no other reasons for withdrawal or drop‐out reported Attrition rates: Intervention 2%, control 8%	
Interventions	**Comparison: Active Smarter Kids programme versus standard practice** **Intervention:** The Active Smarter Kids (ASK) programme comprised 3 components aimed at providing children with the opportunity to engage in 165 minutes of physical activity/week more than the control group: i) **physically‐active lessons** for 90 minutes/ week, conducted in the playground; physically‐active educational lessons were delivered in 3 core subjects – Norwegian (30 minutes/ week), mathematics (30 minutes/week) and English (30 minutes/week); ii) **physical‐activity breaks** (5 minutes/day) implemented **in the classroom during academic lessons**; and iii) **physical‐activity homework** (10 minutes/day) prepared by teachers. "In addition, pupils participated in the curriculum‐prescribed 90 minutes/week of Physical Education and the curriculum‐prescribed 45 minutes/week of physical activity. Thus, PA (165 minutes/week) and PE/PA (135 minutes/week) components provided children opportunities to engage in school‐based physical activities 300 minutes/week. The intervention was established as part of the mandatory school curriculum for all pupils attending the intervention schools." **Standard practice:** “normal practice” school curriculum, including usual amounts of **physical activity/Physical Education**, being approximately 135 minutes/week	
Outcomes	**1. School Achievement**: Reading, numeracy, and English were measured using specific standardised Norwegian National tests designed and administrated by The Norwegian Directorate for Education and Training **2. Cognitive functions** (**measured but not provided**): Inhibition assessed using Golden’s version of the Stroop test; cognitive flexibility using 1 verbal (Verbal fluency) and 1 nonverbal test (The Trail Making Test); working memory used a digit span test with digits both forward and backward (Wechsler Intelligence Scale for Children, 4th edition) **3. Obesity indices** (**measured but not provided for analysis**): Weight/height: BMI; waist circumference; body fat (skinfold thickness sites ‐ biceps, triceps, subscapular, and suprailiac)	
Notes	The authors kindly provided unpublished school achievement data for children with obesity or overweight Cognitive function data were not provided as the authors were in the process of publishing them The sample size calculation was based on the total study sample (participants in any weight group) Funding sources: The Research Council of Norway (ID number 221047/F40) and Sogn og Fjordane University College	
***Risk of bias***		
**Bias**	**Authors' judgement**	**Support for judgement**
Random sequence generation (selection bias)	Unclear risk	Quote: "cluster‐randomized controlled trial (cluster RCT) with a random allocation at the school level using a 1:1 ratio." Quote from the study protocol Resaland 2015: "A neutral third party (Centre for Clinical Research, Haukeland University Hospital, Norway) performed the randomization." Judgement comment: It remains unclear how the random sequence was generated.
Allocation concealment (selection bias)	Unclear risk	Judgement comment: No details reported
Blinding of participants and personnel (performance bias) All outcomes	High risk	Quote: "Fifth‐grade classroom teachers in the intervention schools (I‐ schools) delivered the intervention. To support and qualify teachers to conduct the intervention, we arranged three comprehensive pre‐intervention seminars and two regional refreshing sessions during the intervention period. We also gave support via email and telephone to teachers in I‐schools. A password‐protected homepage (http://www. askstudy.no) further provided teachers in I‐schools with information, videos and content for approximately 100 PA lessons. All lessons on the homepage were developed in collaboration with I‐schools in Sogn og Fjordane County. Finally, we provided all I‐schools with equipment (e.g., laminating machines and accessories, mathematics bingo tiles, cones) necessary to support the intervention." Quote from the study protocol: "Blinding of children and schools was not possible due to the nature of the experiment."
Blinding of outcome assessment (detection bias) All outcomes	Low risk	Quote from the study protocol: "Blinding of children and schools was not possible due to the nature of the experiment. However, only the project management group has formal knowledge of group assignment. The data manager and statisticians are blinded to group allocation until analyses are conducted." Quote: "Academic performance in numeracy (often referred to as mathematics in the literature), reading and English was measured using standardized Norwegian national tests designed and administered by The Norwegian Directorate for Education."
Incomplete outcome data (attrition bias) All outcomes	High risk	Judgement comment: Based on the provided unpublished data, the proportion of missing data was substantially higher in the control group compared to the intervention group: maths Intervention 3%, control 7%; reading Intervention 0%, control 14%, English Intervention 0%, control 6%. No reason for missing data were provided. No imputation of missing data was performed for unpublished data
Selective reporting (reporting bias)	Low risk	Quote: "The study is registered in Clinicaltrials.gov ID nr: NCT02132494. We previously published a detailed description of the study (Resaland et al., 2015)...." Judgement comment: The authors provided unpublished academic achievement data which align with the study protocol and trial register. The authors clarified that they are working on the publication of executive function outcomes
Comparability of baseline groups	Low risk	Quote: "Table 1 shows children's baseline characteristics by group. There were no differences between I‐schools and C‐schools for any variables." Judgement comment: This quote relates to the total study sample including children with healthy weight. Visual inspection of participant characteristics with obesity or overweight (provided unpublished data) indicate a low risk of bias for comparability of the experimental groups at baseline
Cross‐contamination	Unclear risk	Quote: "ASK was a seven‐month cluster‐randomized controlled trial (cluster RCT) with a random allocation at the school level using a 1:1 ratio. Such randomization eliminated the possibility of contamination between pupils in the same school." Quote from the study protocol: "ASK teachers at the 28 I‐schools completed a report each week that described activities performed throughout the school day, the intensity of the activities (on a 1 to 3 scale) and the number of minutes allocated to physical activity/PE in each ASK session. All 29 C‐schools, at the end of the school year, completed a report that describes the activities that were performed and the estimated time allocated to physical activity/PE during the school year (minutes/week)." Judgement comment: Although this study was a cluster‐RCT, it was unclear how closely located the intervention and control schools were and whether intervention teachers had the opportunity to share their teaching approaches. Adherence of the control school to control group conditions was assessed but not reported. Restricted geographical area could mean risk of cross‐contamination. 3 dropout schools were all in the same district
Other bias	Low risk	Judgement comment: None detected

**Table:** Staiano 2012

Methods	Study design: Randomised controlled trial Unit of randomisation: Adolescent stratified by gender Duration of intervention and follow‐up: 10 weeks of intervention Follow‐up: Immediately post‐intervention Unit of analysis: Adolescent Setting: Washington DC, USA Exclusion criteria: BMI < 75th percentile relative to CDC 2000 US reference growth charts Classification of weight status: Overweight: BMI ≥ 85th percentile, obese: BMI > 95th percentile relative to CDC 2000 US reference growth charts Start date: Not reported End date: Not reported	
Participants	N (randomly assigned): 74 (28 in competitive group one, 27 in cooperative group, 19 in control group) N (completed): 54 (19 in each intervention group, 16 in control group) Age range: 15 ‐ 19 years, mean 16.5 years Sex: 57% female Ethnicity: All black Attrition: 27.0% (20/74) Reason for attrition: Self‐consciousness due to obesity, school truancy or dropout; school transfer; lack of interest; pregnancy; safety concerns about walking home in the dark; sports practice time conflicts; academic tutoring time conflicts, frequent headaches and an injury outside of the programme that required crutches. School administrators removed 3 students from the programme because of behavioural infractions external to the exergame intervention	
Interventions	**Comparison: competitive physical activity versus standard practice, co‐operative physical activity versus standard practice** **Interventions:** Nintendo Wii EA Sports Active exergame played in competitive condition individually or in co‐operative condition in pairs for 30 to 60 minutes, 5 days a week, over a period of 10 weeks. "Fitness video game included cardio activities (e.g. inline skating), sports games (basketball, volleyball, tennis, baseball) and strength training. Exergame routine was the same for both intervention groups. Routines varied on a daily basis and gradually increased in difficulty throughout the study." "Children in the competitive group were encouraged to win by earning top scores and expending most calories each time they played. Children in the co‐operative group were encouraged to earn the highest possible score and to expend the most calories as a pair." "Children were supervised during the exergame sessions." Compliance was assessed through attendance. **Standard practice:** Continuation of usual school lunch or after‐school activities or both (Quote: "Control participants continued usual daily activities, such as socializing with friends, tutoring, and sports team practice")	
Outcomes	**Outcome 1: Cognitive function:** Executive function (visual‐spatial skills, response inhibition, motor planning, visual scanning, speed, cognitive flexibility) measured using the subscales Design Fluency and Trail‐Making of the Delis‐Kaplan Executive Function System. Tests were administered by a trained researcher and were coded by 2 research assistants; a 3rd research assistant double‐coded all tests **Outcome 2: Obesity indices:** Body weight change: Body weight measured clothed without shoes by paediatricians and nurse practitioners at the school‐based wellness clinic. Body weight remained unadjusted for height	
Notes	No sample size calculation was performed. Thus, this study might be at risk of a type two error 5 of the study participants (2 boys, 3 girls) were without obesity or overweight. However, this study was done with the intention of weight management, and the number of normal‐weight children is small when allocated into a control group and the 2 intervention groups Participants attended on average 1 exergame session a week Time point of measurement of cognitive function potentially introduced a confounding effect of acute exercise on cognitive function Funding sources: Robert Wood Johnson Foundation, Georgetown University	
***Risk of bias***		
**Bias**	**Authors' judgement**	**Support for judgement**
Random sequence generation (selection bias)	Unclear risk	Quote (from email correspondence): "An adult research coordinator drew a number to randomly assign condition. When conditions became imbalanced due to attrition, new participants were assigned consecutively to the next available condition to maintain sample size balance."
Allocation concealment (selection bias)	Unclear risk	Quote (from email correspondence): "Participants knew that they were assigned to 1 of 2 classrooms or else to the control group, but they did not know the research aim until the disclosure period at the end of the study."
Blinding of participants and personnel (performance bias) All outcomes	Unclear risk	Judgement comment: Blinding not possible in exercise intervention Quote (from email correspondence): Children "did not know the research aim until the disclosure period at the end of the study" Judgement comment: Personnel were also blinded to true purpose of the study (information obtained from email correspondence)
Blinding of outcome assessment (detection bias) All outcomes	Low risk	Quote (from email correspondence): "The coders and data enterers were blinded to the participant's condition"
Incomplete outcome data (attrition bias) All outcomes	High risk	Judgement comment: Analysis was performed with data when both baseline and post‐intervention data were available. Therefore, study did not account for incomplete outcome data. No information available on characteristics of missing data
Selective reporting (reporting bias)	Low risk	Judgement comment: Dissertation was assessed and all previously‐stated outcomes were reported in the article
Comparability of baseline groups	Unclear risk	Judgement comment: No formal assessment performed
Cross‐contamination	Low risk	Quote: "Children were supervised during the exergame sessions. Compliance was assessed through attendance."
Other bias	Low risk	Judgement comment: None detected

**Table:** Sánchez‐López 2017 [pers comm]

Methods	Study design: Cluster‐randomised controlled trial (cross‐over) Unit of randomisation: Schools N schools: 21 (11 intervention, 10 control) Duration of intervention: 1 school year Follow‐up: Immediately post‐intervention (before cross‐over) Unit of analysis: Child Setting: Ciudad Real and Cuenca, Spain Exclusion criteria: Severe Spanish language learning difficulties, serious physical or mental disorders identified by parents or teachers that would impede participation in the programme’s activities; diagnosed with chronic disorders, such as heart disease, diabetes or asthma, which in the opinion of their paediatricians would prevent their participation in the programme’s activities; schools with only one full 3rd‐grade class of preschool or one 1st‐grade class of primary/elementary school Classification of weight status: Not reported Start date: September 2013 End date: June 2015	
Participants	N (randomised): 75 (between‐group distribution unreported) N (analysed): 63 (24 intervention, 39 control) Age range: 4 ‐ 7 years Sex: 63.5% female Ethnicity: South‐European (Spanish) 73%; other 27% Attrition rate: 16% Reason for attrition: Not reported	
Interventions	**Comparison: MOVI‐KIDS programme versus standard practice** **Intervention:** "The Movi‐Kids programme is a multidimensional intervention aimed at influencing individuals and the playground environment." Children participated in an **optional extracurricular, play‐based, non‐competitive physical activity programme:** 3 60‐minute sessions/week using school facilities, adapted to levels of motor competence. "The programme included basic sports games, playground games, dance and other **activities focused on developing motor skills**. At the end of the 1st year, approximately 90 sessions had been carried out in each school." Parents and teachers were involved in activities to promote active lifestyles in their children by "(a) use of reinforcement tools (e.g. a refrigerator magnet with recommendations for physical activity for children); (b) answering a satisfaction‐with‐the programme questionnaire; and (c) access to a blog where parents could observe their children’s progress, read news regarding reinforcing healthy lifestyles, and ask questions of or make complaints to the research team." "Environmental interventions were conducted in the playground. Fixed (a balance circuit and panels with incentives to be physically active during break time) and mobile equipment (tyres of different colours and sizes) were put in the playgrounds to encourage children to be more active during playtime." **Standard practice:** "The standard physical education curriculum (1 hour a week of psychomotor activities to 3rd‐grade preschoolers and 2 hours a week of physical education to 1st‐grade primary/elementary schoolers with physical activity levels at low‐to‐moderate intensity) was applied in both groups."	
Outcomes	**1. School Achievement:** Numerical quantitative concepts and language skills (Analogical relations and Complex verbal orders) assessed using the Battery of General and Differential Aptitudes **2. Cogntive functions**: Basic psychological processes involved in learning, assessed using the Battery of General and Differential Aptitudes scales for children aged 3 – 6 years and 6 – 8 years: general intelligence and visual‐spatial skills **3. Obesity indices:** Measured weight (barefoot and in light clothing) and height (barefoot and upright and with the sagittal midline touching the back board. BMI was calculated as weight in kilograms divided by the square of the height in metres; waist circumference; body fat (triceps skinfold thickness; 4‐electrode Tanita® Segmental‐418 bioimpedance analysis system)	
Notes	Authors provided raw data for characteristic and outcome data for children with obesity or overweight The sample size calculation was based on the total study sample (participants in any weight group) Funding Sources: Ministry of Economy and Competitiveness—Carlos III Health Institute and FEDER funds (FIS PI12/ 00761), Research Network on Preventative Activities and Health Promotion (RD12/0005/0009)	
***Risk of bias***		
**Bias**	**Authors' judgement**	**Support for judgement**
Random sequence generation (selection bias)	Low risk	Quote: "After the approval of school councils, the schools were randomly allocated using the statistical package StatsDirect to either the intervention or the control group."
Allocation concealment (selection bias)	Unclear risk	Judgement comment: No details reported
Blinding of participants and personnel (performance bias) All outcomes	High risk	Quote: "investigators visited each school to explain the aims and methods of the study and to obtain the consent of the head teacher and the school board." Quote: "Through the teachers, a letter was sent to parents inviting them to a group meeting at the school. In this meeting, the objectives, measurements and procedures of the study were explained," Judgement comment: Participants and personnel cannot be blinded due to the nature of this study. The cross‐over design might have exacerbated the risk of performance bias
Blinding of outcome assessment (detection bias) All outcomes	Unclear risk	Quote: "To minimize interobserver variability, the measurements were carried out in the school by trained investigators." Judgement comment: Unclear whether outcome assessors were aware of the group allocation
Incomplete outcome data (attrition bias) All outcomes	High risk	Judgement comment: From email correspondence with the authors: "We have not adjusted for the missing data. The [attrition] between baseline and follow up was 16.2% in all cognitive outcomes."
Selective reporting (reporting bias)	Unclear risk	Judgement comment: The authors provided unpublished data which align with the reported outcomes in the study protocol. However, academic achievement outcomes are not presented in the 2013 trial registry NCT01971840, but they are in the 2015 protocol paper which is after the study had started
Comparability of baseline groups	Unclear risk	Judgement comment: We could obtain no data on whether the experimental groups were comparable at baseline
Cross‐contamination	Low risk	Quote: "In municipalities with more than one school, only one was selected for the study, to avoid contamination of the intervention."
Other bias	Low risk	Judgement comment: None detected

**Table:** Treu 2017

Methods	Study design: Cluster‐randomised controlled trial Unit of randomisation: Schools N schools (randomly assigned): 17 (8 standard arm, 9 enhanced arm); 9 control schools not randomly assigned Unit of analysis: Child Intervention duration: 1 school year Follow‐up: Immediately post‐intervention Setting: East Jackson County, Missouri, USA Classification of weight status: CDC growth charts for overweight and obesity (2000) Start and end date: 2010 ‐ 2011 school year	
Participants	N (randomised): 360 (89 standard arm, 115 enhanced arm, 156 control) N (followed): 349 (85 standard arm, 114 enhanced arm, 150 control) Age range: 8 ‐ 10 years Mean age: Standard intervention 8.7 ± 0.4 years, enhanced intervention 8.7 ± 0.4 years, control 8.7 ± 0.5 years Sex: Standard intervention 45% female, enhanced intervention 50%, female, control 49% female Overweight: Data not available Obesity: Data not available Ethnicity: Standard intervention: White 60%, Hispanic 4%, black 9%, other 8% Enhanced intervention: White 59%, Hispanic 19%, black 13%, other 9% Control: White 31%, Hispanic 15%, black 51%, other 3% Reason for attrition (missing data): Schools did not provide outcome data Attrition rates: Zero for most academic outcomes and very low, with 2% for reading comprehension in the enhanced intervention arm	
Interventions	**Comparisons: Standard intervention versus wait‐list control; Enhanced intervention versus wait‐list control** **Standard intervention: "**Consisted of the Nutrition Detectives (ND) programme and the ABC for Fitness (ABC) programme offered in grade 3. These 2 programmes had already been incorporated into the curriculum and offered annually beginning a few years before the start of this study." **1. Physical activity:** "ABC for Fitness offers **brief ‘bursts’ of physical activity in the classroom**, each of a few minutes in length, spread over the school day. Classroom teachers offered 30 daily minutes of activity bursts throughout the school year. The activity bursts were designed to include a brief warm‐up and cool‐down (e.g. stretching or low‐intensity activity) along with 1 or more core activities of higher intensity (e.g. hopping, running in place, jumping jacks, or dancing to music). Teachers were provided with an ABC manual with guidelines and activity suggestions. The programme structure was flexible and allowed for teachers to be creative in selecting warm‐ups/core activities/cool‐downs, determining the timing and length of individual activity bursts, and deciding how best to incorporate them into the school day (i.e. whether as a break from lessons or incorporated into the lessons)." **2. Health nutrition/lifestyle education:** "ND is a 90‐minute programme, delivered by PE teachers, that aims to convey the link between food choices and health, convince students of the need to become ‘‘supermarket spies’’ to learn the truth about the foods that they eat, and provide ‘‘five clues’’ to distinguish between more healthful (‘‘clued‐in’’) and less healthful (‘‘clue‐less’’) food choices based on the Nutrition Facts labels and ingredient lists on food packages." "At month 3 participants received a 30‐minute booster session." **Enhanced intervention:** Included the ND and ABC programmes plus reinforcements of their messages to participants and their families in the school, home, and a supermarket **1. Physical activity:** As above. "In addition, family‐focused kits were sent home including pedometers, walking tips to increase daily steps, a family log for recording steps, local walking trail guides, walking maps for local grocery stores, physical activity tip sheet, suggestions for ‘activity bursts’, family activity challenge cards, a 3‐minute sand timer to be used for activity challenges, and a log to record the number of activities and repetitions completed." "For each family kit, students were encouraged to return the completed assignments or logs for a small prize. A family night was held in schools and focused primarily on physical activity: families visited stations throughout the building to try out different kinds of exercises, including Frisbee golf and Zumba, and received information or coupons from local fitness‐related businesses." **2. Healthy nutrition/lifestyle education:** As above. "Schools also received articles related to health, nutrition, or physical activity to include in their monthly school newsletters." In addition, "a family‐focused kit was sent home which included a Nutrition Detectives DVD; a reminder card with the programme’s "five clues" to make healthful food choices; grocery store coupons; and a family "homework assignment" to watch the DVD, review the ND clues together, complete an activity applying the clues to foods in the family kitchen, and informational materials on the NuVal Nutritional Scoring System." "A family night was held at the local supermarket, with stations set up to teach families about healthful food choices with games, demonstrations, and taste tests." **Wait‐list control:** "Control schools received a delayed intervention (ND and ABC programmes) during the school year after study completion." Schools were allowed to continue any programming that they would usually offer, which consisted of physical education classes but no classroom‐based programmes involving nutrition education or physical activity."	
Outcomes	**1. School Achievement**: AIMSweb standardised test scores: Maze reading test (reading comprehension); Reading Curriculum‐Based Measurement (reading fluency); Mathematics Concepts and Applications (math problem‐solving skills); Mathematics Curriculum‐Based Measurement (computation performance) **2. Obesity indices:** Measured weight and height fully‐clothed but were instructed to remove shoes and any heavy outerwear such as jackets or sweaters. Calculated BMI z‐scores	
Notes	Authors provided raw data for characteristic and outcome data for children with obesity or overweight The sample size calculation was based on the total study sample (participants in any weight group). Funding source: Health Care Foundation of Greater Kansas City	
***Risk of bias***		
**Bias**	**Authors' judgement**	**Support for judgement**
Random sequence generation (selection bias)	High risk	Quote: "One school district’s 17 elementary schools were randomly assigned to either the SI [standard intervention] group (eight schools) or EI [enhanced intervention] group (nine schools), with the other district’s nine elementary schools serving as the control group." Judgement comment: The districts were not randomised, only intervention arms were randomised.
Allocation concealment (selection bias)	Unclear risk	Judgement comment: No details reported
Blinding of participants and personnel (performance bias) All outcomes	High risk	Judgement comment: Blinding not possible for this type of intervention. The primary aim of the study was obesity prevention and so it was unclear if the participants and teaching personnel were aware of potential effects on academic achievement
Blinding of outcome assessment (detection bias) All outcomes	Low risk	Quote: "These tests are used in schools across the United States as screening and progress monitoring tools." Judgement comment: Assessment seemed centralised and took place for all students in the year group regardless of participation in the study
Incomplete outcome data (attrition bias) All outcomes	Low risk	Quote: "All analyses were based on the intention‐to‐treat principle using the baseline measure carried forward." Judgement comment: Attrition rates were zero for most academic outcomes and very low, with 2% for reading comprehension in the enhanced intervention arm
Selective reporting (reporting bias)	Unclear risk	No reference to a trial register entry or published study protocol
Comparability of baseline groups	High risk	Judgement comment: Comparison of baseline characteristics between the intervention and control schools for the total study sample suggested considerable group differences in ethnicity, BMI z‐scores, physical fitness, nutrition knowledge and mathematical computation skills. Group differences for ethnicity were also present for the subgroup of children with overweight/obesity, based on author‐provided unpublished data
Cross‐contamination	Low risk	Quote: "One school district’s 17 elementary schools were randomly assigned to either the SI group (eight schools) or EI group (nine schools), with the other district’s nine elementary schools serving as the control group." Judgement comment: Separate district for control group
Other bias	Low risk	Judgement comment: None detected

**Table:** Winter 2011

Methods	Study design: Cluster‐randomised controlled trial Units of randomisation: schools (N = 4) Duration of intervention: 24 weeks Follow‐up: Immediately postintervention Unit of analysis: Child Setting: Texas, USA Exlusion criteria: Not reported Classification of weight status: Overweight was defined as BMI 85th to 94th percentile; obesity was defined as BMI > 95th percentile based on gender‐specific CDC BMI‐for‐age growth tables Start date: Not reported End date: Not reported	
Participants	N (recruited): 141 (70 in intervention group, 71 in control group) N (analysed): 125 (61 in intervention group, 64 in control group) Age: 3 ‐ 5 years, mean age: 4.3 ± 0.54 years Sex: 50% female Ethnicity: "predominantly Latino of Mexican American origin" Attrition (children): 27.5% Reason for attrition: None reported	
Interventions	**Comparison: Healthy & Ready to Learn intervention versus standard practice** **Intervention:** Implemented at home and in school by trained parents and teachers. Compliance with the intervention assessed during weekly evaluations at teacher level. Parents interviewed monthly **1. Lifestyle education:** "Parents and teachers read children's books on health‐related themes including nutrition and obesity prevention." **2. Physical activity:** "Teachers and parents were trained to increase children's time spent physically active in moderate to vigorous activity for 60 minutes/d. Activities were play‐based and targeted specific gross motor skills. Physical activity equipment was provided." **Standard practice:** Usual school curriculum and programmes different from the intervention	
Outcomes	**Outcome 1: School achievement:** Receptive vocabulary skills were assessed with the Peabody Picture Vocabulary Test III, administered by trained researchers **Outcome 2 Obesity indices:** Weight and height were measured and BMI calculated.	
Notes	Authors provided raw data for characteristic and outcome data for children with obesity or overweight Funding sources: Baptist Health Foundation of San Antonio and The Max and Minnie Tomerlin Voelcker Fund	
***Risk of bias***		
**Bias**	**Authors' judgement**	**Support for judgement**
Random sequence generation (selection bias)	Unclear risk	Judgement comment: No information provided. Unclear how random sequence was generated
Allocation concealment (selection bias)	Unclear risk	Judgement comment: No information provided
Blinding of participants and personnel (performance bias) All outcomes	High risk	Judgement comment: Blinding to lifestyle education and physical activity intervention was not possible. We could not obtain information whether participants and personnel (teacher and parents) were blinded to the true purpose
Blinding of outcome assessment (detection bias) All outcomes	Unclear risk	Judgement comment: We could not obtain information from study authors
Incomplete outcome data (attrition bias) All outcomes	Low risk	Judgement comment: Study authors provided raw data on the overweight/obese subgroup. For 31 participants, no follow‐up outcome data were available. Review authors imputed missing outcome data using the LOCF method
Selective reporting (reporting bias)	Low risk	Judgement comment: Outcome reported was predefined
Comparability of baseline groups	Low risk	Quote 1 (from report): "Data [...] were matched on the basis of geographical location, size of centre, and demographic characteristics" Quote 2 (from report): "The centre chosen served families that were similar in ethnicity, income and level of parental education" Quote 3 (from report): "Each centre [...] used a common curriculum, teacher professional development, and parent training program"
Cross‐contamination	Low risk	Quote: "The centers were located within a 1‐mile radius of each other in a high‐poverty, low income neighborhood in a large metropolitan city located in South Texas."
Other bias	Low risk	Judgement comment: None detected

**Table:** Wirt 2013 [pers comm]

Methods	Study design: Cluster‐randomised controlled trial Unit of randomisation: Schools stratified based on number of classes in grade 1 or grade 2 or both N randomly assigned: 91 schools (45 intervention, 46 control) N included: 86 schools (44 intervention, 42 control) Duration of intervention: 1 year Follow‐up: Immediately post‐intervention Unit of analysis: Child Geographical region: Baden‐Württemberg, Germany Exclusion criteria: No possibility to collect necessary data at the school, insufficient number of parental consents to collect child’s data Classification of weight status: Overweight: BMI > 90th percentile and obesity: BMI > 97th percentile relative to the German reference population from 1985 to 1999 Start date: Autumn 2010 End date: Autumn 2011	
Participants	N (included): 37 (23 with overweight, 14 with obesity) N (completed): 30 (20 intervention group, 10 control group) N (analysed): 28 (inhibition control), 27 (attention) Age range: 6 ‐ 8 years, mean age: 7.4 ± 0.6 years Sex: 53% female Ethnicity: 52% with migration background Attrition (children): 24.3% Reasons for attrition (for study population with normal weight and overweight): Parental withdrawal from study, change of school, dropout of class from study	
Interventions	**Comparison: 'Join the healthy boat' programme versus no treatment (waiting list)** **Intervention:** Delivered in the primary/elementary school setting (class and recess) by specifically‐trained usual primary/elementary school teachers and at home with parent involvement. Compliance with experimental conditions assessed through evaluation of other health promotion programmes and modifications in school and teaching environment **Healthy lifestyle education:** Healthy lifestyle education of 20 teaching sessions a year focusing on increased physical activity, reduced consumption of sugar‐sweetened beverages and reduced screen time **Physical activity:** 2 physically‐active breaks each school day of 5 ‐ 7 minutes and physical activity task to be performed at home involving parents **Wait‐list control/standard practice: Control schools** followed the regular curriculum	
Outcomes	**Outcome 1: Cognitive function:** Assessment of attention, mental flexibility and inhibition control using the computer‐based test battery of attention for children KiTAP (Kinderversion der Testbatterie zur Aufmerksamkeitsprüfung), administered by trained assessors **Outcome 2: Obesity indices:** 1. BMI percentiles and standard deviation scores calculated on the basis of measured body weight and height. Overweight: BMI > 90th percentile and obesity: BMI > 97th percentile relative to the German reference population from 1985 to 1999. 2. Waist circumference was measured "halfway between the lower costal border and the iliac crest using a metal tape measure"	
Notes	Researchers kindly provided unpublished characteristics and outcome data for children with obesity or overweight Results on both general study sample and overweight/obese subsample have not yet been published Sample size calculation: Calculated for changes of anthropometric variables and running performance for total study sample Funding source: Baden‐Württemberg Stiftung gGmbH	
***Risk of bias***		
**Bias**	**Authors' judgement**	**Support for judgement**
Random sequence generation (selection bias)	Low risk	Quote (from email correspondence): "random sequence generation performed using a computer software"
Allocation concealment (selection bias)	Low risk	Quote (from email correspondence): "Schools were randomised at once"
Blinding of participants and personnel (performance bias) All outcomes	High risk	Quote (from email correspondence): "Children were not informed that the intervention might have a beneficial effect on cognitive function. Teachers, however, were informed that the intervention might improve cognitive function"
Blinding of outcome assessment (detection bias) All outcomes	Low risk	Quote (from email correspondence): "Outcome assessor was blinded to experimental condition"
Incomplete outcome data (attrition bias) All outcomes	High risk	Quote (from email correspondence): "Provided data are from a sub‐sample of the total sample. Missing data were not imputed. Only completed baseline and follow‐up data set were included in the analysis" Judgement comment: No information available on characteristics of missing data
Selective reporting (reporting bias)	Low risk	Judgement comment: Trial authors kindly provided unpublished data Quote (from email correspondence): "Data on mental flexibility cannot be provided to date because test of plausibility has not been performed yet"
Comparability of baseline groups	High risk	Quote (report): "[Stratified] randomisation based on number of classes in grade 1 and/or 2" Quote (from email correspondence): "Baseline groups did not differ in executive function and attention scores, ethnicity and obesity indices. Significant differences were detected for mean age (intervention group 7.22 years; control group 7.74 years) and gender distribution (intervention group: 60% boys; control group: 20% boys)" Judgement comment: Potential risk of comparability of experimental groups at baseline
Cross‐contamination	Unclear risk	Judgement comment: Geographic proximity between experimental groups
Other bias	Low risk	Judgement comment: None detected

BMI: body mass index CAS: Cognitive Assessment System CDC: Centers for Disease Control and Prevention GPA: grade point average IOTF: International Obesity Task Force LOCF: last observation carried forward PA: physical activity PE: physical education

### Characteristics of excluded studies [ordered by study ID]

**Table:**

Study	Reason for exclusion
Bartholomew 2011	Study did not meet intervention criteria: Physical activity intervention was a short bout, 3 days of physically active lessons, which is too short to be considered as a lifestyle intervention for treatment of overweight and obesity
Chaya 2012	Study did not meet the control group criteria: The study used a physical activity control arm
Crova 2014	Study did not aim to prevent obesity (this was confirmed by the study authors on correspondence)
Delgado‐Rico 2012b	Study did not meet study design criteria: It followed a non‐randomised, uncontrolled, pre/postintervention design
Donnelly 2009	Study did not report data for children with obesity or overweight and we were not able to obtain the data from the authors
Donnelly 2013	Study did not report data for children with obesity or overweight and we were not able to obtain the data from the authors
Epstein 2000	Study did not meet control group criteria: All experimental groups received family‐based weight management treatment
Gee 2014	Study obtained cognitive outcomes using self‐ or parent‐reported questionnaire
Gentile 2009	Study did not report data for children with obesity or overweight and we were not able to obtain the data from the authors
Goldfield 2012	Study obtained academic outcomes by self‐reported questionnaire
Grieco 2009	Study did not meet study design and intervention criteria: It followed a non‐randomised, uncontrolled, pre/postintervention design and delivered an acute bout intervention
Gunnarsdottir 2012b	Study did not meet study design criteria: It followed a non‐randomised, uncontrolled, pre/postintervention design
Halberstadt 2017	Study did not meet design criteria: It was a single group before‐after trial
Hill 2011	Study measured outcome during the intervention rather than at baseline and at end of intervention
Hillman 2014	Study did not report data for children with obesity or overweight and we were not able to obtain the data from the authors
Hollar 2010	Study did not meet design criteria: It followed a non‐randomised cluster controlled design
Hutson 2008	Unclear whether study included children with obesity or overweight. Outcome data were not separately reported. Author's contact details not obtainable
Leidy 2013	Study measured school achievement and unrelated cognitive domains (appetite control and satiety regulation) using test tools not specified as eligible in this review (functional magnetic resonance imaging (fMRI) brain activation responses)
Milosis 2007	Primary outcome measure of school achievement was assessed through self‐reported grades
Murray 2008	Study did not report data for children with obesity or overweight and we were not able to obtain the data from the authors
Muzaffar 2014	Study obtained cognitive outcomes using self‐ and parent‐reported questionnaire
Naar‐King 2016	Study obtained cognitive outcomes using self‐ or parent‐reported questionnaire
Pentz 2011	Study obtained cognitive outcomes using self‐ or parent‐reported questionnaire
Pesce 2016b	Study did not aim to prevent or treat obesity (this was confirmed by the study authors on correspondence)
Puder 2011	Study did not report data for children with obesity or overweight and we were not able to obtain the data from the authors
Reed 2010	Study did not report data for children with obesity or overweight and we were not able to obtain the data from the authors
Reed 2012	Study did not meet study design criteria: It followed a non‐randomised, pre/postintervention design
Robinson 2010	Primary outcome measure of school achievement was assessed through self‐reported grades
Salmoirago‐Blotcher 2015	Study obtained cognitive outcomes using self‐ or parent‐reported questionnaire
Smith 2015	Study obtained cognitive outcomes using self‐ or parent‐reported questionnaire
Tomporowski 2008	Study did not meet intervention criteria: Physical activity intervention was a short bout, one‐off session of 23 minutes of treadmill walking, which is not considered a lifestyle intervention for treatment of overweight and obesity
Vanhelst 2012	Study did not meet study design criteria: It followed a non‐randomised, uncontrolled, pre/postintervention design
Verbeken 2013	Study did not meet control group and lifestyle intervention criteria: Control group received same lifestyle intervention as intervention group. Intervention group played a computer game to train executive function, which was not considered an adequate lifestyle intervention according to our definition
Vos 2011	Study did not meet the outcome criteria: Cognitive function was assessed as self‐perceived ability
Wong 2016	Study obtained academic outcomes from self‐report

### Characteristics of studies awaiting assessment [ordered by study ID]

**Table:** NCT02043626

Methods	Study design: Cluster‐randomised controlled trial Unit of randomisation: Schools N schools: 12 (3 for each experimental group) Duration of the intervention: 3 years Follow‐up measurements: Grades 5, 6, 7, 8 Unit of analysis: Child Inclusion criteria: Student of the New Haven School District‐‐State of Connecticut, enrolled in 12 eligible schools participating in study, child between the ages of 9 and 14 or in grades 5 ‐ 8 as of the fall of 2011. Primary language of communication is English Exclusion Criteria: Not enrolled in 1 of 12 participating schools, not in target grade (5 ‐ 8) as of the fall 2011
Participants	Estimated N: 796 Age: 9 ‐ 14 years Gegraphical region: Connecticut, USA
Interventions	Interventions: Group 1 ‐ Physical activity only: Schools will receive educational intervention and increased opportunities for physical activity. District‐wide policies include mandates for daily physical activity and PE and development of policies that prohibit withholding PE for punitive reasons. To further increase physical activity, the School Wellness Policy specifies expanding programmes/activities that meet need, interest, and abilities of students. Exer‐gaming consoles will be provided to 6 schools and will be integrated into 5th ‐ 8th grade PE classes and after‐school programmes. New and innovate gym equipment will be purchased for the 6 target schools for use in gym class and after‐school programming. Various pedometer and interactive programmes encouraging physical activity in and outside of school are planned for the 6th grade Group 2 ‐ Nutrition only: Policy changes will focus on 6 target schools. District will expand nutrition education by integrating other opportunities to learn and practice healthy behaviours across disciplines. Farm‐to‐School programmes will include school visits by farmers to teach students about agriculture, healthy foods and nutrition, coinciding with Farmer's Market Menu Days. Schools will receive 4 45‐minute nutrition workshops a year. Community educators will offer culturally appropriate, interactive nutrition workshops and cooking demonstrations. Cafeterias will receive youth‐friendly nutritional messaging, regular promotion of new menu foods, and a variety of monthly nutrition‐focused activities. The goals are to: increase number of students who try new menu items regularly, increase acceptance of healthy foods, and improve nutrition literacy. Policy states schools will limit celebrations that involve food to no more than 1 per class/month: 6 schools will pilot alternatives to food for celebrations Group 3 ‐ Physical Activity + Nutrition: Schools will receive nutrition education, nutrition standards for foods sold, and opportunities for physical activity. In addition to the above interventions, schools (N+PA) will expand the District's school‐based wellness initiative, PAW‐Physical Activity and Wellness. With District support, PAW schools develop School Wellness Teams (SWTs) to identify school health priorities, implement and sustain health initiatives through school campaigns, promote healthy behaviour, and support wellness policies. SWP addresses health promotion and marketing by limiting product marketing in schools, expanding nutrition education and broadening health communication with parents. In 3 targeted schools, we expand to include Staff Wellness Promotion. Adults in schools are trusted and influential role models for students; by increasing their positive health behaviours, students may be influenced to adopt similar behaviours. The District will work with the City's Employee Wellness Programme to increase school staff participation. Waitlist‐control/standard practice: Schools will receive educational interventions on health topics not related to nutrition or physical activity (i.e. peer relations, sleep, dental care, etc.)
Outcomes	School achievement: Standardised test scores in reading and mathematics Obesity Indices: Body weight and height to calculate BMI
Notes	Retrospective trial registration: 20 January 2014 Completion date: June 2016 No publication of intervention results yet. Unclear if participants were categorised by body weight status We contacted the authors to obtain unpublished data. Funding source: US NIH Grant no 5R01HD070740

**Table:** NCT02122224

Methods	Study design: Randomised controlled cross‐over study Unit of randomisation: Child Duration of the intervention: 4 weeks Follow‐up measurements: Week 1, week 3, week 5 and week 7 Inclusion criteria: 4 ‐ 5 year‐old children attending Head Start centers in Lafayette Exclusion criteria: No digestive disorders, food allergies, or kidney disease. Children cannot be taking medication that would affect appetite
Participants	Estimated N: 80 Age: 4 ‐ 5 years Geographical region: Indiana, USA
Interventions	Interventions: Children rotated through 4 1‐week periods of consuming ad libitum high protein (19 ‐ 20 g protein), high fibre (10 ‐ 11 g fibre), high protein and high fibre (19 ‐ 21 g protein, 10 ‐ 12 g fibre) breakfast Control: Usual breakfast
Outcomes	Cognitive function: Memory after consuming breakfasts with different nutrient content, assessed using the novel object test Obesity indices: Changes in weight/body fat
Notes	Estimated completion date: May 2014 clinicaltrials.gov/ct2/show/study/NCT02122224 No report on intervention effects published yet. Unclear if participants were categorised by body weight status We contacted the authors to obtained further details.

**Table:** Vetter 2015

Methods	Study design: Randomised controlled trial Duration of intervention: 6 weeks Unit of allocation: Not reported Unit of analysis: Child Inclusion criteria: Unclear
Participants	N estimated: 147 (72 intervention, 75 control) Age: Not reported (Grade 3 students)
Interventions	Comparison**:** Classroom mathematics Intervention**:** Playground mathematics ('Shaping Healthy Choices')
Outcomes	School achievement: Numeracy: general competence; times‐table competence Obesity indices: BMI
Notes	Abstract only We contacted the authors, but without response.

### Characteristics of ongoing studies [ordered by study ID]

**Table:** Bau 2016

Trial name or title	**Maintain study**
Methods	Study design: RCT Sequence generation: Participants assigned by study team member by time and date of return using list with random order "intervention" or "control" (principle of contingency) Allocation concealment: Not reported Unit of allocation: Child Blinding: Children: Not reported Providers: Not reported Outcome assessor: Not reported Duration of intervention: 12 months Follow‐up: 12, 18, 24, 36 and 48 months Unit of analysis: Child Inclusion criteria: 1. Age between 10 and 17 years; 2. primary adiposity at recruitment with a BMI exceeding the 97th percentile; 3. willingness of candidates and their families to actively participate in the 3 parts of the study Exclusion criteria: 1. Participation in another clinical trial or intake of experimental medication within 30 days before the inclusion date; 2. personal relationships or dependencies between participants and study team; 3. severe chronic diseases that were incompatible with the planned intervention, i.e. severe damage of liver or kidney, clotting disorder, psychological or psychiatric disorders, systemic infections, endocrine diseases as well as malabsorption, food allergies or special diets; 4. pregnancy
Participants	N (randomised): 137 N (completed): 127 (111 after 18 months, 100 after 24 months, 87 after 36 months & 77 after 48 months) N (analysed): Not reported Age range: 10 ‐ 17 years Sex: 53% female Ethnicity: Not stated Nationality: 49% German, 28% Turkish, 23% other Geographical region: Berlin, Germany
Interventions	**Comparison**: Usual care **Intervention**: Group intervention led by professional therapists who addressed healthy eating and lifestyle factors (10 sessions over 12 months) **Standard care**: No group‐based intervention, received usual medical care. Agreed to complete 1 ‐ 2 physical activity sessions a week in addition to school physical education
Outcomes	**School achievement:** Unclear how assessed **Obesity indices:** Height and weight, waist and hip circumference, bioelectrical impedance analysis
Starting date	October 2009
Contact information	Dr Susanna Wiegand, Charite University, Augustenburger Platz 1, 13353 Berlin, Germany email: susanna.wiegand@charite.de
Notes	Completion date: 2015 We contacted the authors to obtain unpublished data. The authors informed us that the data are still being processed and are not ready for data analysis yet Funding source: German Research Association (Deutsche Forschungsgemeinschaft, DFG – governmental funding) Trial registration: NCT00850629 (clinicaltrials.gov/ct2/show/NCT00850629)

**Table:** Cadenas‐Sanchez 2016

Trial name or title	**ActiveBrains project**
Methods	Study design: Randomised controlled trial Sequence generation: Electronic using SPSS Allocation concealment: Not reported Unit of allocation: Child Blinding: Children: Not reported Providers: Not reported Outcome assessor: Not reported Duration of intervention: 20 weeks Follow‐up: 8 months (in 50% subsample) Inclusion criteria: 1. Age between 8.0 and 11.9 years; 2. with obesity or overweight, based on the sex‐ and age‐specific international body mass index standards (World Obesity Federation); 3. not to have any physical disabilities or neurological disorder that limits exercising; 4. to report no use of medications that influence central nervous system functioning; 5. in the case of the girls, not to have started menstruation at baseline Exclusion criteria: 1. Left‐handedness (measured by the Edinburgh inventory); 2. attention‐deficit hyperactivity disorder (ADHD) evaluated by ADHD rating scale; 3. other psychiatric diagnoses indicated with self‐report
Participants	N (estimated): 100 Age range: 8 ‐ 11 years Geographical region: Spain
Interventions	**Comparison**: Exercise versus wait‐list control **Intervention**: 20 week exercise programme. 5 sessions offered a week (90 minutes/session) of which children are suggested to attend 3 sessions/week **Wait‐list**: No intervention until end of follow‐up period
Outcomes	**Cognitive function**: A battery of tests were used to assess cognitive performance (namely executive function) including KBIT Brief Intelligence Test, the Design Fluency Test, The Stroop Color‐Word Test, The Zoo Map Test, The Trail Making Test, and relational memory tests **School*achievement:*** Used both final school grades obtained before and after the intervention and the Bateria III Woodcock‐Muñoz test (including 3 tests of reading, 2 tests of oral language, 3 tests of mathematics, 3 tests of written language and 1 test of academic knowledge) **Obesity indices:** Height and weight, body mass index, waist circumference and triceps and subscapual skinfolds thickness, body composition, hepatic steatosis, subcutaneous fat and visceral fat (MRI).
Starting date	December 2014
Contact information	Department of Physical Education and Sports, Faculty of Sport Sicences, University of Granada, Carretera de Alfacar s/n, Granada 18071, Spain. Email: ortegaf@ugr.es
Notes	Estimated completion date: July 2017 Funding source: SpanishMinistry of Economy and Competitiveness (Reference DEP2013‐47540) Trial registration: NCT02295072 (clinicaltrials.gov/ct2/show/NCT02295072)

**Table:** DRKS00005275

Trial name or title	**Ballschule ‐ leicht gemacht (Ball School ‐ easy) [Physical exercise and diet counselling for overweight children]**
Methods	Study design: Randomised controlled trial Unit of allocation: Child Blinding: Children: No Providers: No Outcome assessor: Not reported Duration of intervention: 6 months Follow‐up: Immediately post‐intervention Inclusion criteria: Age‐appropriate body weight above the 90th percentile; age between 6 and 10 years old, attending primary/elementary school; exclusion from general diseases after being examined; agreement by a legal guardian for taking part in the study Exclusion criteria: Children with somatic cause of adiposity; relevant somatic disease (no further details provided); regular administration of medication; children with mental retardation
Participants	N (estimated): 120 Age range: 6 to 12 years Geographical region: Heidelberg, Germany
Interventions	4‐arm trial with 3 intervention arms and 1 no‐treatment control group **Intervention:** Arm 1 (Physical activity): Movement therapy for 90 minutes twice a week, aiming to be fun while being physically active and gaining sport‐specific skills. Sessions included behavioural therapy aiming to change daily physical activity Arm 2 (Diet): An optimised mixed diet was implemented in addition to behavioural therapy aiming to change eating behaviour over 8 units of 90 minutes of nutrition counselling, partially together with parents Arm 3 (Physical activity + Diet): In the combined group the children took part in the ball school programme as well as the nutrition counselling, together with their parents. The contents were equivalent to those of the ball school and nutrition group (see Arm 1 and Arm 2). **Control:** The control group did not participate in a special programme during the intervention period of six months
Outcomes	**Cognitive functions:** Differential performance test was used to measure performance during focused activity, the culture fair intelligence test was applied to measure overall intelligence **Obesity indices:** Change in BMI ‐ SDS
Starting date	15 August 2006
Contact information	Institut für Sport und Sportwissenschaft, Im Neuenheimer Feld 700, 69120 Heidelberg, Germany. www.issw.uni‐heidelberg.de
Notes	Retrospective trial registration: 06 March 2014 Completion date: 19 June 2017 Funding source: Günter Reimann‐Dubbers Stiftung; Manfred Lautenschläger‐Stiftung gGmbH Trial registration: DRKS00005275 (www.drks.de/drks_web/navigate.do?navigationId=trial.HTML&TRIAL_ID=DRKS00005275)

**Table:** ISRCTN12698269

Trial name or title	**Effectiveness of the run‐a‐mile intervention**
Methods	Study design: Cluster‐RCT Duration of intervention: Not reported Sequence generation: Not reported Allocation concealment: Not reported Unit of allocation: Not reported Blinding: Children: Not reported Providers: Not possible Outcome assessor: Not reported Duration of intervention: Not reported Follow‐up: 12 months Unit of analysis: Not reported Analysis: Not reported Sample size calculation: Not reported
Participants	N (recruitment target): 40 clusters, 60 participants/cluster Age range: Years 3 and 5 primary/elementary school pupils Sex: Mixed (not yet recruited) Ethnicity: Not reported Inclusion criteria: 1. Children in school years 3 and 5; 2. attending participating schools Exclusion criteria: 1. Disability that prevents children from running/walking a mile a day; 2. unable to have BMI measured Geographical region: Birmingham, UK
Interventions	**Intervention**: Children in intervention schools will work with teachers to map a track within school grounds and calculate how many laps of the track will be equivalent to a mile. Every day, at random times of the day, all children will be encouraged to walk or run this track with the aim of achieving a mile. This is in addition to schools' usual practices **Comparison**: Continue with usual practice
Outcomes	**School achievement:** Teacher assessment. **Obesity indices:** Weight, height and percentage body fat
Starting date	01 November 2016
Contact information	Dr Sandra Passmore; Health Education Service, Services for Education, 10 Edward Street, Birmingham, B1 2RX. sandra.passmore@servicesforeducation.co.uk
Notes	Trial registration: ISRCTN 12698269 (www.isrctn.com/ISRCTN12698269)

**Table:** NCT01737658

Trial name or title	**Insulin resistance and cognitive dysfunction in obese adolescents**
Methods	Study design: Randomised controlled trial Blinding: Children: No Providers: No Outcome assessor: No Duration of intervention: 6 months Unit of analysis: Child Inclusion criteria: Male and female participants 14 to 19 years of age; BMI > 99th centile; clearance by paediatric cardiologist (including evaluation of VO2max) Exclusion criteria: Younger than 14 years of age and older than 19 years; youth with type 1 or type 2 diabetes; serious medical conditions; no clearance by cardiologist Sample size calculation: Not provided
Participants	N estimated: 50 Age: 14 to 19 years BMI > 99th centile corrected for age Geographical region: New York, USA
Interventions	**Comparison:** Exercise versus wait‐list control **Intervention:** Exercise programme, no further details provided
Outcomes	**Cognitive function:** Change in neurocognitive function, no further details provided
Starting date	December 2012
Contact information	Siham Accacha, MD, Pediatric Endocrinology and Metabolism; Assistant Professor Stoney Brook School of Medicine; Principal Investigator, Winthrop University Hospital, Mineola, New York, United States, 11501
Notes	Completion date: February 2016 We contacted the author to obtain data. Authors informed us that they are writing up the data for publication and no data were provided Funding source: Not reported Trial registration: NCT01737658 (clinicaltrials.gov/show/NCT01737658)

**Table:** NCT02873715

Trial name or title	**Primary care pediatrics learning activity and nutrition with families (PLAN)**
Methods	Study design: Randomised controlled trial Blinding: Children: No Provider: No Outcome assessor: Yes Duration of intervention: 2 years Follow‐up measurements: 6, 12, 18 and 24 months Inclusion criteria: The participating children will be between the ages of 6 and 12 and above the 85th percentile for weight and will have at least 1 parent with obesity or overweight (BMI > 25 kg/m^2^). 1 parent must agree to attend all parent/child treatment meetings as the participating parent. For families in which 1 parent is obese, this parent will be encouraged to be the participating parent; if both parents are obese, the family will choose 1 parent to enrol in the study. Similarly, if 2 children in the family are obese, the older sibling will be encouraged to be the primary participant, as it is more likely the younger sibling will model the older sibling. Although only the child who is overweight/obese and the participating parent will be required to attend treatment sessions, all family members living in the household, including other adults and siblings, will be encouraged to participate indirectly by supporting changes in the family's lifestyle. All participants must be able to speak and comprehend English at a first‐grade level Exclusion criteria: The participating parent or child will not be receiving treatment for a DSM‐5 disorder that interferes with treatment delivered as part of the intervention or is explicitly targeted towards management of weight control; will not have a physical disability or diagnosis that prevents performance of physical activity at a level equivalent to a brisk walk or that places severe restriction on diet; will not be on a medication regimen that affects weight; and will not be participating in an alternative weight control programme. Families in which either the participating child or parent is actively involved in psychological or other interfering weight‐loss treatment, using weight‐affecting medications, or has a psychiatric or medical condition (e.g. anorexia nervosa, schizophrenia, binge eating disorder, depression) that would hinder participation in the study will be excluded as identified by the screening assessments described in section 5.B.2. Screening Assessments of the grant text. Families in which the overweight parent is pregnant or is planning on becoming pregnant during the 2‐year study period will be excluded
Participants	N (recruitment target): 1284 Age range: 6 ‐ 12 years Geographical region: New York, USA
Interventions	Comparison: Family‐based treatment plus enhanced usual care versus enhanced usual care **Intervention:** Family‐based treatment uses behaviour‐ change techniques to target family‐wide changes in diet and physical activity habits, with the goal of promoting weight loss and subsequently healthy weight maintenance in all participants. Participants will have visits between 15 to 60 minutes as frequent as weekly and no longer than monthly over the 2‐year study **Control:** Participants will receive the Pediatric Obesity Clinical Decision Support Chart, titled "5210 Every Day!" as the intervention‐ and care‐consistent recommendations by the Expert Committee Recommendations for Assessment and Treatment of Obesity and the American Academy of Pediatrics 2. Participants will meet with their physician for 15 minutes, minimally every 3 months or monthly if needed, over the 2‐year study
Outcomes	**Cognitive function:** Delay of gratification: a computer task about choices assesses the level of immediate gratification parents and children make over treatment using area under the curve (AUC) **Obesity indices:** Height and weight will be taken to calculate changes in overweight status
Starting date	June 2017
Contact information	Colleen K Kilanowski, State University of New York at Buffalo, Telephone: 716‐829‐6816, Email: ckk@buffalo.edu
Notes	Estimated completion date: December 2020 Trial registration: NCT02873715 (clinicaltrials.gov/ct2/show/NCT02873715)

**Table:** NCT02972164

Trial name or title	**Adapted cognitive behavioral approach to addressing overweight and obesity among Qatari youth**
Methods	Study design: Randomised controlled trial Unit of allocation: Unclear Blinding: Children: No Provider: No Outcome assessor: No Duration of intervention: 26 weeks Follow‐up measurements: 26 weeks (obesity indices: week 3, 14, 26) Inclusion criteria: School children of 9 ‐ 12 yrs, at or above 95th percentile of BMI by age using International Obesity TaskForce (IOTF) cut‐off, and parental consent Exclusion criteria: Psychiatric or neurological disorders; learning disability; dyslexia; current or past drug abuse; head injury and psychotropic medication
Participants	N (estimated): 1000 Age range: 9 to 12 years Geographical region: Qatar
Interventions	**Intervention:** 3 phases: 1. Intensive weight loss camps; 2. after‐school clubs as supplement/consolidation; and 3. maintenance through web and social/family support. The intervention involves developing social and emotional competences, promotion of healthy lifestyle, use of activity monitoring devices to promote increased activity and enlisting family to maintain weight loss in the long term. The intervention group receives all programme components: 1. Parent information sessions and orientation; 2. 2‐week intensive weight loss and lifestyle education camp; 3. after‐school clubs over 12 weeks for consolidation (including physical activity and lifestyle education); and 4. wearable sensors and social media modules with parental involvement **Control:** No treatment (usual school routine)
Outcomes	**Cognitive function:** Impulsivity assessed using CANTAB Stop Signal Task system **Obesity indices:** Weight, height, BMI, waist circumference. Change in BMI Standard Deviation Scores (SDS)
Starting date	August 2013
Contact information	Mohamed Ahmedna, PhD, Telephone: +974‐4403‐6559, Email: ahmedna@qu.edu.qa
Notes	Estimated completion date: December 2017 Trial registration: NCT02972164 (clinicaltrials.gov/ct2/show/NCT02972164)

**Table:** Po'e 2013

Trial name or title	**Growing right onto wellness (GROW)**
Methods	Study design: Randomised controlled trial Unit of allocation: Child‐parent dyads Blinding: Children: Not reported Providers: Not possible Outcome assessor: Blinded by group at aggregate level (not at baseline, as participants randomised after baseline measures) Duration of intervention: 3 years Follow‐up: Not reported Inclusion criteria: 1. Child aged 3 to 5 years old; 2. English‐ or Spanish‐speaking; 3. child's BMI ≥ 50% and < 95%; 4. parental commitment to participate in study; 5. consistent phone access; 6. parents' age ≥ 18 years; 7. parents and children must be healthy, without medical conditions necessitating limited physical activity; 8. child completion of baseline data collection, a minimum of 2 diet recall sessions, minimum accelerometry wear time, and at least 90% of survey items completed by the parent within 30 days of child's weight and height measures; 9. recruitment from 1 of 2 Nashville zipcode regions Exclusion criteria: 1. Children who have < 50% BMI or ≥ 95% BMI; 2. children outside the specified age range; 3. families who do not speak English or Spanish; 4. lack of telephone contact; 5. lack of parental commitment to participate consistently for a 3‐year period; 6. parents and/or children with diagnosed medical illness where regular physical activity might be contraindicated; 7. parents/children who do not otherwise meet the eligibility criteria listed in the study population description; 8. incomplete baseline data
Participants	N (recruitment target): 600 parent‐child dyads Age range: 3 to 5 years Geographical region: Tennessee, USA
Interventions	**Intervention**: A tiered intervention approach (with both dietary and physical activity focus) with a 3‐month intensive phase, 9‐month maintenance phase (delivered over the phone), followed by 24 months sustainability phase with monthly engagement opportunities (delivered at local rec centre) **Comparison**: 6 x 45‐minute sessions delivered over 3‐year intervention period following curriculum based on 'Every Child Ready to Read' and 'Parent Involvement Education' curriculum. Newsletter and monthly email and print letters. Also delivered to intervention participants
Outcomes	**Cognitive function:** Developing executive functioning (Carlson's Executive Function Scale for Preschoolers) and IQ (Woodcock‐Johnson III Tests of Cognitive Abilities ‐ Brief Battery) **Obesity indices:** Height and weight to determine BMI trajectory, body fat % (triceps skin fold), and waist circumference
Starting date	Not reported
Contact information	shari.barkin@vanderbilt.edu; Diabetes Research and Training Centre, Vanderbilt University School of Medicine, 2200 Children's Way, Doctor's Office Tower 8232, Nashville, TN 37232‐9225, USA. Tel: +1 615 936 8066.
Notes	Funding source: National Heart, Lung, and Blood Institute, the Eunice Kennedy Shriver National Institute of Child Health and Development and the Office of Behavioral and Social Sciences Research Trial registration: NCT01316653 (clinicaltrials.gov/ct2/show/NCT01316653)

**Table:** RBR‐38p23s

Trial name or title	**Multifocal intervention in obese adolescents: social competence, behavior problems, academic performance and weight reduction**
Methods	Study design: Randomised controlled trial Blinding: Children: No Provider: No Outcome assessor: No Duration of intervention: 3 months Follow‐up measurements: 3 months and 9 months Unit of analysis: Child Inclusion criteria: BMI above the 95th percentile for age, featuring as obese according to the curves of the Center for Disease Control and Prevention (CDC, 2000); between 10 and 19 years old; willingness to participate in all programme activities Exclusion criteria: Psychological disorders; use of medications that could interfere in the variables; physical difficulties that would impede the development of all activities Sample size calculation: Not reported
Participants	Estimated N: 39 Age: 10 to 19 years Geographical region: São Paulo, Brazil
Interventions	**Intervention Group 1:** Involved 20 meetings with the adolescents divided into 16 for the psychological intervention (twice a week), and 4 monthly for the nutritional orientations; 9 meetings with parents, of which 6 bi‐weekly, 2 nutritional meetings (1 in the beginning and the other after 30 days) and also meeting with a physical educator in the 1st week of the intervention, in addition to 36 sessions of physical exercises for all adolescents, conducted 3 times a week **Intervention Group 2:** Involved 4 monthly meetings with adolescents for nutritional orientation, 9 meetings with the parents (6 bi‐weekly with a psychologist, 2 with a nutritionist and 1 with a physical educator) and 36 sessions of physical exercises for adolescents **Control group:** Involved meetings with a nutritionist and a physical educator for the adolescents and parents and physical exercises sessions for the adolescents; no psychological intervention. The psychological intervention contained life experience activities toward the learning of skills such as self‐control, assertiveness, solving problems (including nicknaming and bullying) and reading the context that contribute to the reduction of behavioural problems and to the gain of self‐worthiness skills, such as self‐esteem, self‐efficacy, thereby enhancing social competence. The objective of the parents' counselling sessions was to teach and provide antecedent and consolidated conditions of socially‐acceptable behaviours and diet. The nutritional sessions for adolescents and parents was conducted by a nutritionist and included information with illustrative material suitable for the age, showing food groups and highlighting those that ought to be included or avoided for its high calorific value. The sessions with the physical educator included information about the importance of physical activity for weight loss and overall health, as well as suggestions for games and activities. The adolescents engaged in weekly physical activity sessions and the practice of indoor physical activity (exercise treadmill and stationary bicycle)
Outcomes	**School achievement**: Form of school grades **Obesity indices**: Body mass index curves relative to the CDC 2000 growth charts
Starting date	18 February 2010
Contact information	Graziela Sapienza, Universidade Federal de São Paulo, Rua Capitão Mor Goes e Moraes, 94 02525060 São Paulo Brazil. Telephone: +55(11)30245082, Email: graziela_sapienza@yahoo.com.br
Notes	Retrospective trial registration: 23 April 2013 Data analysis completion: 19 June 2017 Trial registration: RBR‐38p23s (apps.who.int/trialsearch/Trial2.aspx?TrialID=RBR‐38p23s)

**Table:** Robinson 2013

Trial name or title	**Clinic family & community collaboration to treat overweight and obese children (Stanford GOALS)**
Methods	Study design: Randomised controlled trial Blinding: Children: No Provided: No Outcome assessor: Yes Duration of intervention: 3 years Follow‐up measurements: After 1, 2 and 3 years Unit of allocation: School Unit of analysis: Child Inclusion criteria: Children 7 ‐ 11 years of age, BMI ≥ 85th percentile for age and gender on the 2000 CDC BMI reference Exclusion criteria: Child diagnosed with a medical condition affecting growth (e.g. type 1 diabetes, chronic gastrointestinal disease, chronic renal disease, heart condition); pregnancy; taking type 2 diabetes medication; taking medication affecting growth; with conditions limiting participation in the intervention (e.g. physical disability) and assessment (e.g. insufficient English or Spanish reading and writing competency); unable to understand and complete consent forms; intention to move from San Francisco Bay Area within the next 36 months Sample size calculation: Not reported
Participants	Estimated N: 240 Age: 7 to 11 years Geographical region: California, USA
Interventions	**Intervention:** Large‐scale, community‐based, interdisciplinary, multicomponent, multisetting intervention **Physical activity:** Community team sports programme designed specifically for children with obesity or overweight; no further details on duration, intensity, frequency and type of sport reported **Behaviour change:** Behavioural counselling delivered by primary care provider, home‐based family intervention to reduce screen time, alter food/eating environment and promote self‐regulatory skills for eating and activity behaviour change; no further details on duration and frequency provided **Standard care:** Health and nutrition education; semi‐annual home counselling visits, monthly health education newsletter for children and parents/carers, quarterly community‐based evening health lectures
Outcomes	**School achievement:** No details reported **Obesity indices:** Body mass index, waist circumference, triceps skinfold thickness, waist‐to‐hip ratio; no further details provided
Starting date	July 2012
Contact information	Dr Donna Matheson, donna.matheson@stanford.edu, Stanford University, Palo Alto, California, United States 94304
Notes	Estimated completion date: April 2017 Trial registration: NCT01642836 (clinicaltrials.gov/ct2/show/NCT01642836)

**Table:** Sardinha 2014

Trial name or title	**Physical activity and family‐based intervention in paediatric obesity prevention in the school setting (PESSOA project)**
Methods	Study design: Cluster‐randomised controlled trail Sequence generation: Not reported Allocation concealment: Not reported Unit of allocation: School Unit of analysis: Child Duration of intervention: Unclear, possibly 2 years Follow‐up: 2 years post‐baseline
Participants	N (randomised): Not reported N (completed): 1531 N (analysed): 1531 Age range: 12 to 14 years Sex: 49% female Ethnicity: Not reported Inclusion/exclusion criteria: All healthy students that attended the physical education classes were considered eligible to participate Geographical region: Portugal
Interventions	Intervention: 1. Intervention 1 received standard counselling (see standard care) and 90 minutes of weekly physical activity; and 2. intervention 2 received a 90‐minute additional session with health and weight educational programme and physical activity, implementing principles and basic knowledge within the components of physical activity, eating and wellbeing Standard practice: Standard counselling with general information on eating and physical activity
Outcomes	**School achievement:** Assessed using school grades at the end of academic year in mathematics, language (Portuguese), foreign language (English), and sciences. Provided by schools. **Obesity indices:** Participants were weighed to the nearest 0.1 kg and height was measured to the nearest 0.1 cm. BMI was obtained using the Quetelet index and participants were classified as having a normal weight, overweight or obesity, according to the gender‐ and age‐specific criterion‐referenced standards by the International Obesity Task Force
Starting date	September 2010
Contact information	Prof Luis Sardinha, lsardinha@fmh.ulisboa.pt
Notes	Completion date: 1 September 2013 No result report available so far. We contacted the authors twice to obtain data for children with overweight/obesity, no response yet Funding source: Supported by the FCT ‐ Science and Technology Foundation (Portugal). Trial registration: ISRCTN 76013675 (www.isrctn.com/ISRCTN76013675)

**Table:** Scherr 2014

Trial name or title	**Shaping healthy choices**
Methods	Study design: Cluster‐randomised controlled trial Unit of allocation: School (N = 4) Unit of analysis: Child Duration of intervention: 1 academic year plus sustainability activities Follow‐up: Unclear Inclusion criteria: 4th‐grade students. Schools with 30 ‐ 49.9% eligibility for free school meals, minimum of 4 4th‐grade classrooms and absence of a garden currently used for teaching Exclusion criteria: None reported
Participants	N estimated: 490 (n = 252 intervention and n = 238 control) Age: Unclear (4th‐grade students) Geographical region: Califonia, USA
Interventions	I**ntervention:** Multicomponent school nutrition education programme includes nutrition education activities ( Discovering Healthy Choices curriculum, lesson‐integrated cooking demonstrations, school garden, health fair); family and community partnerships; salad bar in school cafeteria; procurement of local produce; and school wellness committee formation and action **Comparison:** Unclear
Outcomes	**Cognitive function:** Critical thinking skills and basic science process skills **Obesity indices:** Height, weight, and waist circumference measures
Starting date	Unclear
Contact information	Sheri Zidenberg‐Cherr, PhD, Department of Nutrition, Center for Nutrition in Schools, University of California, Davis, 1 Shields Ave, Davis, CA 95616; Phone: (530) 752‐3817; Fax: (530) 752‐8905; E‐mail: sazidenbergcherr@ucdavis.edu
Notes	Funding source: University of California Agriculture and Natural Resources Competitive Grant 11‐1018 and US Department of Agriculture Training Grant 2011‐38420‐20082

**Table:** Stanley 2016

Trial name or title	**Jump start**
Methods	Study design: Cluster‐randomised controlled trial Unit of allocation: Early Childhood Education & Care (ECEC) centre Unit of analysis: Child Duration of intervention: 18 months Follow‐up measurement: Not reported Inclusion criteria: Participants ≥ 3 years old at start of intervention; attending a participating ECEC centre ≥ 2 days a week and not starting primary/elementary school education the following year ECEC centres eligible if ≥ 5 participants Sample size calculation: Adjusted for cluster design
Participants	N estimated: 658 (Intervention n = 348 and control n = 310) Age range: 3 ‐ 5 years Geographical region: Wollongong, Australia
Interventions	**Intervention:** Physical activity and motor skill intervention Gross motor development programme: Structured gross motor lessons, which will be facilitated every day for approximately 20 minutes. This component focuses on 1 gross motor skill, across 2 lessons every fortnight for 13 skills. All skill lessons are repeated 3 times over the 18‐month period. The skill experiences are based on fun, interactive and engaging games. Provision of opportunities for children to practise the gross motor skills taught in the Jump In component every day. It provides opportunities for educators to engage with the children in physical activity and encourage the correct performance of the skills. Jump Out is predominantly child‐led, and educators respond to the child’s cues using a variety of intentional teaching methods Promoting physical activity through active ‘energy’ breaks: Music‐based activities designed to break up long periods of sedentary behaviour with high‐energy physical activity. The children and educators will engage in 2 3‐minute songs every day Integrating physical activity with other learning areas: Activities designed to connect learning and movement. This component aims to use movement to enhance the learning experience. This component will be facilitated twice a day using a range of fun and engaging strategies Reinforcing child care programmes with home‐based interventions: Opportunities provided to families to learn about Jump Start and for parents/caregivers to participate in the same activities at home that the children have been participating in at the ECEC centre **Comparison:** Current usual practice
Outcomes	**Cognitive functions**: Behavioural self‐regulation (inhibitory control, working memory, attention focusing) as assessed using a battery of assessment tasks, including the Head‐Toes‐Knees‐Shoulder task, Card sort, Fish and Shark task, Mr Ant task, Not This task, Temperament scale, Approaches to Learning scale. **Obesity indices:** Measuring height and weight and calculating BMI
Starting date	19 January 2015
Contact information	Prof Tony Okely, University of Wollongong, Wollongong NSW 2522, Australia, +61 2 4221 4641 tokely@uow.edu.au
Notes	Funding source: National Health and Medical Research Council Trial registration: ACTRN 12614000597695 (www.anzctr.org.au/Trial/Registration/TrialReview.aspx?ACTRN=12614000597695)

BMI: body mass index

## Differences between protocol and review

The review title changed to specify the intervention types more precisely, and we used person‐first language to remove stigma.

The author team changed from the protocol to this version of the systematic review. Three new authors joined as co‐authors: Josephine N Booth, Yvonne Laird and John J Reilly. Susan Shenkin was not involved in the update of this review.

We revised the wording in the review objectives in the abstract and main text by providing an example of what we mean by cognitive function in brackets. The wording changed from "cognitive function" to "cognitive function (e.g. executive functions)".

We intended from the outset to select studies based on inclusion criteria; however, we did not state this explicitly in the protocol. The intervention criterion for inclusion was that the study aimed to prevent or treat childhood and adolescent obesity as a primary or secondary outcome through lifestyle interventions. The outcome criterion for inclusion was that studies measured school achievement, cognitive function and future success as defined in Types of outcome measures. We clarified that we restricted the review to the aforementioned outcome measures because the same interventions were studied in the same population for different purposes, e.g. change in body mass index, and were published recently in three other Cochrane Reviews.

We stated in the protocol that studies that included some children and adolescents with overweight would be included in the review only when outcomes for children with obesity or overweight were reported separately. Only a few studies investigated the effects of lifestyle interventions on school achievement or cognitive function or both in a paediatric population with overweight; we therefore did not exclude those studies if results for this population group were not reported separately. We made every effort to contact the authors of those studies to obtain data for the subgroup with obesity or overweight, or both.

In the protocol, we stated that we would include controlled trials. We removed this inclusion criterion and considered only randomised controlled trials, as is was recommended by our Cochrane group.

We provided effect sizes for studies that were inappropriate for inclusion in a meta‐analysis. The protocol stated that we would provide a narrative description of study results derived from those studies.

We intended from the outset to consider the 'Risk of bias' item ‘Comparability of groups at baseline’ to assess the extent of the limitation of unclear risk of bias on randomisation on our confidence in the evidence when using GRADE. We did not state this explicitly in the protocol. We did not consider an unclear risk of selection bias as a serious limitation where we rated the 'Risk of bias' item ‘Comparability of groups at baseline’ as low risk of bias. A low risk of bias in ‘Comparability of groups at baseline’ may suggest adequate randomisation, so we have confidence in the evidence. However, where we rated ‘Comparability of groups at baseline’ as being at unclear or high risk of bias, we considered an unclear risk of bias in randomisation as a serious limitation, and therefore downgraded the quality of evidence to reflect our limited confidence in the evidence.

We have added 'Adverse outcomes' as a primary outcome, and classed 'Cognitive outcomes' as an additional primary outcome.

We added a section on 'Summary of findings' to the Methods.

We removed different intervention types from the subgroup analysis and conducted separate comparisons for each intervention type. The increased number of identified studies allowed us to classify 'multicomponent' interventions in more detail.

At the request of the editorial base, we reported Tau^2^ as an indicator of statistical heterogeneity in random‐effect models in additional to the I^2^ statistic.

We did not search Open Grey (previously Open Sigle) and MIT CogNet, as they did not yield any records previously. We did not search the database on Obesity and Sedentary Behaviour Studies (EPPI) because the content had not been updated since the previous searches. We searched Dissertation and Thesis Global instead of the Networked Digital Library of Theses and Dissertations.

We extracted change‐from‐baseline data and entered them in the meta‐analysis, instead of post‐intervention data, where possible.
